# VCF2Dis: an ultra-fast and efficient tool to calculate pairwise genetic distance and construct population phylogeny from VCF files

**DOI:** 10.1093/gigascience/giaf032

**Published:** 2025-04-04

**Authors:** Lian Xu, Weiming He, Shuaishuai Tai, Xiaoli Huang, Mumu Qin, Xun Liao, Yi Jing, Jian Yang, Xiaodong Fang, Jianhua Shi, Nana Jin

**Affiliations:** Institute for Translational Neuroscience of Affiliated Hospital 2 of Nantong University, Center for Neural Developmental and Degenerative Research of Nantong University, Key Laboratory of Neurodegenerative Diseases, Nantong, Jiangsu 226014, China; Key Laboratory of Neuroregeneration, Ministry of Education and Jiangsu Province, Co-innovation Center of Neuroregeneration, NMPA Key Laboratory for Research and Evaluation of Tissue Engineering Technology Products, Nantong University, Nantong, Jiangsu 226001, China; BGI Research, Shenzhen 518083, China; BGI Research, Sanya 572025, China; BGI Research, Shenzhen 518083, China; Institute for Translational Neuroscience of Affiliated Hospital 2 of Nantong University, Center for Neural Developmental and Degenerative Research of Nantong University, Key Laboratory of Neurodegenerative Diseases, Nantong, Jiangsu 226014, China; BGI Research, Sanya 572025, China; BGI Research, Shenzhen 518083, China; BGI Research, Sanya 572025, China; Key Laboratory of Neuroregeneration, Ministry of Education and Jiangsu Province, Co-innovation Center of Neuroregeneration, NMPA Key Laboratory for Research and Evaluation of Tissue Engineering Technology Products, Nantong University, Nantong, Jiangsu 226001, China; BGI Research, Shenzhen 518083, China; BGI Research, Sanya 572025, China; Institute for Translational Neuroscience of Affiliated Hospital 2 of Nantong University, Center for Neural Developmental and Degenerative Research of Nantong University, Key Laboratory of Neurodegenerative Diseases, Nantong, Jiangsu 226014, China; Institute for Translational Neuroscience of Affiliated Hospital 2 of Nantong University, Center for Neural Developmental and Degenerative Research of Nantong University, Key Laboratory of Neurodegenerative Diseases, Nantong, Jiangsu 226014, China; Key Laboratory of Neuroregeneration, Ministry of Education and Jiangsu Province, Co-innovation Center of Neuroregeneration, NMPA Key Laboratory for Research and Evaluation of Tissue Engineering Technology Products, Nantong University, Nantong, Jiangsu 226001, China

**Keywords:** VCF2Dis, p-distance, population phylogeny, VCF

## Abstract

**Background:**

Genetic distance metrics are crucial for understanding the evolutionary relationships and population structure of organisms. Progress in next-generation sequencing technology has given rise of genotyping data of thousands of individuals. The standard Variant Call Format (VCF) is widely used to store genomic variation information, but calculating genetic distance and constructing population phylogeny directly from large VCF files can be challenging. Moreover, the existing tools that implement such functions remain limited and have low performance in processing large-scale genotype data, especially in the area of memory efficiency.

**Findings:**

To address these challenges, we introduce VCF2Dis, an ultra-fast and efficient tool that calculates pairwise genetic distance directly from large VCF files and then constructs distance-based population phylogeny using the ape package. Benchmarking results demonstrate the tool’s efficiency, with rapid processing times, minimal memory usage (e.g., 0.37 GB for the complete analysis of 2,504 samples with 81.2 million variants), and high accuracy, even when handling datasets with millions of variants from thousands of individuals. Its straightforward command-line interface, compatibility with downstream phylogenetic analysis tools (e.g., MEGA, Phylip, and FastTree), and support for multithreading make it a valuable tool for researchers studying population relationships. These advantages meaning VCF2Dis has already been widely utilized in many published genomic studies.

**Conclusion:**

We present VCF2Dis, a straightforward and efficient tool for calculating genetic distance and constructing population phylogeny directly from large-scale genotype data. VCF2Dis has been widely applied, facilitating the exploration of population relationship in extensive genome sequencing studies.

## Introduction

With advances in sequencing technologies and their decreased cost, increasing amounts of large-scale genome sequencing of individuals have been performed, as exemplified by the 1000 Genomes Project, the UK Biobank, and the 3000 Rice Genomes Project [1–3]. These large-scale genome projects generate a large amount of genetic information, including single-nucleotide polymorphisms (SNPs) and insertions/deletions (indels), which is stored in standard Variant Call Format (VCF). These datasets provide a tremendous resource for further exploring genetic diversity. Exploring population structures and relationships are fundamental tasks in evolutionary biology and population genetics, requiring robust methods to infer evolutionary history [4]. Among these methods, distance-based approaches for phylogenetic tree construction, such as neighbor-joining (NJ) and the unweighted pair group method with arithmetic mean (UPGMA), are computationally efficient and utilize evaluated pairwise distances between genomes to construct trees [4–6]. These methods are particularly well suited for analyzing large datasets, including those in VCF format, because they do not require sequence alignment. In contrast, another category of phylogenetic tools, such as RAxML [7], IQ-TREE [8], PhyML [9], and FastTree [10], uses maximum likelihood estimation. These tools rely on substitution models to infer phylogenies and require alignment data as input. This class of methods is more complex and provides more accurate evolutionary inferences, but is generally more computationally intensive. Although capable of handling large sample counts, their applicability is often constrained to gene-level analyses.

Most current tools for constructing population phylogeny from VCF files first convert VCF format into an alignment format (e.g., FASTA and “Phy”) and then employ third-party evolutionary phylogenetic software, such as MUSCLE [11], FastME [12], FastTree [10], IQ-TREE [8], and Phylip [13]. These tools include local pipelines or programs, such as SNPhylo [14], VCF-Kit [15], and VCFToTree [16], and web-based applications, such as SNiPlay3 [17] and CSI Phylogeny [18]. However, alignment-based methods are computationally demanding and are not well-suited for large-scale genotype datasets due to their high resource consumption, including both computational power and memory.

Currently, two programs, VCF2PopTree [19] and fastreeR [20], are commonly used to calculate genetic distance and then construct distance-based population phylogeny directly from VCF files. VCF2PopTree, a JavaScript-based client-side application, calculates the *p* distance and constructs a distance-based phylogeny using either the UPGMA or NJ algorithms. Although this tool requires minimal memory, its scalability is limited because it can only process populations with fewer than 1,500 individuals (as inferred from its source code). Furthermore, it is slow and becomes unresponsive when handling a large input file. FastreeR, an R package, implements calculation of the “cosine” distance and constructs NJ phylogeny using the Java programming language. It needs several functions for users to calculate distance, construct phylogeny, and display trees, making it difficult for researchers who lack advanced programming skills. Furthermore, it is difficult to control memory usage based on Java. Both tools are only able to adopt one input file. Many efficient tools for such distance-based phylogeny reconstruction have been developed [6]. Nevertheless, the distance calculation step remains a major bottleneck, especially when processing large-scale genomic datasets. To address these challenges, we developed VCF2Dis, a command-line tool designed to efficiently calculate a *p*-distance (i.e., the proportion (*p*) of nucleotide sites at which two sequences differ [21], Methods) matrix from single or multiple VCF files with minimal memory consumption (e.g., 0.37 GB for the whole analysis of 2,504 samples with 81.2 million variants) and high computational speed (e.g., 3.48 times and 47.78 times faster than fastreeR and ngsDist, respectively, when calculating the genetic distance for 1,000 individuals with 2 million variants). In addition, it can construct a phylogenetic tree using the UPGMA or the NJ method by calling the external ape package [22], and display the tree using the ggtree package [23]. Since its first release, VCF2Dis has undergone continuous refinement, including running time, and has been cited in many high-quality scientific studies, including studies of population relationships in wheat [24], *Rhesus macaque* [25], lablab [26], and watermelon [27].

## Data Description

To evaluate the performance of VCF2Dis, we used a popular dataset from phase 3 of the 1000 Genomes Project which sequenced the genomes of 2,504 individuals from 26 populations and characterized over 88 million variants, including 84.7 million SNPs and 3.6 million indels [28].

## Findings

### Accuracy and performance of VCF2Dis

**Figure 1:**
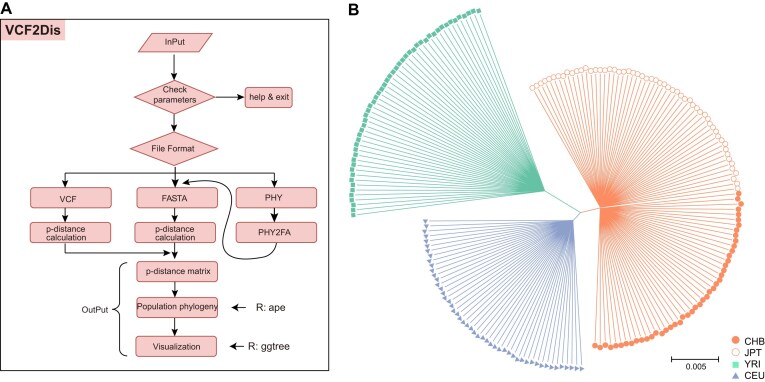
The workflow of VCF2Dis and NJ phylogeny generated from a test dataset consisting 203 samples and 81.2 million bi-allele SNPs isolated from the 1000 Genomes Project. **a**, The VCF2Dis workflow involves several key steps, including parameter checks (e.g., input format), *p*-distance calculation, construction of population phylogeny, and phylogeny visualization. VCF2Dis could adopt input with formats of VCF, fasta and “phy.” The outputs include a *p*-distance matrix, a population phylogeny in Newick format and associated figure. **b**, Neighbor-joining phylogeny of 203 individuals. Colors indicated individuals from distinct populations. YRI, Africa; CEU, European; CHB, China; JPT: Japan.

VCF2Dis is a simple and straightforward command-line tool that enables users to obtain a *p*-distance matrix directly from one or multiple VCF files, and infer distance-based population relationships using the external ape package (Fig. 1a). For the simplest usage, users only need to provide single or multiple input files via the “-InPut” parameter to quickly generate output files, including a *p*-distance matrix, a Newick format tree, and associated figures in PDF and PNG formats. Additionally, users can reconstruct population phylogeny using other alternative phylogenetic software, such as MEGA, Phylip, and FastTree, using the *p*-distance matrix output from VCF2Dis as input. For advanced or customized visualization, annotation, and management of phylogenetic trees, users can upload the Newick format tree to powerful web-based tools, such as iTOL [29] and Evolview [30], or use the ggtree R package [23].

To test its accuracy, we extracted a small dataset from 2,504 human genomes via the parameter “-SubPop,” which contained 203 individuals and 81.2 million variants. The NJ phylogeny of this dataset revealed three distinct groups, with individuals from the same superpopulation (YRI, Africa; CEU, European; CHB and JPT, Asian) clustering together (Fig. 1b). Notably, individuals from China (CHB) and Japan (JPT) were clearly distinguishable. Since its initial release, VCF2Dis has been used in studies investigating population relationships in various organisms, including wheat [24], *R. macaque* [25], lablab [26], and watermelon [27]. These evidences demonstrate the accuracy and utility of VCF2Dis in population genetic research.

VCF2Dis is highly memory-efficient because it processes input files in a line-by-line manner. This approach ensures that memory consumption depends solely on the number of individuals, rather than the total size of the dataset, making it particularly suitable for handling large-scale genotype data. For instance, analyzing 81.2 million variants across 203 individuals required only 0.17 GB of memory. Even when analyzing 2,504 individuals with 81.2 million variants, the memory usage only increased to 0.37 GB, demonstrating that a substantial increase in sample size does not significantly impact memory usage.

VCF2Dis is also exceptionally fast. To speed up runtime, we utilize pointer-based string operations to reduce memory allocation and assignment operation during data parsing. Furthermore, we only calculate the upper-triangle matrix to reduce the computational workload by eliminating redundant operations (Methods and Supplemental Note 1 in Additional file 1). VCF2Dis completed the analysis of 81.2 million variants across 203 individuals in about 3 hours. To accelerate the analysis of large-scale genotype data, we also provide a multiple threading version of VCF2Dis (named VCF2Dis_multi) by paralleling the “for” loop using OpenMP library. We tested the performance of VCF2Dis_multi in a distance calculation step on different thread counts (*n* = 2,4,8,16,32) with a dataset containing 1 million variants across 2,504 samples from the 1000 Genomes Project. The result showed that the runtime generally decreases as the number of threads increases, but the reduction is not perfectly linear (Fig. S1 in Additional file 1). In these tests, the best speed-up was 19-fold, achieved using 32 threads: this took VCF2Dis_multi 8.1 minutes while the single-threaded VCF2Dis took 157.8 minutes (Fig. S1 in Additional file 1 and Table S3 in Additional file 2). We also compared and tested the performance of VCF2Dis and VCF2Dis_multi in the distance calculation step across the number of variants and samples. The runtime of both single-threaded and multi-threaded VCF2Dis exhibited a linear relationship with the number of variants (Fig. S2A in Additional file 1). In this scenario, the multi-threaded version achieved a speed-up of 2–3 times compared with the single-threaded implementation. For the tested sample sizes ranging from 100 to 2,500, the runtime of multi-threaded VCF2Dis demonstrated significant improvement, achieving over an 11-fold speed-up when the sample size exceeded 600 (Fig. S2B in Additional File 1 and Table S2 in Additional file 2). Therefore, the multi-threaded VCF2Dis is highly suitable for analyzing large-scale genomic datasets, particularly those involving thousands of individuals.

### Performance comparison with other existing tools

Two tools, VCF2PopTree and fastreeR, offer functions for pairwise distance calculation and for constructing population phylogeny directly from VCF files (Table 1). However, VCF2PopTree, a JavaScript-based local client program, failed to process datasets with a large number of samples and variants (e.g., 91 samples with 3 M variants). We also found another tool, ngsDist [31], developed in C/C++, which is capable of calculating *p* distance. However, this tool requires an additional preprocessing step—converting VCF format into PLINK format—to function correctly (Table 1). The runtime complexity of VCF2Dis is primarily determined by the distance calculation step (see Methods for details). Additionally, because VCF2Dis focuses on the*p*-distance while tree reconstruction is handled by an external tool, we compared the performance of VCF2Dis, fastreeR, and ngsDist in terms of runtime and memory usage during the distance calculation process. We also consider the effect of the number of samples and the number of variants on the performance (Methods).

**Table 1:** The comparison of VCF2Dis and other distance-based tools.

		Input format					Algorithm		Output			
Software	Programming*	VCF	FASTA	Phy	Multiple input files or list	Sub-population	Distance	Tree	Figure	Distance matrix	Newick tree	Memory
VCF2Dis	C/C++	√	√	√	√	√	*p* distance	NJ,UPGMA	√	√	√	low
VCF2PopTree	JavaScript	√	×	×	×	√	*p* distance	NJ,UPGMA	√	√	√	low
fastreeR	Java	√	√	×	×	×	cosine distance^#^	NJ	√	√	√	high
ngsDist	C/C++	×	×	×	×	×	*p* distance	×	×	√	×	high

* Major programming languages. ^#^defined in the fastreeR.

**Figure 2:**
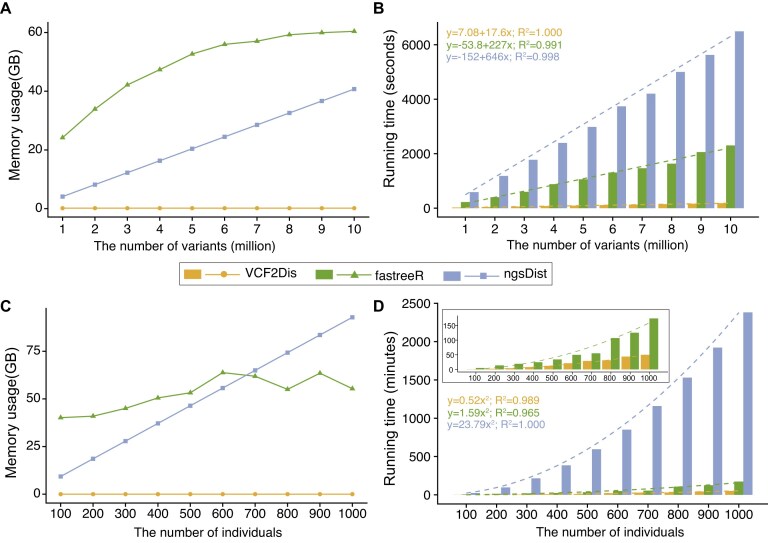
The memory and runtime performance of VCF2Dis, fastreeR, and ngsDist were assessed based on the number of variants and samples used when calculating the genetic distance. **a**, Memory test with an increasing number of variants in a dataset containing 91 samples. **b**, Runtime test with an increasing number of variants in a dataset containing 91 samples. **c**, Memory test with an increasing number of individuals, each containing 2 million variants. **d**, Runtime test with an increasing number of individuals, each containing 2 million variants. The runtime of VCF2Dis and fastreeR are also separately shown in the inset.

In terms of memory usage, VCF2Dis uses very little memory compared with the other two tools (Fig. 2a,c). For example, VCF2Dis required only 10 MB of memory to analyze 1,000 samples with 2 million variants, whereas fastreeR and ngsDist consumed 55.36 GB and 92.83 GB, respectively (Table S1 in Additional file 2). The memory usage of VCF2Dis is independent of the number of variants and increases slightly with the number of samples (Fig. 2). In contrast, the memory usage of fastreeR approximately follows a logarithmic increase with the number of variants and samples, whereas ngsDist exhibits a linear relationship with both the number of samples and the number of variants.

In terms of runtime performance, the runtime of all three tools shows a linear increase with the number of variants. In this situation, VCF2Dis demonstrates the fastest performance, being approximately 12 times and 36 times faster than fastreeR and ngsDist, respectively (Fig. 2b). Regarding the number of samples, the runtime of all three tools approximately follows a pattern where the time taken is proportional to the square of the sample size. However, VCF2Dis showed the fastest performance, being approximately 3 times and 45 times faster than fastreeR and ngsDist, respectively (Fig. 2D). For instance, when analyzing 1,000 individuals, VCF2Dis took 49.84 seconds, while fastreeR took 173.64 seconds, and ngsDist took 2,381.68 seconds, which is approximately 3.48 times and 47.78 times faster (Table S1 in Additional file 2), respectively. Therefore, VCF2Dis consistently outpaced fastreeR and ngsDist, particularly as the sample size increased.

Unlike VCF2Dis, which uses the *p*-distance method, fastreeR employs a “cosine” distance metric. To compare the accuracy of these two softwares, we conducted a test using 203 individuals with 3,492 variants from the 1000 Genomes Project which was included as a test dataset used in VCF2PCACluster software [32]. Our results showed that both tools produced identical distance values. Overall, these comparisons highlight the accuracy and high performance of VCF2Dis in handing large-scale population genetics analyses.

## Discussion

VCF2Dis is a simple and efficient tool designed to facilitate the calculation of genetic distance and the reconstruction of population relationships directly from large VCF files, offering significant advantages for large-scale genomic studies. Since its first release, it has been widely applied and cited in studies of population relationships, such as wheat [24], *R. macaque* [25], lablab [26], and watermelon [27]. One of the key strengths of VCF2Dis lies in its ability to calculate the *p* distance quickly with extremely low running memory, even for large datasets involving thousands of individuals. This is especially useful given the increasing size of population genomic datasets generated by projects such as the UKB whole-genome sequencing (WGS) consortium and other large-scale sequencing efforts [2, 33]. The integration of multithreading further enhances the performance of VCF2Dis, providing significant time savings in computationally intensive tasks, as demonstrated by its 19-fold speed improvement over single-threaded execution in our benchmarking tests of 2,504 samples with 1 million variants using 32 threads. It is important to note that the speed-up achieved by VCF2Dis_multi is often nonlinear compared to the single-threaded version of VCF2Dis. Factors such as the overhead of thread management, uneven workload distribution among threads, and the fact that not all steps (e.g., input/output) in the process are fully parallelizable can impact parallel efficiency. Consequently, we recommend using the multi-threaded version of VCF2Dis for studies involving thousands of individuals, because it provides substantial computational advantages.

In addition to its efficiency, VCF2Dis offers flexibility. The output files, including *p*-distance matrices and the Newick format tree, can be easily used as inputs for other popular phylogenetic analysis tools such as MEGA [21], Phylip, and FastTree, allowing users to build and refine their phylogenetic tree with a variety of software. Moreover, for users who require more advanced visualization and annotation capabilities, compatibility with tools such as iTOL, Evolview, and the ggtree R package provides extensive options for tree manipulation and display.

However, some limitations should also be considered in future work. First, VCF2Dis is highly effective for generating *p*-distance matrices and its utility is dependent on the quality of the input VCF data. In cases where the VCF contains missing or erroneous data, the resulting distance matrix and phylogenetic tree may not accurately reflect the true population structure. Second, the current version of VCF2Dis focuses solely on *p* distance, which may not be the best metric for all phylogenetic analyses. Future incorporation of additional genetic distance metrics could expand the functionality of VCF2Dis and enhance its applicability to a broader range of evolutionary studies. Third, future developments of VCF2Dis could also address user needs for more interactive features, such as a graphical user interface, which would lower the entry barrier for non-technical users. Although VCF2PopTree was not included in the performance comparison due to its failure in most tests, its user-friendly interface, which requires just one click, makes it a viable option for scientific experts without advanced computational skills, particularly for the analysis of small datasets.

In conclusion, VCF2Dis provides a valuable tool for researchers conducting large-scale population genetic studies, offering a fast, flexible, and user-friendly solution for generating *p*-distance matrices and constructing population phylogenies from VCF files. It enables users to infer distance-based population phylogeny directly from VCF files, significantly streamlining the workflow. Despite some limitations, it remains a powerful option for users seeking to streamline their phylogenetic analysis workflows.

## Methods

### Overview of VCF2Dis workflow

VCF2Dis (RRID:SCR_022513) is implemented in the C/C++ and R programming languages, and runs on Linux/Unix and MacOS operating systems. The C/C++ components are mainly used for computational tasks, while R is utilized for generating visualizations (Fig. 1a). We have also provided both Docker and Singularity containerized versions of VCF2Dis, enabling users to bypass the compilation and installation process for a seamless experience. VCF2Dis can utilize compressed or uncompressed input files with formats of VCF, fasta, and “phy,” via “-InPut” and “-InFormat” parameters. Users can provide one or several input files separated by a space or provide a list file with path of input files via the “-InList” parameter. Specifically, VCF2Dis can analyze bgzipped/gzip VCF files which allows random access and is widely used in big genomic data storage and search. By default, VCF2Dis performs calculation for all samples defined in the input. Recognizing the common need in population genetics to construct phylogenies for specific subpopulations, we provide the “-SubPop” parameter. This feature enables users to easily generate trees for selected sample subsets by specifying them through this parameter. To input “phy” format, it is first converted into FASTA format and the *p* distance is then calculated. VCF2Dis uses an external R package, ape [22], to construct population phylogeny, and users could choose NJ or UPGMA algorithms via the “-TreeMethod” parameter. To meet the requirement of showing bootstrap values on the branch of phylogeny for some users, we also used a method of sampling with replacement. For this scenario, users can randomly set a certain ratio (default, 0.25) of all the sites via the parameter “-Rand,” and run VCF2Dis a given number of times (e.g., 100) to separately construct trees. After that, trees are combined and subject to the fconsense program implemented in the PHYLIPNEW package [34] to construct a consensus tree with bootstrap values. In addition, VCF2Dis uses another R package, ggtree [23], to provide an initial display of population relationship. Users can optionally provide prior group information of individuals for color labelling in the tree figure via the “-InSampleGroup” parameter. The outputs of VCF2Dis include *p*-distance matrix, phylogeny in Newick format, and related figures in PDF and PNG formats. Having output the *p*-distance matrix, users can use other phylogenomic software to reconstruct the population phylogeny, such as MEGA (RRID:SCR_000667) [21], FastMe 2.0 [12], Phylip (RRID:SCR_006244) [13], and the PHYLIPNEW package [34]. For advanced and customized visualization of the phylogeny, users can set additional attributes (e.g., layout, color, shape) and modify our provided custom R script for tree display, or use other alternative excellent online or local interactive tools, such as iTOL [29], Evolview [30], and MEGA [21].

### The *p*-distance calculation

The *p* distance is a straightforward approach to estimate genetic distance between two genomes [21]. For genotyping data, the following formula is used to calculate the distance (${D_{ij}}$) for individuals *i* and *j* with the total length of *L*, where variants can be identified:


\begin{eqnarray*}
{D_{ij}} = \,\,\frac{{\mathop \sum \nolimits_{l = 1}^L {d_l}}}{L}
\end{eqnarray*}

For instance, assuming alleles at position *l* are A/C, ${d_l}$ could be set as follows:

If genotypes of two individuals are the same (AA, CC, or AC) then ${d_l}$ = 0
If genotypes of two individuals are AA and AC, respectively, then ${d_l}\,\, = \,\,0.5$
If genotypes of two individuals are AA and CC, respectively, then ${d_l}\,\, = \,\,1$.

Most genetic distance calculation tools, e.g., Vcf2popTree and PLINK, only consider biallelic variants. However, multiallelic variants are frequently encountered in populations and ignoring them could lead to loss of effective genetic information. Thus, we did not perform any preprocessing of VCF files and just compared their genotypes. We adopt a site-by-site pairwise distance calculation and summed the genotypes to produce the total dissimilarity of the whole genome, namely, the pairwise distance matrix, which we then use in an external phylogenetic software (ape) to construct the population phylogeny. Furthermore, VCF2Dis also considers genotype data from phased genomes. In phased genomes:

If genotypes of two individuals are AC and AC, respectively, then ${d_l}\,\, = \,\,0$
If genotypes of two individuals are CA and AC, respectively, then ${d_l}\,\, = \,\,1$.
See Algorithm 1 for the pseudocode of the *p*-distance calculation and see Supplementary Note 1 for more details.

### Accelerated methods of VCF2Dis

Large-scale genome sequencing projects generate millions of variants across hundreds of accessions, leading to extensive memory usage and long runtimes. For instance, the popular tool PLINK (v.1.9) [35] can require more than 257 GB of memory when analyzing a large dataset containing 78 million biallelic SNPs across 2,500 human genomes [32], which is challenging to run on a standard computer. To address memory concerns, VCF2Dis adopts a streaming processing approach, reading and calculating data line-by-line rather than loading the entire VCF file into memory before processing. This method enables efficient handling of large datasets with minimal memory usage (e.g., less than 0.1 GB for analyzing 2,500 individuals in the distance calculation step). To accelerate its runtime, we have made two major improvements during data processing. First, we utilized pointer-based string operations, reducing the overheads associated with memory allocation and assignment operations during data parsing. This results in faster extraction of relevant fields from input files compared with traditional string manipulation methods. Second, we optimized the computation process by employing upper-triangle calculations, which significantly reduce the computational workload by eliminating redundant operations. These optimizations ensure that VCF2Dis is both faster and more memory efficient. See Algorithm 1 for the pseudocode of the accelerated methods and see Supplementary Note 1 for more details. In addition, we also implemented a multiple-thread version of VCF2Dis (VCF2Dis_multi) by paralleling the “for” loop using the OpenMP library [36].

**Algorithm 1:** The calculation of *p* distance and the main accelerated methods in VCF2Dis.

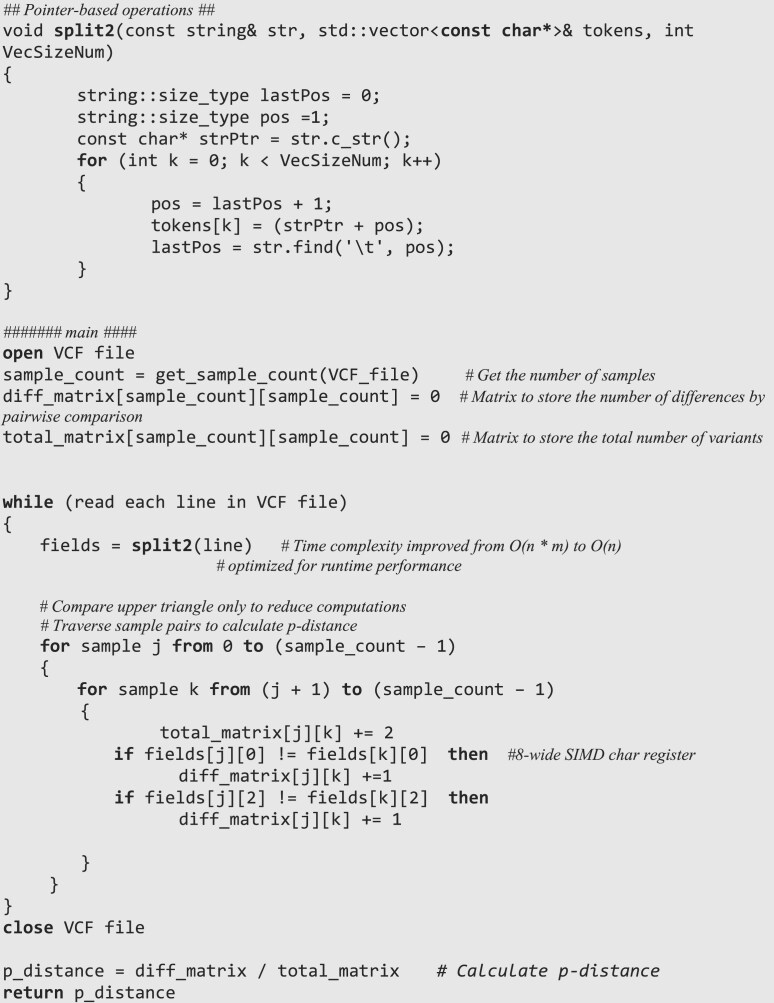

### The runtime complexity of VCF2Dis

The runtime complexity of VCF2Dis is primarily determined by two main components: *p*-distance matrix calculation and tree construction. The *p*-distance matrix calculation step has a complexity of ${\mathrm{{\mathrm{O}}}}( {{n^2}\,\,m} )$, where $n\,\,$ represents the number of samples and *m* represents the number of variants. Each pair of samples requires a comparison across *m* variants. The tree construction step uses a NJ method with a complexity of ${\mathrm{{\mathrm{O}}}}( {{n^3}} )$ because it involves iterative clustering of *n* samples. The overall runtime complexity is therefore $O( {{n^2}\,\,m} ) + O( {{n^3}} )$. Given that *m* (commonly >10^6^) is typically much larger than *n* (commonly <10^3^), the runtime complexity is predominantly determined by the *p*-distance matrix calculation step, making it is nearly $O( {{n^2}\,\,m} )$ for VCF2Dis in practical scenarios.

### Evaluation of performance in memory usage and runtime of existing tools

To evaluate performance, we assessed the memory usage and runtime of existing tools, VCF2Dis, fastreeR, and ngsDist, which are designed for calculating genetic distance and/or reconstructing distance-based population phylogeny. fastreeR was installed via the Bioconductor package, while ngsDist was downloaded from its GitHub repository [37]. Test datasets were generated from the 1000 Genome Project. To evaluate the number of samples on performance, we used a dataset containing 2 million variants across 2,504 individuals from the 1000 Genome Project. However, fastreeR was unable to complete the calculations within a reasonable timeframe, while ngsDist consumed excessive memory resources and was terminated by the system when processing datasets with more than 1,000 samples. Consequently, we conducted performance tests on datasets with fewer than 1,000 samples (100, 200, 300, …, up to 1,000), each containing 2 million variants. To evaluate the effect of the number of variants on performance, datasets were created with fixed 91 samples, containing 1 million, 2 million, 3 million, …, up to 10 million variants each. The tools were executed according to their respective documentation, and the memory usage and runtime of completed jobs were recorded. Results were visualized using the ggplot2 package and are shown in Additional file 2. All evaluations were performed on a computational node with 64 cores and 512 GB of memory, managed using the qsub job scheduler.

## Availability of Source Code and Requirements

Project name: VCF2Dis

Project homepage: https://github.com/hewm2008/VCF2Dis

Operating systems(s): Linux/Unix, MacOS

Programming language: C/C++, R

License: MIT License

RRID:SCR_022513

VCF2Dis requires minimal external dependencies, making installation simple. It can generate the *p*-distance matrix without R or related packages although the visualization features will not be available in this case.

## Supplementary Material

giaf032_Supplemental_Files

giaf032_GIGA-D-24-00393_Original_Submission

giaf032_GIGA-D-24-00393_Revision_1

giaf032_GIGA-D-24-00393_Revision_2

giaf032_Response_to_Reviewer_Comments_Original_Submission

giaf032_Response_to_Reviewer_Comments_Revision_1

giaf032_Reviewer_1_Report_Original_Submission Ekaterina Noskova -- 10/8/2024

giaf032_Reviewer_1_Report_Revision_1 Ekaterina Noskova -- 1/24/2025

giaf032_Reviewer_2_Report_Original_Submission Catia Vaz -- 11/8/2024

giaf032_Reviewer_2_Report_Revision_1 Catia Vaz -- 1/28/2025

giaf032_Reviewer_3_Report_Original_Submission Sankar Subramanian -- 11/13/2024

## Data Availability

The datasets used in this study are freely available from the 1000 Genome Project—Phase 3 dataset [3, 38]. All scripts for the tests, including downloading, generating small datasets, and running, are available in the GitHub repository [39]. An archival copy of the code is available via Software Heritage [40].

## Additional Files

**Additional file 1: Figure S1**. The performance of multithreaded VCF2Dis (VCF2Dis_multi) with different thread counts**. Figure S2**. The performance of multithreaded VCF2Dis (VCF2Dis_multi) with different number of variants and samples compared to the single-threaded VCF2Dis. **Supplementary Note 1**: The pseudocode for improving memory and runtime of VCF2Dis.

**Additional file 2: Table S1**. The performance comparison of the distance calculation step using VCF2Dis, fastreeR, and ngsDist was conducted across varying numbers of variants and individuals. **Table S2**. The performance comparison of the distance calculation step between VCF2Dis_multi and VCF2Dis was conducted across varying sample sizes and numbers of variants. **Table S3**. The performance of the distance calculation step in the multithreaded version of VCF2Dis (VCF2Dis_multi) was evaluated using different thread counts.

## Abbreviations

VCF, Variant Call Format; VCF2Dis, Variant Call Format to distance; SNP, single-nucleotide polymorphism; indel, insertion/deletion; NJ, neighbor-Joining; UPGMA, unweighted pair group method with arithmetic mean.
