# Supplementary material for: VCF2Dis: an ultra-fast and efficient tool to calculate pairwise genetic distance and construct population phylogeny from VCF files
Source: Gigascience. 2025 Apr 4;14:giaf032. doi: 10.1093/gigascience/giaf032 (PMC11970368; doi:10.1093/gigascience/giaf032)
Supplement: giaf032_Supplemental_Files [file giaf032_supplemental_files.zip › Addtionally_File1-revise.docx]

**
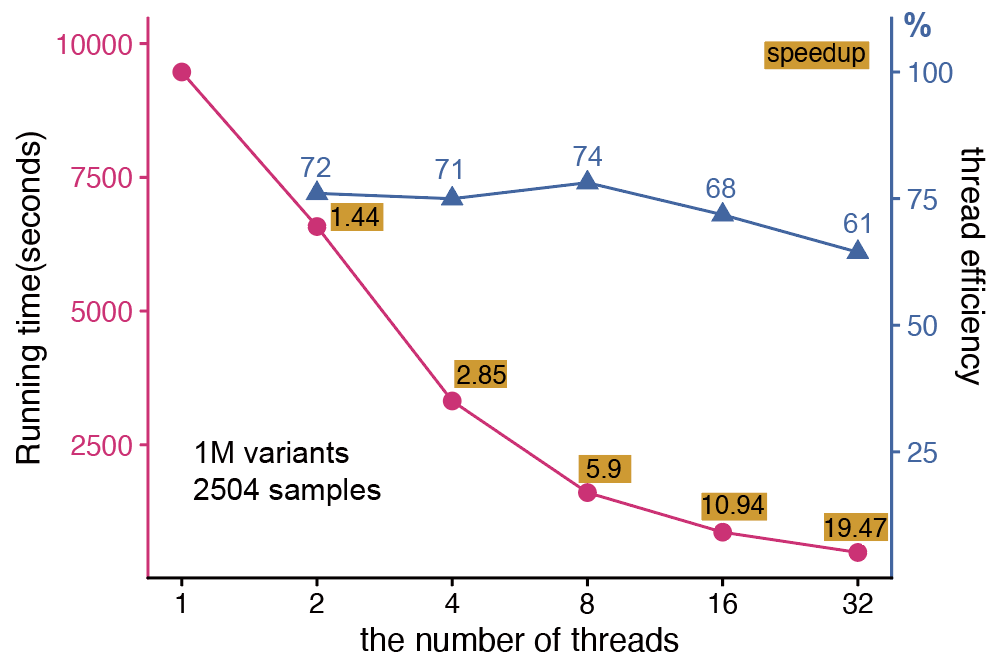
**

**Figure S1. The performance of multi-threaded VCF2Dis (VCF2Dis_multi) with different thread counts**. The tests were performed on single-threaded mode VCF2Dis and multi-threaded VCF2Dis (threads=2,4,8,16,32). The left y-axis indicates the runtime and the right y-axis indicate thread efficiency. The fold of speedup was shown in yellow shaded texts. The thread efficiency was calculated as the ratio of speedup to the number of threads. The runtime was recorded for the distance calculation step.


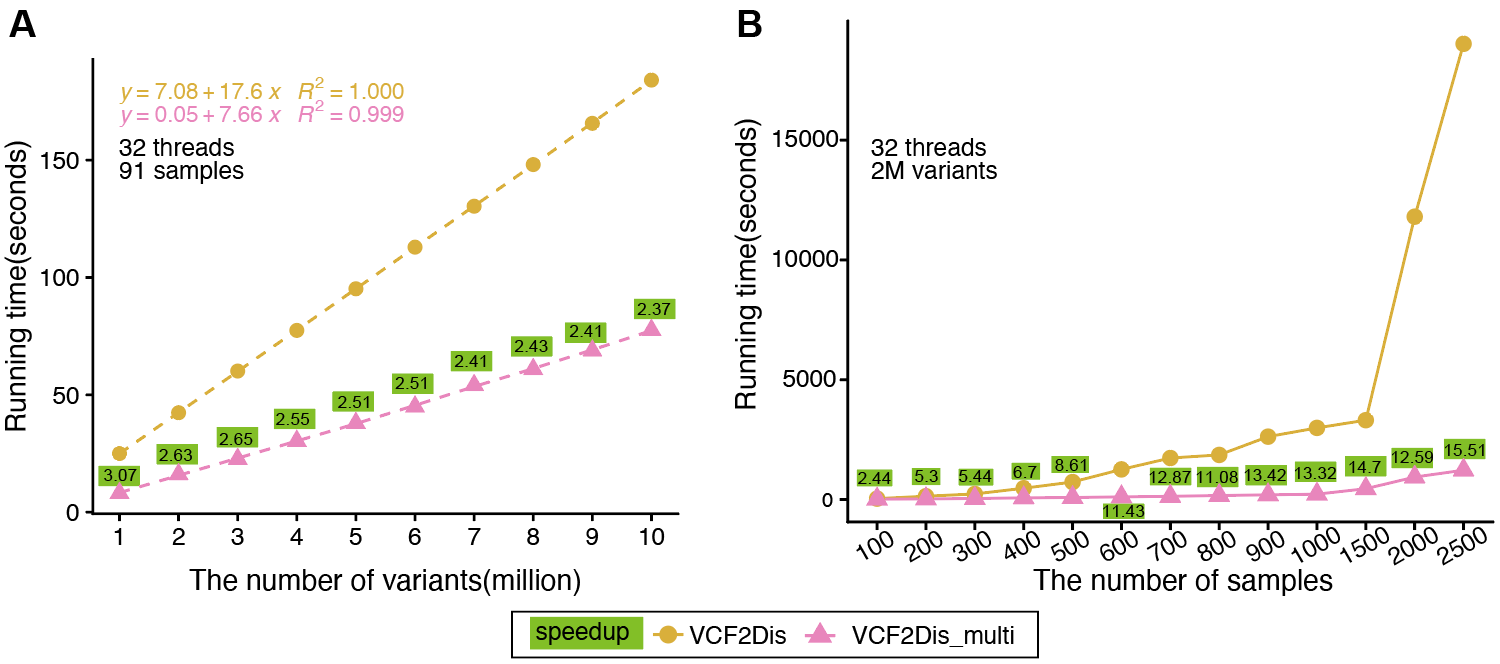


**Figure S2. The performance of multi-threaded VCF2Dis (VCF2Dis_multi) with different number of variants and samples compared to the single-threaded VCF2Dis. A**, The runtime of VCF2Dis and VCF2Dis_multi (32 threads) by analyzing 91 samples with different number of variants. Dashed lines indicate linear fitting. **B**, The runtime of VCF2Dis and VCF2Dis_multi (32 threads) across different number of samples with fixed 2 million (M) variants. Yellow points indicate analysis using VCF2Dis. Pink points indicate analysis using VCF2Dis_multi. Text with green shade indicate speedup. Speedup = Time(VCF2Dis)/Time(VCF2Dis_multi). The runtime was recorded for the distance calculation step.

**Supplementary Note 1: Pseudocode for improving memory and runtime of VCF2Dis**We have included pseudocode below to enhance clarity and facilitate understanding of the approach, emphasizing the optimizations that improve the efficiency of VCF2Dis. A concise version of the pseudocode is presented in the manuscript.

*// Initialize variables*

file INVCFB

string line

integer sampleNumber

array SampleSite[sampleNumber]

array spline[VecSizeNum]

matrix DiffData[sampleNumber][sampleNumber] *//the number of differential variants*

*// Only related to samples, not sites, reducing memory usage*

matrix SumData[sampleNumber][sampleNumber] *//the total number of validated variants*

*// Open VCF file*

open file INVCFB

*// Main processing logic*

while file not end:

line = read a line of data *// Read one line from the VCF file, reducing memory usage*

***// Split the current line into spline array will take much time***

spline = **split2**(line)  *// Use split2 for better performance,time[ O(n * m) 🡪 O(n) ] ， improve run speed*

*// see more at the Below*

*// Traverse sample pairs to calculate p-distance*

for each sample jj from 0 to sampleNumber - 1:

if spline[SampleSite[jj]][0] is '.': *// Skip missing data*

continue

*// compare 8-byte* ***char*** *faster than compare 32-byte* ***int****, improve run speed*

for each sample kk from jj + 1 to sampleNumber - 1:  *// Only compare upper triangle to reduce computations, improve run speed*

if spline[SampleSite[kk]][0] is '.': *// Skip missing data*

continue

*// Update difference counts and total comparisons*

if spline[SampleSite[jj]][0] != spline[SampleSite[kk]][0]:

DiffData[jj][kk] += 1 *// Bases differ, increase difference count*

if spline[SampleSite[jj]][2] != spline[SampleSite[kk]][2]:

DiffData[jj][kk] += 1  *// Coverages differ, increase difference count*

SumData[jj][kk] += 2 *// Each comparison adds 2 (base and coverage)*

*// Close file*

close file INVCFB

// other

p_dis= DiffData/ SumData

This pseudocode shows the optimizations and improvements in memory usage and runtime:

1. **Speed Enhancement with Pointer Operations by defining a function “split2”**

In comparison to the previous version (“split” in V1.36), we have achieved a significant speed improvement by implementing the “split2” function, which leverages pointer-based operations for string processing. This optimized approach reduces overhead by directly manipulating memory, enabling faster and more efficient data parsing. The split2 function operates by iterating over strings using pointers to delimiters, minimizing intermediate object creation and redundant memory allocation.

By replacing split with split2, the processing speed has improved significantly, particularly when handling large-scale datasets or files with high complexity. This improvement positions the new version of VCF2Dis as a more robust and high-performance tool for calculating genetic distances and processing genomic data.

**Time Complexity** Comparison between **split** and **split2**

**Split function**

***Time Complexity:***  **O(n * m)**

n: Length of the input string

m: Number of delimiters in the string

***Explanation:*** The split function typically iterates through the entire string and identifies delimiters to split the string into parts. For each delimiter found, it creates a new substring, which involves memory allocation and copying operations.

**split2 (pointer-based optimization):**

***Time Complexity:*** **O(n)**

n: Length of the input string

***Explanation:*** *b*y using pointer operations, split2 can process the string in a single pass without repeatedly allocating memory or copying substrings. This makes it more efficient in terms of both time and space.


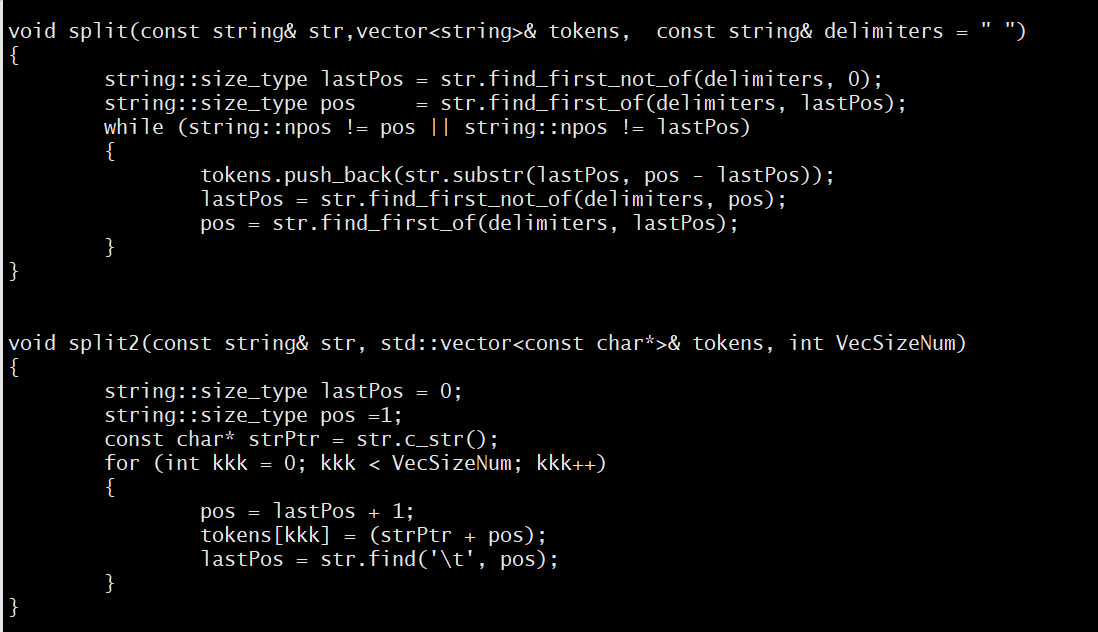
Source code for implementation of split and split2 functions.

Comparison of features between split and split2 functions

| **Feature** | **split (V1.36)** | **split2 (V1.53)** |
| --- | --- | --- |
| Methodology | String-based operations | Pointer-based operations |
| Overhead | High, due to object copies | Low, direct memory access |
| Memory Allocation | Frequent | Minimal |
| Execution Speed | Slower | Faster |
| Scalability | Limited for large datasets | Improved for large datasets |
| time complexity | O(n * m) | O(n) |
| Applicable scenarios | Suitable for situations where there are many delimiters or the delimiters are not fixed and short strings | Suitable for cases where the delimiter is fixed (such as tab) suitable for processing large files and long strings |
| Advantages | Ease of use, compatibility, flexibility | Performance, memory efficiency, scalability |

**B. Memory Efficiency through Streaming Processing**

Another key enhancement in VCF2Dis is the adoption of streaming processing, which enables data to be read and computed on-the-fly rather than loading the entire VCF file into memory prior to processing. By processing data incrementally, this method significantly reduces memory usage.

The memory requirements are now proportional only to the number of sites being processed at any given time, rather than the size of the entire dataset. This optimization is especially advantageous for handling large-scale VCF files, as it ensures that the memory footprint remains minimal regardless of the dataset size. Consequently, users can efficiently process datasets that would otherwise exceed memory limits on standard computing resources.

**C. Computational Efficiency with Upper-Triangle Calculations**

Furthermore, we have optimized the computation process by employing upper-triangle calculations, which significantly reduce the computational workload by eliminating redundant operations. Instead of calculating pairwise distances for the entire symmetric matrix, this approach focuses only on the upper triangle, effectively halving the number of required computations.

This optimization minimizes unnecessary calculations, thereby improving processing speed and reducing resource usage.

Together with other enhancements, such as streaming processing and pointer-based operations, these optimizations ensure that VCF2Dis is both faster and more memory-efficient. As a result, the tool is well-suited for analyzing large-scale datasets with enhanced performance and reduced computational demands.
