# Supplementary material for: VCF2Dis: an ultra-fast and efficient tool to calculate pairwise genetic distance and construct population phylogeny from VCF files
Source: Gigascience. 2025 Apr 4;14:giaf032. doi: 10.1093/gigascience/giaf032 (PMC11970368; doi:10.1093/gigascience/giaf032)
Supplement: giaf032_GIGA-D-24-00393_Original_Submission [file giaf032_giga-d-24-00393_original_submission.pdf]

GigaScience

# VCF2Dis: an ultra-fast and efficient tool to calculate pairwise genetic distance and construct population phylogeny from VCF files.

--Manuscript Draft--

| | |
|---|---|
| Manuscript Number: | GIGA-D-24-00393 |
| Full Title: | VCF2Dis: an ultra-fast and efficient tool to calculate pairwise genetic distance and construct population phylogeny from VCF files. |
| Article Type: | Technical Note |
| Funding Information: | National Natural Science Foundation of China (82171425) — Dr Nana Jin<br>Scientific Research Foundation for High-Level Talents of the Second Affiliated Hospital of Nantong University (YJRCJJ001) — Dr Nana Jin<br>Scientific Research Foundation for High-Level Talents of the Second Affiliated Hospital of Nantong University (YJRCJJ004) — Dr Lian Xu<br>Shuangchuang Doctor program of Jiangsu Province (JSSCBS20211127) — Dr Lian Xu<br>Hainan Seed Industry Laboratory (JBGS-B23YQ2001) — Dr Xiaodong Fang<br>Hainan Seed Industry Laboratory (JBGS-B23YQ201P) — Dr Xiaodong Fang<br>Project of Sanya Yazhou Bay Science and Technology City (SKJC-2023-02-002) — Dr Xiaodong Fang |


| | |
|---|---|
| Abstract: | Background: Genetic distance metrics are crucial for understanding the evolutionary relationships and population structure of organisms. The advance of next-generation sequencing technology has given rise of genotyping data of thousands of individuals. The standard Variant Call Format (VCF) is widely used to store genomic variation information, but calculating genetic distances and constructing population phylogeny directly from large VCF files can be challenging. Moreover, the existing tools that implement such function remains limited and have low performance, especially in the area of memory efficiency.<br>Findings: To address these issues, we introduce VCF2Dis, an ultra-fast and efficient tool for calculating pairwise genetic distance and constructing population phylogeny directly from large VCF files. Benchmarking results demonstrate the tool's efficiency, with rapid processing times, minimal memory usage, and high accuracy, even when handling datasets with millions of variants from thousands of individuals. Its straightforward command-line interface, compatibility with downstream phylogenetic analysis tools (such as MEGA, Phylip, and FastTree), and support for multithreading make it a valuable tool for researchers studying population relationships. These advantages meaning VCF2Dis has already been widely utilized in many published genomic studies.<br>Conclusion: We present VCF2Dis, a simple tool for calculating genetic distance and constructing population phylogeny directly from large genotype data. This tool has been widely applied and its application can aid exploration population relationship from the large-scale genotype data |



| | |
|---|---|
| Corresponding Author: | Lian Xu, PhD<br>the Second Affiliated Hospital of Nantong University, Nantong University<br>Nantong, CHINA |
| Corresponding Author Secondary Information: | |
| Corresponding Author's Institution: | the Second Affiliated Hospital of Nantong University, Nantong University |
| Corresponding Author's Secondary | |


Powered by Editorial Manager® and ProduXion Manager® from Aries Systems Corporation

| | |
|---|---|
| Institution: | |
| First Author: | Lian Xu, PhD |
| First Author Secondary Information: | |
| Order of Authors: | Lian Xu, PhD |
| | Weiming He |
| | Shuaishuai Tai |
| | Xiaoli Huang |
| | Mumu Qin |
| | Xun Liao |
| | Yi Jing |
| | Jian Yang |
| | Xiaodong Fang |
| | Jianhua Shi |
| | Nana Jin |
| Order of Authors Secondary Information: | |
| Additional Information: | |
| Question | Response |
| Are you submitting this manuscript to a special series or article collection? | No |
| **Experimental design and statistics**<br><br>Full details of the experimental design and statistical methods used should be given in the Methods section, as detailed in our Minimum Standards Reporting Checklist. Information essential to interpreting the data presented should be made available in the figure legends.<br><br>Have you included all the information requested in your manuscript? | Yes |
| **Resources**<br><br>A description of all resources used, including antibodies, cell lines, animals and software tools, with enough information to allow them to be uniquely identified, should be included in the Methods section. Authors are strongly encouraged to cite Research Resource Identifiers (RRIDs) for antibodies, model organisms and tools, where possible. | Yes |

Powered by Editorial Manager® and ProduXion Manager® from Aries Systems Corporation

| | |
|---|---|
| Have you included the information requested as detailed in our Minimum Standards Reporting Checklist? | |
| **Availability of data and materials**<br><br>All datasets and code on which the conclusions of the paper rely must be either included in your submission or deposited in publicly available repositories (where available and ethically appropriate), referencing such data using a unique identifier in the references and in the "Availability of Data and Materials" section of your manuscript.<br><br>Have you have met the above requirement as detailed in our Minimum Standards Reporting Checklist? | Yes |
| GigaScience has policies and guidelines in place for the use of generative AI-writing tools such as ChatGPT. If you have used such writing tools to assist with writing the manuscript this must be declared and cited in the text. Authors should not list AI-writing tools and other AI-assisted technologies as an author or co-author and should acknowledge that they are fully responsible for text generated or refined by AI-writing tools.<br><br> A summary of use (particularly in the introduction or among methods) needs to be included at the end of the paper, and the outputs should also be included as a supplementary file hosted in GigaDB or other open repositories. Please <a href=https://academic.oup.com/gigascience/pages/editorial_policies_and_reporting_standards target="_new" > read our guidelines for more information. </a> <br><br>By submitting to GigaScience, you are aware of the journal's AI-writing tools policy, and if you have declared use of such tools below, you have acknowledged this where appropriate in your manuscript and have made a summary of use and outputs available. </b><br><br> <b>AI-assisted writing tools have been used in the preparation of this manuscript?<br></b><br> | |

Manuscript Click here to access/download;Manuscript;VCF2Dis_manuscript.docx

# VCF2Dis: an ultra-fast and efficient tool to calculate pairwise genetic distance and construct population phylogeny from VCF files

Lian Xu[1,2#], Weiming He[3,4#], Shuaishuai Tai[3], Xiaoli Huang[1], Mumu Qin[4], Xun Liao[3], Yi Jing[4], Jian Yang[2], Xiaodong Fang[3,4], Jianhua Shi[1*], Nana Jin[1,2*]

[1]Institute for translational neuroscience, the Second Affiliated Hospital of Nantong University, Nantong University, Nantong, Jiangsu, 226001, China.

[2]Key Laboratory of Neuroregeneration, Ministry of Education and Jiangsu Province, Co-innovation Center of Neuroregeneration, NMPA Key Laboratory for Research and Evaluation of Tissue Engineering Technology Products, Nantong University, Nantong, Jiangsu, 226001, China.

[3]BGI Research, Shenzhen, 518083, China.

[4]BGI Research, Sanya, 572025, China.

*To whom correspondence: Jianhua Shi (ntshijianhua@ntu.edu.cn) and Nana Jin (yongna0321@126.com).

#These authors contributed equally.

## Abstract

**Background**: Genetic distance metrics are crucial for understanding the evolutionary relationships and population structure of organisms. The advance of next-generation sequencing technology has given rise of genotyping data of thousands of individuals. The standard Variant Call Format (VCF) is widely used to store genomic variation information, but calculating genetic distances and constructing population phylogeny directly from large VCF files can be challenging. Moreover, the existing tools that implement such function remains limited and have low performance, especially in the area of memory efficiency.

**Findings**: To address these issues, we introduce VCF2Dis, an ultra-fast and efficient tool for calculating pairwise genetic distance and constructing population phylogeny directly from large VCF files. Benchmarking results demonstrate the tool's efficiency, with rapid processing times, minimal memory usage, and high accuracy, even when handling datasets with millions of variants from thousands of individuals. Its straightforward command-line interface, compatibility with

downstream phylogenetic analysis tools (such as MEGA, Phylip, and FastTree), and support for multithreading make it a valuable tool for researchers studying population relationships. These advantages meaning VCF2Dis has already been widely utilized in many published genomic studies.

**Conclusion**: We present VCF2Dis, a simple tool for calculating genetic distance and constructing population phylogeny directly from large genotype data. This tool has been widely applied and its application can aid exploration population relationship from the large-scale genotype data.

**Keywords**: VCF2Dis, P-distance, population phylogeny, VCF

## Introduction

With the advance and decreased cost of sequencing technologies, increasing amounts of large-scale genome sequencing of individuals has been performed, such as the 1000 Genomes Project, UK Biobank and 3000 Rice Genomes Project [1-3]. These large-scale genome projects generate a large amount of genetic variation, including single nucleotide polymorphisms (SNPs) and insertions/deletions (indels), and are stored in standard Variant Call Format (VCF). These datasets provide tremendous resource for further exploring genetic diversity. Exploring population structure and relationship are major tasks in evolutionary biology and population genetics. Most current tools for constructing population phylogeny from VCF files firstly convert VCF format into an alignment format (e.g., FASTA and "Phy") and then employ third-party evolutionary phylogenetic software, such as MUSCLE [4], FastME [5], FastTree [6], IQ-TREE [7] and Phylip [8]. These tools include local pipelines or programs, such as SNPhylo [9], VCF-Kit [10], VCFToTree [11], and web-based applications, such as SNiPlay3 [12] and CSI Phylogeny [13]. However, alignment-based methods are computationally demanding and are not well-suited for large-scale genotype datasets due to their high resource consumption, including both computational power and memory.

Currently, two programs, VCF2PopTree [14] and fastreeR [15], are commonly used to calculate genetic distance and then construct population phylogeny directly from VCF files. VCF2PopTree, a JavaScript based client-side application, can calculate p-distance and construct phylogeny using the UPGMA or neighbour-joining algorithms. Although this tool consumes very low running memory, it can only analyze populations with a number of individuals less than 1500. Furthermore, it is slow and easily crashes when the upload file is large. FastreeR, a R package, implements calculating cosine distance and constructs neighbour-joining phylogeny using the Java

programming language. It needs several functions for users to calculate distance, construct phylogeny, and display trees, making it difficult for researchers without advanced programming skills. Furthermore, it is difficult to control memory usage based on Java. Both tools only able to adopt one input file. To address these challenges, we developed a command-line tool, VCF2Dis, a versatile application designed to streamline the calculation of pairwise genetic distance from single or multiple VCF files, construct phylogenetic trees using the UPGMA or the Neighbor-Joining (NJ) method with low memory, and display these trees. Upon its first release, VCF2Dis has undergone continuous refinement, including running time, and has been cited in many high-quality studies, including studies of population relationships in *Rhesus macaque* [16], lablab [17], and watermelon [18].

**Data Description**

To evaluate the performance of VCF2Dis, we used the popular dataset from Phase 3 of the 1000 Genomes Project which sequenced the genomes of 2,504 individuals from 26 populations and characterized over 88 million variants, including 84.7 million SNPs and 3.6 million indels [19].

**Findings**

**Accuracy and performance of VCF2Dis**

VCF2Dis is a simple and straightforward command-line tool that enables users to obtain p-distance matrix and population relationship directly from VCF files (**Fig. 1A**). For the simplest usage, users only need to provide single or multiple input files via the "-InPut" parameter to quickly generate output files, including the p-distance matrix, a Newick format tree and associated figures in PDF and PNG formats. Additionally, users can reconstruct population phylogeny using other alternative phylogenetic software, such as MEGA, Phylip, and FastTree using the p-distance matrix output from VCF2Dis as input. For advanced or customized visualization, annotation, and management of phylogenetic trees, users can upload the Newick format tree to powerful web-based tools, such as iTOL [20] and Evolview [21], or use the ggtree R package [22].

To test its accuracy, we extracted a small dataset from 2,504 human genomes via the parameter, "-SubPop", which contained 203 individuals and 81.2 million variants. The neighbor-joining phylogeny of this dataset revealed three distinct groups, with individuals from the same super

population (YRI: Africa, CEU: European, Asian: CHB and JPT) clustering together (**Fig. 1B**). Notably, individuals from China (CHB) and Japan (JPT) were clearly distinguishable. Since its initial release has been used in studies investigating populations relationships in various organisms, including *Rhesus macaque* [16], lablab [17], and watermelon [18]. These results demonstrate the accuracy and utility of VCF2Dis in population genetic researches.

VCF2Dis is highly efficient in memory usage, processing input files in a line-by-line manner, which makes memory consumption only dependent on the number of individuals (**Fig. 2A** and **2C**). For instance, analyzing 81.2 million variants across 203 individuals required only 0.17 GB of memory. Even when analyzing 2,504 individuals with 81.2 million variants, memory usage only increased to 0.37 GB, demonstrating that a substantial increase in sample size does not significantly impact memory usage. VCF2Dis is also exceptionally fast, completing the analysis of 81.2 million variants across 203 individuals in just about 3 hours. To accelerate the analysis of large-scale genotype data, we also provide a dynamic multiple threading version of VCF2Dis ("VCF2Dis_multi") and recommend it to users for studies when the number of individuals exceeds 1000. For example, when analyzing 6,196,151 variants across 2,504 samples from the 1000 Genomes Project, the single-threaded VCF2Dis took 951 minutes, whereas the "VCF2Dis_multi" completed the task in just 43 minutes using 60 threads, achieving a speedup of approximately 22-fold.

**Comparison with fastreeR**

Although some pipelines, such as VCFToTree, can construct population phylogeny from VCF files, they heavily rely on other third-party programs, such as RAxML [23] and FastME, and require the step of converting VCF input format into a fasta-like format. This step consumes large memory and is also inconvenient for users. Two popular software tools, VCF2PopTree and fastreeR, offer functions for pairwise distance calculation and constructing population phylogeny directly from VCF files (**Table 1**). However, VCF2PopTree, a JavaScript-based local client program, tends to crash when processing datasets with a large number of samples and variants. Therefore, we compared the performance of VCF2Dis and fastreeR in terms of accuracy, runtime, and memory usage. For evaluation, we used a dataset containing 2,065,454 variants across 2,504 individuals. However, fastreeR crashed when handing more than 1000 samples. As a result, we conducted

performance tests on dataset with fewer than 1000 samples. As shown in **Fig. 2A**, fastreeR consumed 42 GB of memory, whereas VCF2Dis used only 3.2MB. Additionally, the memory usage of fastreeR increased significantly as the number of individuals grew, while memory consumption in VCF2Dis remained slightly increased (**Fig. 2A**). We further tested memory usage with varying numbers of variants using a dataset of 10 million variants across 100 individuals. The results indicated an obvious increase in memory usage for fastreeR, while no change in VCF2Dis as the number of variants increased (**Fig. 2C**).

In terms of runtime performance, the tests showed an exponential increase for both fastreeR and VCF2Dis as the number of individuals increased. Although fastreeR was set to use a single thread for comparison, it still occupied multiple threads. Despite this, VCF2Dis consistently outpaced fastreeR, particularly as the sample size increased. For instance, when analyzing 1,000 individuals, fastreeR took 173.64 seconds whereas VCF2Dis completed the task in just 49.84 seconds - approximately 3.48 times faster (**Fig. 2B**). When testing the runtime performance with an increasing number of variants, both fastreeR and VCF2Dis exhibited a linear increase (**Fig. 2D**). However, VCF2Dis was about 12 times faster than fastreeR.

Unlike the p-distance method used in VCF2Dis, fastreeR employs a cosine type distance metric. To compare the accuracy of the two software, we conducted a test using 203 individuals with 3,492 variants from the 1000 Genomes Project which was included as a test dataset used in VCF2PCACluster software [24]. The result showed consistency in the major clusters using these two tools (**Fig. S1**). However, the population phylogeny reconstructed by VCF2Dis appeared to be more accurate, as individuals from the same region consistently clustered together which is the same with the PCA result generated by VCF2PCACluster [24]. In contrast, fastreeR grouped some individuals clustered with population from different regions. For instance, one individual (sample name: NA19007) from the Asian population was clustered with the European population (**Fig. S1**). Overall, these comparisons highlight the accuracy and high performance of VCF2Dis in handing large-scale population genetics analyses.

## Discussion

VCF2Dis is a simple and efficient tool designed to facilitate the calculation of genetic distance and reconstruction of population relationships directly from large VCF files, offering significant advantages for large-scale genomic studies. Since its first release, it has been widely applied and

cited in studies of population relationships. One of the key strengths of VCF2Dis lies in its ability to process VCF files quickly and with extremely low running memory, even for large datasets involving thousands of individuals. This is especially useful given the increasing size of population genomic datasets generated by projects such as the UKB whole-genome sequencing (WGS) consortium and other large-scale sequencing efforts [2, 25]. The integration of multithreading further enhances its performance, providing significant time savings in computationally intensive tasks, as demonstrated by its 22-fold speed improvement over single-threaded execution in our benchmarking tests.

In addition to its efficiency, VCF2Dis offers flexibility. The output files, including p-distance matrices and Newick format trees, can be easily used as inputs for other popular phylogenetic analysis tools like MEGA [26], Phylip, and FastTree, allowing users to build and refine their phylogenetic tree using a variety of software. Moreover, for users who require more advanced visualization and annotation capabilities, the compatibility with tools such as iTOL, Evolview, and the ggtree R package provides extensive options for tree manipulation and display.

However, some limitations should also be considered in the future work. First, VCF2Dis is highly effective for generating p-distance matrices and its utility is dependent on the quality of the input VCF data. In cases where the VCF contains missing or erroneous data, the resulting distance matrix and phylogenetic tree may not accurately reflect the true population structure. Secondly, the current version of VCF2Dis focuses solely on p-distance, which may not be the best metric for all phylogenetic analyses. Incorporating additional genetic distance metrics, such as cosine distance implemented in fastreeR and Hamming distance implemented in PLINK [27], could expand its functionality and improve its applicability to a broader range of evolutionary studies. Thirdly, future developments of VCF2Dis could also address user needs for more interactive features, such as a graphical user interface (GUI), which would lower the entry barrier for non-technical users. Additionally, the integration of support for more advanced evolutionary models could further enhance the versatility of the tool for various phylogenetic and population genetics studies.

In conclusion, VCF2Dis provides a valuable tool for researchers conducting large-scale population genetic studies, offering a fast, flexible, and user-friendly solution for generating p-distance matrices and constructing population phylogenies from VCF files. It enables users to infer population phylogeny directly from VCF files, significantly streamlining the workflow. Despite

some limitations, it remains a powerful option for users seeking to streamline their phylogenetic analysis workflows.

## Methods

### Overview of VCF2Dis workflow

VCF2Dis is implemented with C/C++ and R programming languages, and runs on Linux/Unix and MacOS operating systems. The C/C++ components are mainly used for computational tasks, while R is utilized for generating visualizations (**Fig. 1A**). It can utilize compressed or uncompressed input files with formats of VCF, fasta, and "phy", via "-InPut" and "-InFormat" parameters. Users can provide one or several input files separated by a space or provide a list file with path of input files via "-InList" parameter. Specifically, VCF2Dis can analyze bgzipped/gzip VCF files which allows random access and widely used in big genomic data storage and search. By default, VCF2Dis performs calculation for all samples defined in the input. Recognizing the common need in population genetics to construct phylogenies for specific sub-populations, we provide the '-SubPop' parameter. This feature enables users to easily generate trees for selected sample subsets by specifying them through this parameter. For input of "phy" format, it is firstly converted into fasta format and then calculates p-distance. VCF2Dis employs an external R package, ape [28], to construct population phylogeny and users could choose neighbour-joining or UPGMA algorithms via "-TreeMethod" parameter. To meet the requirement of showing bootstrap values on the branch of phylogeny for some users, we also employed a method of sampling with replacement. For this scenario, users can randomly set a certain ratio (default: 0.25) of all the sites via the parameter, "-Rand", and run VCF2Dis with given repeated times, such as 100 times, to separately construct trees. After that, trees are combined and subject to the fconsense program implemented in the PHYLIPNEW package [29] to construct a consensus tree with bootstrap values. In addition, VCF2Dis employs another R package, ggtree [22], to provide an initial display of population relationship. Users could optionally provide prior group information of individuals for color labelling in the tree figure via "-InSampleGroup" parameter. The outputs of VCF2Dis include p-distance matrix, phylogeny in newick format, and related figures in PDF and PNG formats. With the output of p-distance matrix, users could use other phylogenomic software to reconstruct population phylogeny, such as MEGA [26], FastMe 2.0 [5], Phylip [8], and PHYLIPNEW package

[29]. For advanced and customized visualization of the phylogeny, users can set additional attributes (*e.g.*, layout, color, shape) and modify in our provided custom R script for tree display or use other alternative excellent online tools, such as iTOL [20], Evolview [21] and MEGA [26].

**Pairwise p-distance calculation**

The p-distance is a straightforward approach to estimate genetic distance between two genomes. For genotyping data, the following formula is used to calculate distance ($D_{ij}$) for individual $i$ and $j$ with the total length of $L$ where variants can be identified:

$$D_{ij} = \frac{\sum_{l=1}^{L} d_l}{L}$$

For instance, assuming alleles at the position $l$ are A/C and $d_l$ could be set as followings:
If genotypes of two individuals are the same (AA, CC, or AC) then $d_l = 0$;
If genotypes of two individuals are AA and AC respectively, then $d_l = 0.5$;
If genotypes of two individuals are AA and CC respectively, then $d_l = 1$.
Only bi-allelic variants are considered by most genetic distance calculation tools, such as Vcf2popTree and PLINK. However, multiallelic variants are frequent in population and ignoration could lead to loss of effective genetic information. Thus, we didn't perform any preprocessing of VCF files and compared their genotypes. We adopt a site-by-site of pairwise distance calculation and summed them into a total dissimilarity of the whole genome, namely pairwise distance matrix, which is subjected to phylogenetic software for population phylogeny construction. Furthermore, VCF2Dis also considers genotype data from phased genomes. In phased genomes:
if genotypes of two individuals are AC and AC respectively, then $d_l = 0$;
if genotypes of two individuals are CA and AC respectively, then $d_l = 1$;

**Accelerated method of VCF2Dis**

Large-scale genome sequencing projects generates millions of variants across hundreds of accessions, leading to an extensive memory usage and long running time. For instance, the popular tool PLINK [27] (v1.9) can require more than 257 GB of memory when analyzing a large dataset containing 78 million biallelic SNPs across 2500 human genomes, which is challenging to run on a standard computer. To address this issue, VCF2Dis processes an input file line-by-line, enabling it

to handle large dataset within minimal memory usage (e.g., less than 0.1 GB for analyzing 2500 individuals). To accelerate computation, we have optimized data processing, such as store address pointer instead of the string and reduce the frequency of "substr" and new assignment operations. In addition, we also implemented a multiple thread version of VCF2Dis ("VCF2Dis_multi") via OpenMP library [30].

**Evaluation of performance in running memory and consuming time of existed tools**

To our current knowledge, only three tools have implemented functions of phylogeny construction directly from VCF files, VCF2Dis, VCF2PopTree, and fastreeR. To test the performance of these tools in terms of memory usage and running time for constructing phylogeny from VCF files, we used a large dataset containing 78 million biallelic SNPs across 2500 human genomes. Because VCF2PopTree is a JavaScript based client application and easily crushed when test a moderate dataset. Thus, we only systematically compared the performance between VCF2PopTree and fastreeR. We ran these tools and recorded the memory usage and time of finished jobs with the increase of the number of samples or variants. All the evaluations were conducted on the node with 64 cores and 512 GB of memory using the qsub job scheduler.

## Availability of Source Code and Requirements

Project name: VCF2Dis
Project homepage1: https://github.com/hewm2008/VCF2Dis
Project homepage2: https://github.com/BGI-shenzhen/VCF2Dis
Operating systems(s): Linux/Unix, MacOS
Programming language: C/C++, R
Other requirements:
License: MIT License
VCF2Dis requires minimal external dependencies, making installation simple. It can generate the p-distance matrix without R or related packages, though the visualization features will not be available in this case.

## Additional Files

**Additional file 1: Fig. S1.** The comparison accuracy of phylogenetic trees generated by VCF2Dis and fastreeR using the same test dataset.

## Abbreviations

VCF: Variant Call Format; VCF2Dis: Variant Call Format to distance; SNP: Single-Nucleotide

Polymorphism; Indel: insertion/deletion; GB: Gigabyte; MB: Megabyte; RAM: Random access memory; NJ: Neighbor-Joining; UPGMA: unweighted pair group method with arithmetic mean.

## Author Contributions

NNJ, JHS and WMH conceived the study. WMH developed the tool and performed the analysis. LX, NNJ and JHS provided suggestion for software improvement. LX wrote the draft manuscript. SST, XLH, MMQ, XL, JY, YJ, and XDF involved in the discussion and contributed to manuscript. All authors read and approved the final manuscript.


## Funding

This work was supported by the National Natural Science Foundation of China (Grant No. 82171425), the Scientific Research Foundation for High-Level Talents of the Second Affiliated Hospital of Nantong University (Grant No. YJRCJJ001 and YJRCJJ004), the Shuangchuang Doctor program of Jiangsu Province (Grant No. JSSCBS20211127), Hainan Seed Industry Laboratory (JBGS-B23YQ2001, JBGS-B23YQ201P) and Project of Sanya Yazhou Bay Science and Technology City, Grant No: (SKJC-2023-02-002).


## Data Availability

The datasets used in this study are freely available from the 1000 Genome Project-Phase 3 dataset (https://ftp.1000genomes.ebi.ac.uk/vol1/ftp/release/20130502/) [3].

## Competing Interests

The authors declare no potential competing interests.

**Table 1. The comparison of VCF2Dis and other distance-based tools**

| Software | Programming* | Input format | | | Multiple input files | Sub-population | Algorithm | | Output | | | Memory |
|---|---|---|---|---|---|---|---|---|---|---|---|---|
| | | VCF | FASTA | Phy | | | Distance | Tree | figure | distance matrix | newick tree | |
| VCF2Dis | C/C++ | √ | √ | √ | √ | √ | p-distance | NJ,UPGMA | √ | √ | √ | low |
| VCF2PopTree | JavaScrript | √ | × | × | × | √ | p-distance | NJ,UPGMA | √ | √ | √ | low |
| fastreeR | Java | √ | √ | × | × | × | cosine distance | NJ | √ | √ | √ | high |

*Major programming languages. Red text indicated the tool developed in this study.

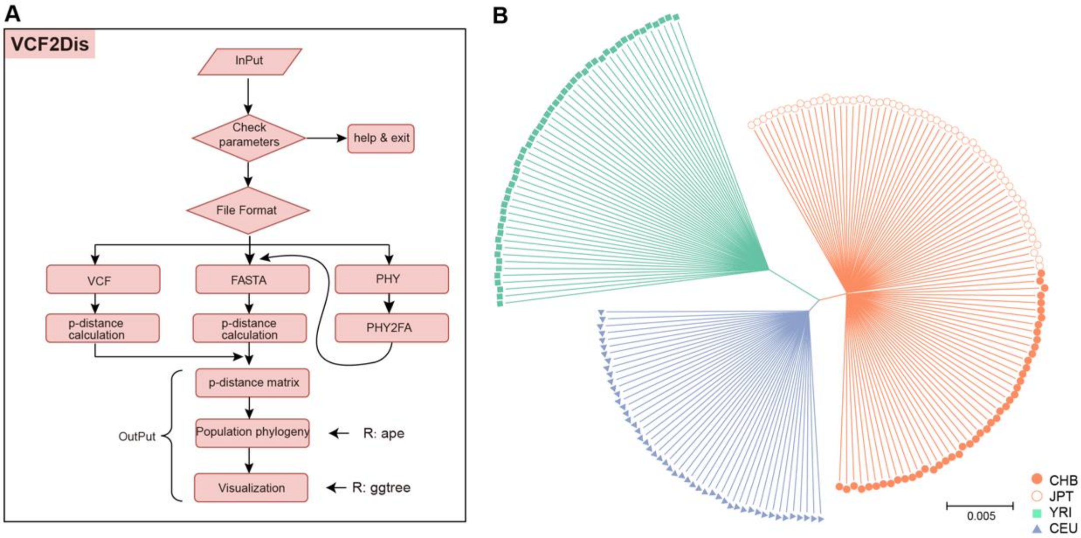


**Figure 1. The workflow of VCF2Dis and neighbor-joining phylogeny generated from a test dataset consisting 203 samples and 80 million bi-allele SNPs isolated from the 1000 human genomes**. **A**, The VCF2Dis workflow involves several key steps, including parameter checks (e.g., input format), p-distance calculation, construction of population phylogeny and phylogeny visualization. VCF2Dis could adopt input with formats of VCF, fasta and “phy”. The outputs include a p-distance matrix, a population phylogeny in newick format and associated figure. **B**, Neighbor-joining phylogeny of 203 individuals. Colors indicated individuals from distinct populations.

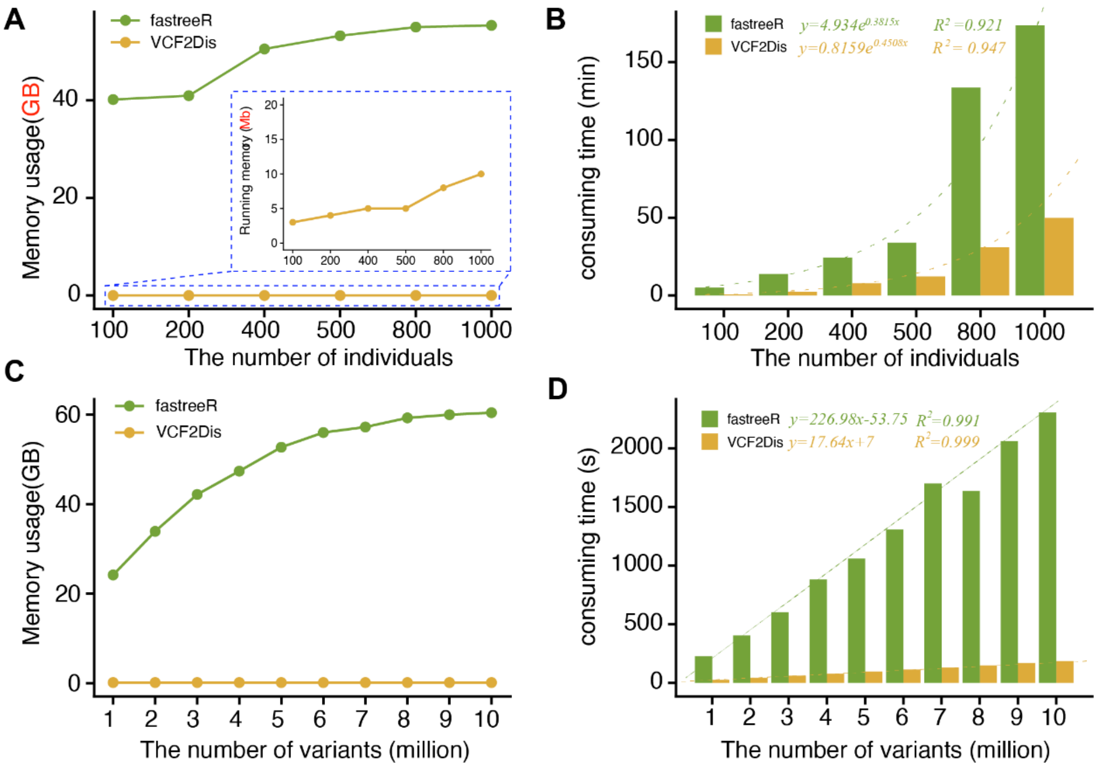


**Figure 2. The performance comparison between VCF2Dis and fastreeR using two test datasets randomly extracted from the 1000 Genome Project**. **A**, The running memory test with the increase of the number of individuals. **B**, Consuming time test in comparison of two tools with the increase of the number of individuals. **C**, The running memory test with the increase of the number of variants. **D**, Consuming time test in comparison of two tools with the increase of the number of variants.

## References


1. Palmer LJ. UK Biobank: bank on it. Lancet. 2007;369 9578:1980-2. doi:10.1016/S0140-6736(07)60924-6.
2. project rg. The 3,000 rice genomes project. Gigascience. 2014;3:7. doi:10.1186/2047-217X-3-7.
3. Siva N. 1000 Genomes project. Nat Biotechnol. 2008;26 3:256. doi:10.1038/nbt0308-256b.
4. Edgar RC. MUSCLE: multiple sequence alignment with high accuracy and high throughput. Nucleic Acids Res. 2004;32 5:1792-7. doi:10.1093/nar/gkh340.
5. Lefort V, Desper R and Gascuel O. FastME 2.0: A Comprehensive, Accurate, and Fast Distance-Based Phylogeny Inference Program. Mol Biol Evol. 2015;32 10:2798-800. doi:10.1093/molbev/msv150.
6. Price MN, Dehal PS and Arkin AP. FastTree 2--approximately maximum-likelihood trees for large alignments. PLoS One. 2010;5 3:e9490. doi:10.1371/journal.pone.0009490.

7. Minh BQ, Schmidt HA, Chernomor O, Schrempf D, Woodhams MD, von Haeseler A and Lanfear R. IQ-TREE 2: New Models and Efficient Methods for Phylogenetic Inference in the Genomic Era. Mol Biol Evol. 2020;37 5:1530-4. doi:10.1093/molbev/msaa015.
8. Felsenstein J. PHYLIP (phylogeny inference package), version 3.5 c. Joseph Felsenstein.; 1993.
9. Lee TH, Guo H, Wang X, Kim C and Paterson AH. SNPhylo: a pipeline to construct a phylogenetic tree from huge SNP data. BMC Genomics. 2014;15:162. doi:10.1186/1471-2164-15-162.
10. Cook DE and Andersen EC. VCF-kit: assorted utilities for the variant call format. Bioinformatics. 2017;33 10:1581-2. doi:10.1093/bioinformatics/btx011.
11. Xu D, Jaber Y, Pavlidis P and Gokcumen O. VCFtoTree: a user-friendly tool to construct locus-specific alignments and phylogenies from thousands of anthropologically relevant genome sequences. BMC Bioinformatics. 2017;18 1:426. doi:10.1186/s12859-017-1844-0.
12. Dereeper A, Homa F, Andres G, Sempere G, Sarah G, Hueber Y, et al. SNiPlay3: a web-based application for exploration and large scale analyses of genomic variations. Nucleic Acids Res. 2015;43 W1:W295-300. doi:10.1093/nar/gkv351.
13. Kaas RS, Leekitcharoenphon P, Aarestrup FM and Lund O. Solving the problem of comparing whole bacterial genomes across different sequencing platforms. PLoS One. 2014;9 8:e104984. doi:10.1371/journal.pone.0104984.
14. Subramanian S, Ramasamy U and Chen D. VCF2PopTree: a client-side software to construct population phylogeny from genome-wide SNPs. PeerJ. 2019;7:e8213. doi:10.7717/peerj.8213.
15. Gkanogiannis A. fastreeR: Phylogenetic, Distance and Other Calculations on VCF and Fasta Files. 2024.
16. Ding W, Li X, Zhang J, Ji M, Zhang M, Zhong X, et al. Adaptive functions of structural variants in human brain development. Sci Adv. 2024;10 14:eadl4600. doi:10.1126/sciadv.adl4600.
17. Njaci I, Waweru B, Kamal N, Muktar MS, Fisher D, Gundlach H, et al. Chromosome-level genome assembly and population genomic resource to accelerate orphan crop lablab breeding. Nat Commun. 2023;14 1:1915. doi:10.1038/s41467-023-37489-7.
18. Zhang Y, Zhao M, Tan J, Huang M, Chu X, Li Y, et al. Telomere-to-telomere Citrullus super-pangenome provides direction for watermelon breeding. Nat Genet. 2024;56 8:1750-61. doi:10.1038/s41588-024-01823-6.
19. Genomes Project C, Auton A, Brooks LD, Durbin RM, Garrison EP, Kang HM, et al. A global reference for human genetic variation. Nature. 2015;526 7571:68-74. doi:10.1038/nature15393.
20. Letunic I and Bork P. Interactive Tree of Life (iTOL) v6: recent updates to the phylogenetic tree display and annotation tool. Nucleic Acids Res. 2024;52 W1:W78-W82. doi:10.1093/nar/gkae268.
21. Subramanian B, Gao S, Lercher MJ, Hu S and Chen WH. Evolview v3: a webserver for visualization, annotation, and management of phylogenetic trees. Nucleic Acids Res. 2019;47 W1:W270-W5. doi:10.1093/nar/gkz357.
22. Xu S, Li L, Luo X, Chen M, Tang W, Zhan L, et al. Ggtree: A serialized data object for visualization of a phylogenetic tree and annotation data. Imeta. 2022;1 4:e56. doi:10.1002/imt2.56.
23. Stamatakis A. RAxML version 8: a tool for phylogenetic analysis and post-analysis of large phylogenies. Bioinformatics. 2014;30 9:1312-3. doi:10.1093/bioinformatics/btu033.
24. He W, Xu L, Wang J, Yue Z, Jing Y, Tai S, et al. VCF2PCACluster: a simple, fast and memory-

efficient tool for principal component analysis of tens of millions of SNPs. BMC Bioinformatics. 2024;25 1:173. doi:10.1186/s12859-024-05770-1.

25. Halldorsson BV, Eggertsson HP, Moore KHS, Hauswedell H, Eiriksson O, Ulfarsson MO, et al. The sequences of 150,119 genomes in the UK Biobank. Nature. 2022;607 7920:732-40. doi:10.1038/s41586-022-04965-x.

26. Tamura K, Dudley J, Nei M and Kumar S. MEGA4: molecular evolutionary genetics analysis (MEGA) software version 4.0. Molecular biology and evolution. 2007;24 8:1596-9.

27. Chang CC, Chow CC, Tellier LC, Vattikuti S, Purcell SM and Lee JJ. Second-generation PLINK: rising to the challenge of larger and richer datasets. Gigascience. 2015;4:7. doi:10.1186/s13742-015-0047-8.

28. Paradis E and Schliep K. ape 5.0: an environment for modern phylogenetics and evolutionary analyses in R. Bioinformatics. 2019;35 3:526-8. doi:10.1093/bioinformatics/bty633.

29. Rice P, Longden I and Bleasby A. EMBOSS: the European Molecular Biology Open Software Suite. Trends Genet. 2000;16 6:276-7. doi:10.1016/s0168-9525(00)02024-2.

30. Dagum L and Menon R. OpenMP: an industry standard API for shared-memory programming. IEEE computational science and engineering. 1998;5 1:46-55.

Table 1

Click here to access/download;Table;Table1.xlsx

| Software | Programming* | Input format | | | Multiple input files | Sub-population | Algor |
|---|---|---|---|---|---|---|---|
| | | VCF | FASTA | Phy | | | Distance |
| VCF2Dis | C/C++ | √ | √ | √ | √ | √ | p-distance |
| VCF2PopTree | JavaScrript | √ | × | × | × | √ | p-distance |
| fastreeR | Java | √ | √ | × | × | × | cosine distance |

| ithm | Output | | | Memory |
|---|---|---|---|---|
| Tree | figure | distance matrix | newick tree | |
| NJ,UPGMA | √ | √ | √ | low |
| NJ,UPGMA | √ | √ | √ | low |
| NJ | √ | √ | √ | high |

Figure 1

Click here to access/download;Figure;Fig1.pdf

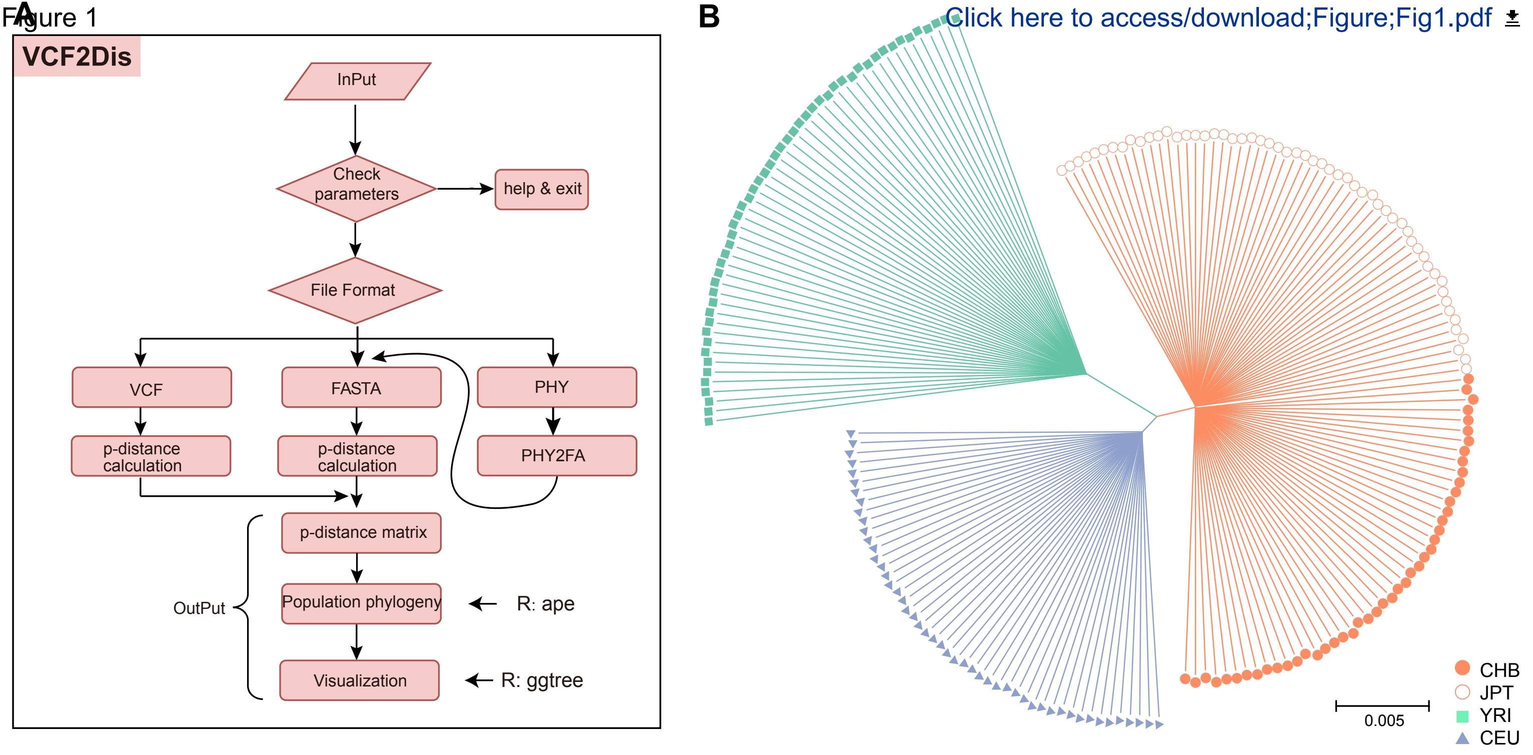

Figure 2

Click here to access/download;Figure;Fig2.pdf

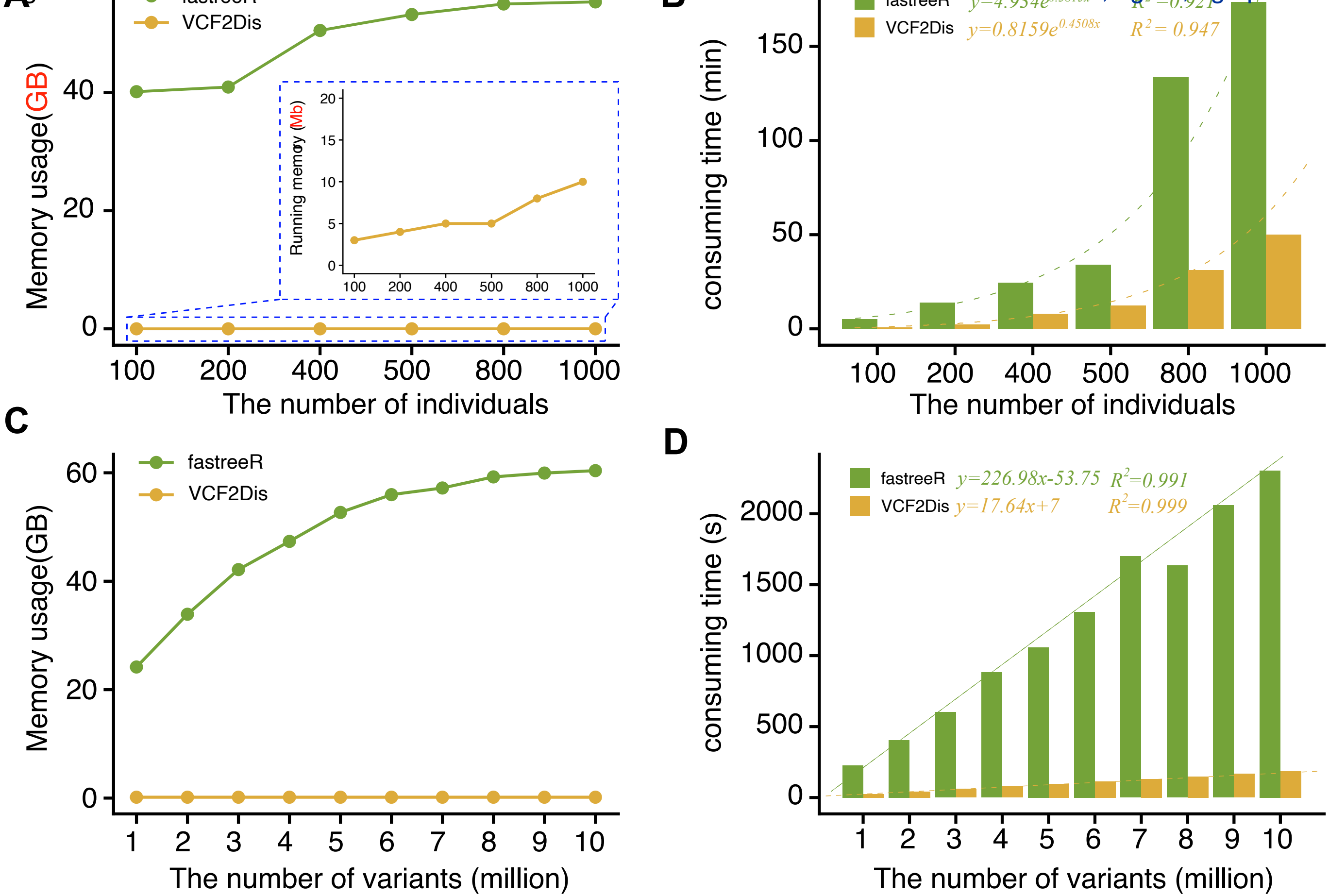

Additional file 1: Fig. S1

Click here to access/download
**Supplementary Material**
Additional_file1.docx
