# Supplementary material for: VCF2Dis: an ultra-fast and efficient tool to calculate pairwise genetic distance and construct population phylogeny from VCF files
Source: Gigascience. 2025 Apr 4;14:giaf032. doi: 10.1093/gigascience/giaf032 (PMC11970368; doi:10.1093/gigascience/giaf032)
Supplement: giaf032_GIGA-D-24-00393_Revision_1 [file giaf032_giga-d-24-00393_revision_1.pdf]

GigaScience

VCF2Dis: an ultra-fast and efficient tool to calculate pairwise genetic distance and construct population phylogeny from VCF files.

--Manuscript Draft--

**Manuscript Number:**	GIGA-D-24-00393R1
**Full Title:**	VCF2Dis: an ultra-fast and efficient tool to calculate pairwise genetic distance and construct population phylogeny from VCF files.
**Article Type:**	Research
**Funding Information:**	National Natural Science Foundation of China (82171425) — Dr Nana Jin<br>Scientific Research Foundation for High-Level Talents of the Second Affiliated Hospital of Nantong University (YJRCJJ001) — Dr Nana Jin<br>Scientific Research Foundation for High-Level Talents of the Second Affiliated Hospital of Nantong University (YJRCJJ004) — Dr Lian Xu<br>Shuangchuang Doctor program of Jiangsu Province (JSSCBS20211127) — Dr Lian Xu<br>Hainan Seed Industry Laboratory (JBGS-B23YQ2001) — Dr Xiaodong Fang<br>Hainan Seed Industry Laboratory (JBGS-B23YQ201P) — Dr Xiaodong Fang<br>Project of Sanya Yazhou Bay Science and Technology City (SKJC-2023-02-002) — Dr Xiaodong Fang
**Abstract:**	Background: Genetic distance metrics are crucial for understanding the evolutionary relationships and population structure of organisms. The advance of next-generation sequencing technology has given rise of genotyping data of thousands of individuals. The standard Variant Call Format (VCF) is widely used to store genomic variation information, but calculating genetic distance and constructing population phylogeny directly from large VCF files can be challenging. Moreover, the existing tools that implement such function remains limited and have low performance in processing large-scale genotype data, especially in the area of memory efficiency.<br>Findings: To address these challenges, we introduce VCF2Dis, an ultra-fast and efficient tool that calculates pairwise genetic distance directly from large VCF files and then constructs distance-based population phylogeny using the ape package. Benchmarking results demonstrate the tool's efficiency, with rapid processing times, minimal memory usage (e.g., 0.37 GB for the whole analysis of 2,504 samples with 81.2 million variants), and high accuracy, even when handling datasets with millions of variants from thousands of individuals. Its straightforward command-line interface, compatibility with downstream phylogenetic analysis tools (such as MEGA, Phylip, and FastTree), and support for multithreading make it a valuable tool for researchers studying population relationships. These advantages meaning VCF2Dis has already been widely utilized in many published genomic studies.<br>Conclusion: We present VCF2Dis, a straightforward and efficient tool for calculating genetic distance and constructing population phylogeny directly from large-scale genotype data. VCF2Dis has been widely applied, facilitating the exploration of population relationship in extensive genome sequencing studies.
**Corresponding Author:**	Lian Xu, PhD<br>the Second Affiliated Hospital of Nantong University, Nantong University<br>Nantong, CHINA
**Corresponding Author Secondary Information:**	
**Corresponding Author's Institution:**	the Second Affiliated Hospital of Nantong University, Nantong University

Powered by Editorial Manager® and ProduXion Manager® from Aries Systems Corporation

**Corresponding Author's Secondary Institution:**	
**First Author:**	Lian Xu, PhD
**First Author Secondary Information:**	
**Order of Authors:**	Lian Xu, PhD
	Weiming He
	Shuaishuai Tai
	Xiaoli Huang
	Mumu Qin
	Xun Liao
	Yi Jing
	Jian Yang
	Xiaodong Fang
	Jianhua Shi
	Nana Jin
**Order of Authors Secondary Information:**	
**Response to Reviewers:**	10 Jan, 2025<br>To<br>The Editor,<br>GigaScience<br><br>Dear Editor,<br>Thank you for carefully reviewing our manuscript, "VCF2Dis: an ultra-fast and efficient tool to calculate pairwise genetic distance and construct population phylogeny from VCF files" (GIGA-D-24-00393).<br><br>First, we appreciate for giving an opportunity to submit our revised manuscript, and also your kindly positive comments and critical suggestions, as well as three reviewers' comments, which have helped us to greatly improved our submitted manuscript as well as our software, VCF2Dis.<br><br>We have carefully read the referees' comments. We would like to express our sincere thanks to the reviewers for their constructive and positive comments. We have addressed all their suggestions, and the manuscript has been edited accordingly, with changes highlighted in red text. Given the lost highlights and supplemented figures in the response text box, we have also uploaded a Word file (coverLetter_Response) containing the cover letter and response as a supplemental material.<br><br>The major amendments were listed below:<br>1)The readily executable version of VCF2Dis has been packaged in popular Docker and Singularity containers, which are available on GitHub (https://github.com/hewm2008/VCF2Dis). Users can easily pull the images and directly run VCF2Dis without the need for compilation and installation. Detailed instructions are provided in the GitHub repository.<br>2)Regarding the inclusion of VCF2PopTree for comparison, we conducted tests on a windows 10 computer (256GB memory, 24 threads, i9 10920x CPU) using a small dataset consisting of 91 samples with 1M, 2M and 3M variants. The source code of VCF2PopTree was slightly modified to include a function for displaying the runtime of the distance calculation step. The runtime for the 1M and 2M variants were 456 s and 596 s respectively. However, VCF2PopTree failed to respond when analyzing the 3M variants, even after 24 hours (a screenshot has been shown in the figure listed below, the figure was shown in the coverLetter_Response file which was uploaded as a attached supplementary material). Similar tests on a Mac M1 max computer yielded a runtime of 111 seconds for the 1M dataset, while the software failed to process the 2M and 3M variants. A similar issue has also been reported by a VCF2PopTree user

Powered by Editorial Manager® and ProduXion Manager® from Aries Systems Corporation

(https://github.com/sansubs/vcf2pop/issues/1). Additionally, the VCF2PopTree developer reported that the software took 3.57 min (214 seconds) to analyze 100 genomes and 2 M variants (PMID: 31824783). Even so, VCF2Dis is approximately at least 10 times faster than VCF2PopTree. Due to these limitations, including the instability, slower performance and failure in running the tests of VCF2PopTree, we decided not to include it in the direct performance comparison. Instead, we discussed its advantages and disadvantages in the revised manuscript, noting its user-friendliness for non-technical users to analyze a small dataset.
Both #reviewer 1 and #reviewer 2 suggested that the efficiency of VCF2Dis might be attributed to the use of the C++ language. As pointed by #reviewer1, we should focus on the performance of distance calculation step because VCF2Dis aims to calculate p-distance. We agreed that the advantage of C++ language is one aspect. We found another C++ tool, ngsDist (https://github.com/fgvieira/ngsDist; https://doi.org/10.1111/bij.12511), which calculate only p-distance. Our tests showed that ngsDist consumes significantly more memory and runs much slower than VCF2Dis (updated Figure 2 in the revised manuscript). This highlights that the efficiency of VCF2Dis is not solely due to the programming language but also its optimized implementation. Additional details have been provided in the Methods section and additional file 1 of the revised manuscript.
3)Considering the importance of reconstructing population phylogeny from large-scale genotype data, the superior performance of VCF2Dis compared to existing genetic distance tools (e.g., VCF2PopTree, fastreeR, ngsDist), and its extensive application in scientific research (with over 190 citations according to Google Scholar, e.g., Nature (wheat, 2024, PMID: 38885696), Nature Genetics (watermelon, 2024, PMID: 38977857), Science Advance (honey bee, 2023, PMID: 37134176; human, 2024, PMID: PMID: 38579006)) underscores its value in the field. We have revised the manuscript to better emphasize the advantages of VCF2Dis in this context.

We hope that with the amendments made in response to the reviewers' comments, the manuscript is now acceptable for publication in GigaScience.

Figure R1. the snapshots of running VCF2PopTree across a dataset of 91 samples with 1 million, 2 million and 3 million variants on a Windows 10 computer. (Note: the figure was shown in the coverLetter_Response file which was uploaded as an attached supplementary material)

Response (blue texts) to comments (black italic) point by point:

Reviewer #1:
comment: In this manuscript, the authors present a new tool, VCF2Dis, which facilitates the construction of phylogenetic trees from large datasets typically presented in VCF format. VCF2Dis provides a fast implementation for calculating pairwise distances and employs the ape tool to construct phylogenetic trees using either the neighbour-joining or UPGMA algorithms. It features an easy-to-use interface that does not require programming skills from the user. The authors compare the performance of VCF2Dis with a tool called fastreeR and demonstrate its superiority in terms of memory efficiency and speed.
Overall, while I find the concept of the manuscript interesting, I believe the introduction lacks sufficient motivation and background, and the comparison with other tools could be more detailed and accurate. Furthermore, the comparison is made against fastreeR, which is not published and does not have a preprint. The novelty of VCF2Dis appears to lie in its efficient implementation of distance evaluation, as it relies on the external tool ape for tree construction. I think this aspect is not adequately emphasized in the manuscript.
I have three major comments and several minor comments that I believe will help improve the manuscript.

Response: Thank you for your professional comments and suggestions, which have been invaluable in improving both our manuscript and the tool. We have thoroughly revised the manuscript in accordance with your professional and constructive suggestions. Below, we have provided detailed responses to each of your major and minor comments.

Powered by Editorial Manager® and ProduXion Manager® from Aries Systems Corporation

Major comments:
comment: 1)Lack of detailed introduction to existing phylogenetic tools. The manuscript does not provide sufficient background on the broader class of phylogenetic methods. VCF2Dis focuses on distance-based methods for phylogenetic tree construction, such as neighbour-joining and UPGMA, which are computationally efficient and use evaluated pairwise distances between samples to build trees. These methods are well-suited for working with large datasets, especially those provided in VCF format, and do not require sequence alignment.
In contrast, other groups of phylogenetic tools, such as RAxML, IQ-TREE, PhyML, and FastTree, use maximum likelihood estimation. These tools rely on substitution models to infer phylogenies, and they require alignment data as input. This class of methods is more complex and provides more accurate evolutionary inferences, but is generally more computationally intensive. They can handle large sample counts, but are restricted by gene-level analyses.
I believe it is essential for the manuscript to provide a broader context and highlight the distinctions between distance-based methods, like those implemented in VCF2Dis, and maximum likelihood methods, as they serve different purposes in phylogenetic analysis. It would also be helpful to explain why many tools use sequence-aligned data instead of VCF format—whether due to traditional practices, methodological requirements, or other reasons.

Response: Thank you for your thoughtful suggestions and kind comments. We apologize for any confusion caused by the unclear statements in the original manuscript. In response to your feedback, we have revised the introduction section to include a more detailed explanation of distance-based methods and maximum likelihood methods, highlighting their distinctions and respective applications in phylogenetic analysis. (lines 52-65)

comment: 2) Comparison with other tools. The manuscript compares VCF2Dis only with fastreeR, an unpublished tool, which makes it difficult to fully assess its strengths and weaknesses. I would expect the authors to compare VCF2Dis with other popular tools, especially VCF2PopTree, which they mention can handle up to 1,500 samples. While the authors mention that VCF2PopTree crashes on large datasets, I believe it should still be included in their comparative analysis to determine the extent to which it can be used effectively.
Due to VCF2Dis's feature for fast distance evaluation, I believe the comparison should focus solely on the distance conversion aspect (see my next comment). I suggest comparing it with PLINK's Hamming distance conversion, though I believe other methods also exist. However, this comparison must be conducted carefully—ideally by comparing conversions to the same distance types or to those with equivalent computational complexity.

Response: Thank you for your thoughtful suggestions and kind comments. FastreeR and VCF2PopTree are current other two tools that generated distance and construct population phylogeny directly from VCF files. FastreeR is indeed an unpublished tool, but it has been applied over 10 studies (https://scholar.google.com/scholar?q=fastreeR, e.g., PMID: 39215560, Mol Ecol, 2024; PMID: 39069530, Commun Biol,2024; PMID: 39443340, Planta,2024; PMID: 39461976, Sci Rep, 2024). Given its usage in the literature, we believe it is reasonable to compare VCF2Dis with FastreeR.

Regarding VCF2PopTree, although its original code limits the number of samples to less than 1500 (We also tested VCF2PopTree and found that it successfully processed a dataset containing 1,500 samples with 500 variants but failed when attempting to process a dataset with 1,501 samples and 500 variants), the performance tests presented in original publication were conducted on the test datasets with fewer than 100 samples and less than 4M variants (https://pmc.ncbi.nlm.nih.gov/articles/PMC6901002/). We conducted tests on a windows 10 computer (256GB memory, 24 threads, i9 10920x CPU) using a small dataset consisting of 91 samples with 1M, 2M and 3M variants. The source code of VCF2PopTree was slightly modified to include a function for displaying the runtime of the distance calculation step. The runtime for the 1M and 2M variants were 456 s and 596 s respectively. However, VCF2PopTree failed to respond when analyzing the 3M

variants, even after 24 hours (a screenshot has been shown in the figure listed below, the figure was shown in the coverLetter_Response file which was uploaded as a attached supplementary material). Similar tests on a Mac M1 max computer yielded a runtime of 111 seconds for the 1M dataset, while the software failed to process the 2M and 3M variants. A similar issue has also been reported by a VCF2PopTree user (https://github.com/sansubs/vcf2pop/issues/1). Additionally, the VCF2PopTree developer reported that the software took 3.57 min (214 seconds) to analyze 100 genomes and 2 M variants (PMID: 31824783). Even so, VCF2Dis is approximately 10 times faster than VCF2PopTree. Due to these limitations, including the instability, slower performance and failure in running the tests of VCF2PopTree, we decided not to include it in the direct performance comparison. Instead, we discussed its advantages and disadvantages in the revised manuscript, noting its user-friendliness for non-technical users to analyze a small dataset. (lines 259-263)

We agreed with your suggestion to focus on distance comparison, as this is a key feature of VCF2Dis. As noted in the original paper of VCF2PopTree, other tools, PLINK and ngsDist can also calculate pairwise genetic distance. PLINK is capable of calculating Hamming distances but requires VCF files to be converted into its own format, which demands substantial memory resources (e.g., 257 GB for analyzing 78M variants across 2,504 human genomes). Additionally, PLINK employs dynamic multi-threading, utilizing most available threads, making direct performance comparisons with other tools less fair. The ngsDist (https://github.com/fgvieira/ngsDist; https://doi.org/10.1111/bij.12511), developed using C++ lanuage, on the other hand, cannot directly handle VCF files and requires external tools (e.g., bcftools → PLINK) to convert the file formats (VCF → BEAGLE). We have included ngsDist in our comparison, and as you suggested, we have updated the performance comparison to focus specifically on the distance calculation step. Our results show that VCF2Dis outperforms the other tools in this regard (updated Figure2 in the manuscript). We have revised the manuscript accordingly, with changes in red text. (e.g., lines 170-200)

Figure R1. the snapshots of running VCF2PopTree across a dataset of 91 samples with 1 million, 2 million and 3 million variants on a Windows 10 computer. (Note: the figure was shown in the coverLetter_Response file which was uploaded as an attached supplementary material)

Figure R2. The snapshot of a similar issue reported in the VCF2PopTree github. (https://github.com/sansubs/vcf2pop/issues/1)(Note: the figure was shown in the coverLetter_Response file which was uploaded as an attached supplementary material)

comment: 3) Comparison procedure. VCF2Dis consists of two components: distance evaluation and tree construction. The first part is novel, with the authors introducing an efficient implementation. The second part relies on the ape software for tree construction. The comparison presented in the manuscript evaluates VCF2Dis and fastreeR in terms of memory usage and speed. Unfortunately, due to the lack of a publication for fastreeR, I was unable to determine the method of tree construction used in that tool. If fastreeR also employs a method from ape, then the comparison is valid. However, if it does not, I believe the manuscript would benefit from conducting two separate comparisons: one for the distance evaluation methods and another for the tree construction methods. It is possible that the difference in efficiency may not stem from the distance evaluation that is a key feature for VCF2Dis, but rather from the fast tree construction in ape.

Response: Thank you for your thoughtful suggestions and kind comments. We have outputted the distance result generated by fastreeR and constructed the population phylogeny using ape package. The tree result is the consistent with the tree generated by VCF2Dis (ape). We also checked the distance method (cosine type distance) defined by the fastreeR author: (1-cosine_similarity)/2 where cosine_similarity could be one of -1(completely opposite samples), 0 (perpendicular samples), and 1 (identifical samples) and it only consider Biallelic or multiallelic (maximum 7 alternate alleles) SNP and/or INDEL variants (README in https://github.com/gkanogiannis/fastreeR). Therefore, for the Biallelic SNPs/INDELs, the “cosine type distance” is the same with p-

Powered by Editorial Manager® and ProduXion Manager® from Aries Systems Corporation

distance and several branch discordances in population phylogeny generated by fastreeR and VCF2Dis is indeed led by different tree reconstructed software. We have corrected and clarified in the revised manuscript. (lines 202-204, 214-218)

Minor Comments:
comment: 1)The ape software used by VCF2Dis for phylogenetic analysis is mentioned at the end of the manuscript in the Methods section. I believe it should be introduced earlier, as its late mention may confuse readers and give the impression that VCF2Dis has its own implementation of phylogenetic tree construction.

Response: Thank you for your thoughtful suggestions and comments. As mentioned in the abstract and introduction, the population phylogeny step, based on the p-distance generated by VCF2Dis, utilizes the ape package. We appreciate your attention to this detail and thank you again for pointing it out. (e.g., lines 30,98, 114)

comment: 2) It would be helpful to provide a definition or at least a brief description of p-distance at the beginning of the manuscript.

Response: Thank you for your kind suggestions and comments. We have added a definition for the p-distance at the beginning of the manuscript in the revised version. (lines 91-92)
The p-distance refers to the proportion (p) of nucleotide sites at which two sequences differ. This metric is well-defined and thoroughly explained in the widely used MEGA software (https://www.megasoftware.net/mega1_manual/Distance.html).

comment: 3) It is unclear how the standard evaluation of p-distance is implemented in VCF2Dis. The manuscript would benefit from a figure illustrating the performance or a pseudocode listing of the implementation. I find the section "Accelerated Method of VCF2Dis" to be very brief.

Response: Thank you for your valuable feedback. We understand the need for further clarity regarding the standard evaluation of p-distance in VCF2Dis. The p-distance is well-defined and thoroughly explained in the widely used MEGA software (https://www.megasoftware.net/mega1_manual/Distance.html). We have added a description and citation in the appropriate location. (e.g., lines 92, 309)
We have provided a more detailed description of the implementation of p-distance in the revised manuscript. (lines 308-328).

Regarding the section “Accelerated Method of VCF2Dis,” we agree that it could benefit from more detailed explanation. In the revised manuscript, we have expanded this section to better describe the optimizations and techniques used to accelerate the computation, providing readers with a clearer understanding of the improvements made. (lines 334-348).

In addition, we have provided a pseudo-code to illustrate the implementation of p-distance calculation and to highlight the improvements in performance (running time and memory usage, Supplementary Note 1 in Additional file 1).

We appreciate your constructive suggestions, which will help improve the manuscript’s clarity and completeness.

comment: 4) To demonstrate that the proposed implementation is both correct and efficient, it would be beneficial to compare VCF2Dis with a naive implementation of p-distance evaluation, implemented in C++.

Response: Thank you for your thoughtful comments. To demonstrate the accuracy of VCF2Dis, it has been widely used in various research studies, with over 190 citations (as of Google Scholar search on December 20, 2024), including publications in high-impact journals such as Nature (PMID: 38885696), Nature Genetic (PMID: 38977857), Nature Communications (PMID: 37069152) and Science Advances (PMID: 38579006). These citations suggest the tool’s robustness and accuracy.

Furthermore, we performed a detailed comparison of the distance results generated by VCF2Dis, fastreeR using a small dataset containing biallelic SNPs without missing

Powered by Editorial Manager® and ProduXion Manager® from Aries Systems Corporation

sites (Figure R3 listed below). The results were identical, although the distance metric defined by fastreeR (cosine type distance) differs. This confirms the correctness and consistency of VCF2Dis.

Regarding performance, we agreed that the advantage of C++ language is one aspect. VCF2Dis was originally developed in C++ and continues to be optimized. Significant improvements were made in versions 1.36, 1.44, and 1.53, with runtime reductions from 4 hours (version 1.36) to 1 hour (version 1.44), and further down to 50 minutes (version 1.53) for the same test dataset. Additionally, we found another C++ tool, ngsDist (https://github.com/fgvieira/ngsDist; https://doi.org/10.1111/bij.12511), which calculate only p-distance. Our tests showed that ngsDist consumes significantly more memory and runs much slower than VCF2Dis (updated Figure 2 in the revised manuscript). This highlights that the efficiency of VCF2Dis is not solely due to the programming language but also its optimized implementation. Additional details have been provided in the Methods section of the revised manuscript and Supplementary Note 1 in Additional file 1. (lines 329-348)

Figure R3. The snapshot includes the runtime performance of fastreeR and VCF2Dis, alongside their corresponding distance matrices, highlighting the computational differences and distance consistency between the two tools. (Note: the figure was shown in the coverLetter_Response file which was uploaded as an attached supplementary material)

comment: 4) I suggest including some final results in the abstract and introduction. These could be part of the results, but they should be specific and impressive. For example, "VCF2Dis requires only 0.37 GB of memory when analyzing 2,504 individuals with 81.2 million variants," or "VCF2Dis performs the analysis of 81.2 million variants across 203 individuals in just about 3 hours and is N times faster than fastreeR."

Response: Thank you for your professional comments. We have added this result in the abstract and introduction. (lines 32-33, 93-96)

comment: 5) The authors introduced a multithreaded version of VCF2Dis and demonstrated that it is 22 times faster when using 60 threads. However, it is unclear how the tool scales with the number of threads. The manuscript indicates that the speed-up is not linear; the speed is only 22 times faster, not 60 times. I suggest the authors provide benchmarking results for VCF2Dis to illustrate its performance with different thread counts.

Response: Thank you for your professional comments. We are sorry for the confusion caused by our unclear statement.

“It seldom occurs that get an N times speedup when running a program parallelized using OpenMP on a N processor platform”. The performance of OpenMP is limited by factors including:
1)When a dependency exists, a process must wait until the data it depends on it computed;
2)When multiple processes share a non-parallel proof resource (like a file to write in), therefore, each thread must wait until the other thread releases the resource;
3)A large part of the program may not be parallelized by OpenMP, which means that the theoretical upper limit of speedup is limited according to Amdahl’s law.
4)Many other common problems affecting the final speedup in parallel computing also apply to OpenMP, like load balancing and synchronization overhead.
Above was copied from the wiki (https://en.wikipedia.org/wiki/OpenMP)

For multi-threaded VCF2Dis, the VCF input file is divided into multiple blocks, which are processed sequentially and calculated in parallel across available threads (Figure R4 of the response below, the figure was shown in the coverLetter_Response file which was uploaded as a attached supplementary material). The OpenMP library is utilized to accelerate the loop calculations within each block (as shown on line 583 in Figure R5 of the response below, the figure was shown in the coverLetter_Response file which was uploaded as an attached supplementary material).

	The non-linear speedup using the multi-threaded VCF2Dis (VCF2Dis_multi) may be caused by following reasons:<br>First, not all steps (e.g., read and write) are separately parallelized. Second, our test of multi-threaded VCF2Dis was performed on a node computer with 64 threads, not all 60 working threads are running at all times during execution, as the CPU usage is dynamic. Third, although 60 threads are employed, the threads often wait for each other during computation, preventing the CPU utilization from reaching 100%. Additionally, the overhead from thread switching consumes a significant amount of CPU resources.<br><br>In the original version of VCF2Dis_multi, we implemented a dynamic parallel strategy that utilized all available CPU cores. In the updated version, we switched to a static parallel strategy, allowing users to specify the number of threads. As per your suggestion, we have included benchmarking results to demonstrate the performance of VCF2Dis_multi with varying thread counts. These tests were conducted on a dataset containing 2,504 samples with 1 million variants (see Figure R6 below and Fig. S1 in Additional File 1, the figure was shown in the coverLetter_Response file which was uploaded as an attached supplementary material). We have revised the manuscript accordingly. (lines 147-165, 235-241)<br><br>Figure R4. The illustration for multi-threaded VCF2Dis. (Note: the figure was shown in the coverLetter_Response file which was uploaded as an attached supplementary material)<br><br>Figure R5. The source code of VCF2Dis for parallel acceleration. (Note: the figure was shown in the coverLetter_Response file which was uploaded as an attached supplementary material)<br><br>Figure R6. The performance of multi-threaded VCF2Dis (VCF2Dis_multi) with different thread counts. The tests were performed on single-threaded mode VCF2Dis and multi-threaded VCF2Dis (threads=2,4,8,16,32). The left y-axis indicates the runtime and the right y-axis indicate thread efficiency. The fold of speedup was shown in yellow shaded texts. The thread efficiency was calculated as the ratio of speedup to the number of threads. (Note: this figure was also shown in Fig. S1 in additional file 1; the figure was shown in the coverLetter_Response file which was uploaded as an attached supplementary material)<br><br>comment: 6) It is stated that, in terms of runtime performance, VCF2Dis and fastreeR exhibited exponential increases. How was this determined? Does it scale exponentially with the number of samples? Could the authors provide an explanation of that complexity? I would expect the complexity to derive from both the distance evaluation and tree construction processes. The distance complexity is O(n^2 M), where nnn is the number of samples and MMM is the number of variants (which also changes with the number of samples). The tree construction complexity should take O(n^3). Therefore, I expect the overall complexity to be O(n^2 M) +O n^3). Could the authors elaborate on this?<br><br>Response: Thank you for your professional comments and suggestions. We are sorry for the confusion caused by unclear statement. As you pointed correctly, the distance complexity is O(n^2 M) and the tree construction complexity is O(n^3) and the overall complexity is O(n^ M) + O(n^3). However, given the fact that M (the number of variants, 10^6) is often much bigger than N (the number of samples, 10^2~10^3), thus the total runtime is mainly determined by the step of distance calculation. Simply, time1 = O(n^2 M), time2 = O(n^3), and time2/time1 <0.01, namely, the runtime of tree construction only occupy less than 1% of the overall runtime. We have clarification this in the revised manuscript. (lines 350-360)<br>To compare the runtime affected by the number of samples and variants, we tested the runtime by fixing the number of variants and increased the number of samples (e.g., 100, 200, 400, 500, 800,1000), or fixing the number of samples and increased the number of variants (1M, 2M, 3M,…,10M). (the performance was shown in Fig.2 of the main text, the test datasets and scripts have been uploaded the giga FTP). We have clarified in the revised manuscript (lines 362-377).

comment: 7) Line 142: "In contrast, fastreeR grouped some 143 individuals clustered with population from different regions.". If fastreeR employs a different method for building the phylogenetic tree (as noted in Major Comment #3) than ape, then it is not surprising that the resulting tree could differ and potentially be less accurate. However, this observation does not provide any insight into the distance metric used. From the experiments, it is not possible to determine whether the discrepancies arise from the distance metrics (p-distance for VCF2Dis and cosine distance for fastreeR) or from the phylogenetic tree construction method. The authors should be clear and accurate in their conclusions.

Response: Thank you for your kind points and professional comments. We are sorry for the confusions caused by unclear statement and not thoroughly considered. We have carefully checked the intermediate distance output and found it was the same as we calculated.
The cosine type distance defined is calculated (1-cosine_similarity)/2, where cosine_similarity could be one of -1(completely opposite samples), 0 (perpendicular samples), and 1 (identifical samples) and it only consider Biallelic or multiallelic (maximum 7 alternate alleles) SNP and/or INDEL variants (README in https://github.com/gkanogiannis/fastreeR). Therefore, for the Biallelic SNPs/INDELs, the “cosine type distance” is the same with p-distance and several branch discordances in population phylogeny generated by fastreeR and VCF2Dis is indeed led by different tree reconstructed software. We have clarified in the revised manuscript (lines 214-218). Thank you for your professional comments.

Reviewer #2:
comment: the authors claim that they have developed an efficient tool to calculate pairwise genetic distance. Also, the tool after processing the pairwise distance, uses phylogenetic inference algorithms very know to produce the tree.
In my opinion, it is not clear on this work what are the algorithmic details of the authors approach that turns their approach more efficient, since, for instance the authors do not discuss a pseudo-code of their approach. Also, what is the asymptotical complexity of the approach? Did the authors did a formal study about that?

Response: Thank you for your kind points and professional comments. We are sorry for the confusions caused by unclear statement. We agreed with your suggestion that it is required the algorithmic details for improving efficiency of VCF2Dis. We have added the details in the revised manuscript. (lines 334-349)
For memory efficiency, we employed a strategy by reading and processing in a manner of line-by-line, ensuring that memory usage is affected only by the number of samples and remains independent of the number of variants. For runtime efficiency, the distance complexity is O(n^2 M) and the tree construction complexity is O(n^3) and the overall complexity is O(n^ M) + O(n^3). However, given the fact that M (the number of variants, 10^6) is often much bigger than N (the number of samples, 10^2~10^3), thus the total runtime is mainly determined by the step of distance calculation. Simply, time1 = O(n^2 M), time2 = O(n^3), and time2/time1 <0.01, namely, the runtime of tree construction only occupy less than 1% of the overall runtime. We have added the details in the revised manuscript (lines 350-360). The pseudo-code of VCF2Dis has been illustrated and added in the supplementary materials (Supplemental Note 1 in Additional file 1).

comment: Moreover, it does not seem a fair comparison to the other tools/approaches since they are written in different languages, executing in different execution environments. It should be important to understand the asymptotical complexity of the different approaches and the experimental evaluation should discuss the diferences that result from the execution environment.

Response: Thank you for your kind points and professional comments. We are sorry for the confusions caused by unclear statement.
We agree to some extent that that development languages may lead to performance difference. VCF2Dis was originally developed in C++ and continues to be optimized. Significant improvements were made in versions 1.36, 1.44, and 1.53, with runtime reductions from 4 hours (version 1.36) to 1 hour (version 1.44), and further down to 50 minutes (version 1.53) for the same test dataset. According to the comment and

suggestions from #reviewer1, that we should focus on the comparison performance in the step of distance calculation. We found another C++ tool, ngsDist (https://github.com/fgvieira/ngsDist; https://doi.org/10.1111/bij.12511), which calculate only p-distance. Our tests showed that ngsDist consumes significantly more memory and runs much slower than VCF2Dis (updated Figure 2 in the revised manuscript). This highlights that the efficiency of VCF2Dis is not solely due to the programming language but also its optimized implementation. Additional details have been provided in the Methods section of the revised manuscript. (lines 362-377)

comment: With respect to the repository, and taking into account that the tool should be executed by non computer science experts, the authors should provide an executable that allow to test the tool.

Response: Thank you for your kind comments for the repository, the readily executable version of VCF2Dis has been packaged in Docker and Singularity containers, which are available on GitHub (https://github.com/hewm2008/VCF2Dis). Users can easily pull the images and run VCF2Dis without the need for compilation and installation. Detailed instructions are provided in the GitHub repository. (lines 274-277)

[Reviewer #3](Additional technical advice)

comment: A number of software tools have been developed in the past to perform population genetic and evolutionary analysis using large-scale genomic data in Variant Call Format (VCF). The present study has developed another software program, 'VCF2Dis,' which receives a VCF file, computes pairwise genetic distance and infers population phylogeny using simple methods such as Neighbour-Joining and UPGMA methods. While this program has some small incremental benefits over the other available software, such as VCF2POPTREE and fastreeR, it does have limitations that the other software don't. Therefore, I don't think this manuscript has merits that warrant publication in GigaScience, but it may be suitable for other specialized journals.

Response: Thank you for your comments. We have clearly stated the limitation of VCF2Dis in the Discussion section: "However, some limitations should also be considered in the future work, …, Thirdly, future developments of VCF2Dis could also address user needs for more interactive features, such as a graphical user interface (GUI), which would lower the entry barrier for non-technical users.". (lines 249-259)
We agree that user-friendly tools like VCF2PopTree enable non-computer science experts to perform pairwise distance calculations and generate NJ trees easily. We conducted tests on a windows 10 computer (256GB memory, 24 threads, i9 10920x CPU) using a small dataset consisting of 91 samples with 1M, 2M and 3M variants. The source code of VCF2PopTree was slightly modified to include a function for displaying the runtime of the distance calculation step. The runtime for the 1M and 2M variants were 456 s and 596 s respectively. However, VCF2PopTree failed to respond when analyzing the 3M variants, even after 24 hours (a screenshot has been shown in the figure listed below). Similar tests on a Mac M1 max computer yielded a runtime of 111 seconds for the 1M dataset, while the software failed to process the 2M and 3M variants. A similar issue has also been reported by a VCF2PopTree user (https://github.com/sansubs/vcf2pop/issues/1). Additionally, the VCF2PopTree developer reported that the software took 3.57 min (214 seconds) to analyze 100 genomes and 2 M variants (PMID: 31824783). Even so, VCF2Dis is approximately 10 times faster than VCF2PopTree. We have discussed its advantages and disadvantages in the revised manuscript, noting its user-friendliness for non-technical users to analyze small dataset. (lines 259-263)

According to the suggestion from #reviewer 1, we also took another p-distance calculation tool, ngsDist developed using C++ language, for performance comparison. Compared to other two tools, fastreeR and ngsDist, VCF2Dis demonstrates superior performance in both memory efficiency and running speed (Figure 2 in the revised manuscript). Considering the importance of reconstructing distance-based population phylogeny from large-scale genotype data, the superior performance of VCF2Dis compared to existing p-distance tools (e.g., VCF2PopTree, fastreeR, ngsDist), and its extensive application in scientific research (with over 190 citations according to Google Scholar, e.g., Nature Genetic (PMID: 38977857), Nature Communications (PMID:

37069152) and Science Advances (PMID: 38579006)) underscores its value in the field.

To facilitate further evaluation, we have made all test datasets available on giga FTP server, and we welcome you to verify the results.

comment: The authors highlight the positive features of VCF2Dis, such as faster execution time compared to VCF2POPTREE and fastreeR. They failed to mention the limitations in comparison with the same software. For instance, VCF2POPTREE is a client-side browser program (no need for installation), and hence, it is easy to draw a phylogeny in a couple of clicks. On the other hand, VCF2Dis is a command line-based program, which has to be complied, installed, and run through a terminal that are not user-friendly, particularly for those who have limited computational skills. Hence, I suggest the authors list the limitations of their software as well. This will inform the users to choose the right software tool that serves their needs.

Response: Thank you for your kind comments and suggestions. We acknowledge that VCF2PopTree is indeed a user-friendly tool. In response, we plan to develop a graphical user interface (GUI) for VCF2Dis to make it more accessible and lower the entry barrier for non-technical users.
We have clearly stated the limitation of VCF2Dis in the Discussion section (lines 249-259). To avoid the compiling and installation, we have made popular docker and singularity containers of VCF2Dis deposited in the github (https://github.com/hewm2008/VCF2Dis/releases/tag/v1.53).(lines 274-277)

comment: Lines 57-58 states that VCF2POPTREE can analyze only <1500 genomes. Lines 120-121 say that fastreeR also crashes when the number of genomes (or samples) is >1000. However, the performance of VCF2Dis was compared only with that of fastreeR but not with that of VCF2POPTREE using 1000 genomes. The reason for this was stated as VCF2POPTREE crashes when the number of genomes was large (lines 116-117). This statement was contradictory to that mentioned in lines 57-58, which means that VCF2POPTREE is capable of analysing 1000 genomes. I suggest that the authors compare the performances of both VCF2POPTREE and fastreeR with VCF2Dis using 1000 or less genomes.

Response: Thank you for your comments. We apologize for the unclear statements and any inappropriate descriptions. We have revised the relevant statements in the revised manuscript.
For fastreeR, it was unable to complete the calculations within a reasonable timeframe when processing datasets with more than 1,000 samples.
For VCF2PopTeee, it is not contradictory for the statement. The limitations of 1500 samples were inferred from the source code of VCF2PopTree (e.g., var all=new Array(1500);). We also tested VCF2PopTree and found that it successfully processed a dataset containing 1,500 samples with 500 variants but failed when attempting to process a dataset with 1,501 samples and 500 variants. In the original publication of VCF2PopTree, the performance tests presented were conducted on the test datasets with fewer than 100 samples and less than 4M variants (https://pmc.ncbi.nlm.nih.gov/articles/PMC6901002/). We conducted tests on a windows 10 computer (256GB memory, 24 threads, i9 10920x CPU) using a small dataset consisting of 91 samples with 1M, 2M and 3M variants. The source code of VCF2PopTree was slightly modified to include a function for displaying the runtime of the distance calculation step. The runtime for the 1M and 2M variants were 456 s and 596 s respectively. However, VCF2PopTree failed to respond when analyzing the 3M variants, even after 24 hours (a screenshot has been shown in the figure listed below). Similar tests on a Mac M1 max computer yielded a runtime of 111 seconds for the 1M dataset, while the software failed to process the 2M and 3M variants. A similar issue has also been reported by a VCF2PopTree user (https://github.com/sansubs/vcf2pop/issues/1). Additionally, the VCF2PopTree developer reported that the software took 3.57 min (214 seconds) to analyze 100 genomes and 2 M variants (PMID: 31824783). Even so, VCF2Dis is approximately 10 times faster than VCF2PopTree. Due to these limitations, including the instability, slower performance and failure in running the tests of VCF2PopTree, we decided not to include it in the direct performance comparison. Instead, we discussed its advantages and disadvantages in the revised manuscript, noting its user-friendliness

Powered by Editorial Manager® and ProduXion Manager® from Aries Systems Corporation

	for non-technical users to analyze a small dataset.<br><br>Figure R1. the snapshots of running VCF2PopTree across a dataset of 91 samples with 1 million, 2 million and 3 million variants on a Windows 10 computer. (Note: the figure was shown in the coverLetter_Response file which was uploaded as an attached supplementary material)
Additional Information:	
Question	Response
Are you submitting this manuscript to a special series or article collection?	No
**Experimental design and statistics**<br><br>Full details of the experimental design and statistical methods used should be given in the Methods section, as detailed in our Minimum Standards Reporting Checklist. Information essential to interpreting the data presented should be made available in the figure legends.<br><br>Have you included all the information requested in your manuscript?	Yes
**Resources**<br><br>A description of all resources used, including antibodies, cell lines, animals and software tools, with enough information to allow them to be uniquely identified, should be included in the Methods section. Authors are strongly encouraged to cite Research Resource Identifiers (RRIDs) for antibodies, model organisms and tools, where possible.<br><br>Have you included the information requested as detailed in our Minimum Standards Reporting Checklist?	Yes
**Availability of data and materials**<br><br>All datasets and code on which the conclusions of the paper rely must be either included in your submission or deposited in publicly available repositories (where available and ethically	Yes

appropriate), referencing such data using a unique identifier in the references and in the “Availability of Data and Materials” section of your manuscript.<br><br>Have you have met the above requirement as detailed in our Minimum Standards Reporting Checklist?	
GigaScience has policies and guidelines in place for the use of generative AI-writing tools such as ChatGPT. If you have used such writing tools to assist with writing the manuscript this must be declared and cited in the text. Authors should not list AI-writing tools and other AI-assisted technologies as an author or co-author and should acknowledge that they are fully responsible for text generated or refined by AI-writing tools.<br><br>A summary of use (particularly in the introduction or among methods) needs to be included at the end of the paper, and the outputs should also be included as a supplementary file hosted in GigaDB or other open repositories. Please read our guidelines for more information. <br>By submitting to GigaScience, you are aware of the journal's AI-writing tools policy, and if you have declared use of such tools below, you have acknowledged this where appropriate in your manuscript and have made a summary of use and outputs available. <br><br>**AI-assisted writing tools have been used in the preparation of this manuscript?<br>** 	

Powered by Editorial Manager® and ProduXion Manager® from Aries Systems Corporation

Revised Manuscript Click here to access/download;Manuscript;revised_manuscript_highlight.doc

VCF2Dis: an ultra-fast and efficient tool to calculate pairwise genetic distance and construct population phylogeny from VCF files

Lian Xu[1,2#], Weiming He[3,4#], Shuaishuai Tai[3], Xiaoli Huang[1], Mumu Qin[4], Xun Liao[3], Yi Jing[4], Jian Yang[2], Xiaodong Fang[3,4], Jianhua Shi[1*], Nana Jin[1,2*]

[1]Institute for translational neuroscience of Affiliated Hospital 2 of Nantong University; Center for neural developmental and degenerative research of Nantong University, Nantong, Jiangsu, 226001, China.

[2]Key Laboratory of Neuroregeneration, Ministry of Education and Jiangsu Province, Co-innovation Center of Neuroregeneration, NMPA Key Laboratory for Research and Evaluation of Tissue Engineering Technology Products, Nantong University, Nantong, Jiangsu, 226001, China.

[3]BGI Research, Shenzhen, 518083, China.

[4]BGI Research, Sanya, 572025, China.

*To whom correspondence: Jianhua Shi (ntshijianhua@ntu.edu.cn) and Nana Jin (yongna0321@126.com).

[#]These authors contributed equally.

Abstract

Background: Genetic distance metrics are crucial for understanding the evolutionary relationships and population structure of organisms. The advance of next-generation sequencing technology has given rise of genotyping data of thousands of individuals. The standard Variant Call Format (VCF) is widely used to store genomic variation information, but calculating genetic distance and constructing population phylogeny directly from large VCF files can be challenging. Moreover, the existing tools that implement such function remains limited and have low performance in processing large-scale genotype data, especially in the area of memory efficiency.

Findings: To address these challenges, we introduce VCF2Dis, an ultra-fast and efficient tool that calculates pairwise genetic distance directly from large VCF files and then constructs distance-based population phylogeny using the ape package.

Benchmarking results demonstrate the tool's efficiency, with rapid processing times, minimal memory usage (*e.g.*, 0.37 GB for the whole analysis of 2,504 samples with 81.2 million variants), and high accuracy, even when handling datasets with millions of variants from thousands of individuals. Its straightforward command-line interface, compatibility with downstream phylogenetic analysis tools (such as MEGA, Phylip, and FastTree), and support for multithreading make it a valuable tool for researchers studying population relationships. These advantages meaning VCF2Dis has already been widely utilized in many published genomic studies.

Conclusion: We present VCF2Dis, a straightforward and efficient tool for calculating genetic distance and constructing population phylogeny directly from large-scale genotype data. VCF2Dis has been widely applied, facilitating the exploration of population relationship in extensive genome sequencing studies.

Keywords: VCF2Dis, p-distance, population phylogeny, VCF

Introduction

With the advance and decreased cost of sequencing technologies, increasing amounts of large-scale genome sequencing of individuals has been performed, such as the 1000 Genomes Project, UK Biobank and 3000 Rice Genomes Project [1-3]. These large-scale genome projects generate a large amount of genetic variation, including single nucleotide polymorphisms (SNPs) and insertions/deletions (indels), and are stored in standard Variant Call Format (VCF). These datasets provide tremendous resource for further exploring genetic diversity. Exploring population structure and relationships are fundamental tasks in evolutionary biology and population genetics, requiring robust methods to infer evolutionary history[4]. Among these methods, distance-based approaches for phylogenetic tree construction, such as neighbor-joining and UPGMA, are computationally efficient and utilize evaluated pairwise distances between genomes to construct trees[4-6]. These methods are particularly well-suited for analyzing large datasets, including those in VCF format, as they do not require sequence alignment. In contrast, another category of phylogenetic tools, such as RAxML[7], IQ-TREE[8], PhyML[9], and FastTree[10], employs maximum likelihood estimation. These tools

rely on substitution models to infer phylogenies and require alignment data as input. This class of methods is more complex and provides more accurate evolutionary inferences, but it is generally more computationally intensive. Although capable of handling large sample counts, their applicability is often constrained to gene-level analyses.

Most current tools for constructing population phylogeny from VCF files firstly convert VCF format into an alignment format (e.g., FASTA and "Phy") and then employ third-party evolutionary phylogenetic software, such as MUSCLE [11], FastME [12], FastTree [10], IQ-TREE [8] and Phylip [13]. These tools include local pipelines or programs, such as SNPhylo [14], VCF-Kit [15], VCFToTree [16], and web-based applications, such as SNiPlay3 [17] and CSI Phylogeny [18]. However, alignment-based methods are computationally demanding and are not well-suited for large-scale genotype datasets due to their high resource consumption, including both computational power and memory.

Currently, two programs, VCF2PopTree [19] and fastreeR [20], are commonly used to calculate genetic distance and then construct distance-based population phylogeny directly from VCF files. VCF2PopTree, a JavaScript-based client-side application, calculates p-distance and constructs a distance-based phylogeny using either the UPGMA or Neighbor-Joining algorithms. While this tool requires minimal memory, its scalability is limited, as it can only process populations with fewer than 1,500 individuals (as inferred from its source code). Furthermore, it is slow and becomes unresponsive when handling a large input file. FastreeR, a R package, implements calculating "cosine" distance and constructs neighbor-joining phylogeny using the Java programming language. It needs several functions for users to calculate distance, construct phylogeny, and display trees, making it difficult for researchers without advanced programming skills. Furthermore, it is difficult to control memory usage based on Java. Both tools only able to adopt one input file. Many efficient tools for such distance-based phylogeny reconstruction have been developed [6]. Nevertheless, the distance calculation step remains a major bottleneck, especially when processing large-scale genomic datasets. To address these challenges, we developed VCF2Dis, a

command-line tool designed to efficiently calculate the p-distance (proportion (p) of nucleotide sites at which two sequences differ[21], **Methods**) matric from single or multiple VCF files with minimal memory consumption (*e.g.*, 0.37 GB for the whole analysis of 2,504 samples with 81.2 million variants) and high computational speed (*e.g.*, 3.48 times and 47.78 times faster than fastreeR and ngsDist, respectively, when calculating the genetic distance for 1,000 individuals with 2 million variants). In addition, it could construct a phylogenetic tree using the UPGMA or the Neighbor-Joining (NJ) method by calling the external ape package[22], and display the tree using the ggtree package[23]. Upon its first release, VCF2Dis has undergone continuous refinement, including running time, and has been cited in many high-quality scientific studies, including studies of population relationships in wheat[24], *Rhesus macaque* [25], lablab [26], and watermelon [27].

Data Description

To evaluate the performance of VCF2Dis, we used the popular dataset from phase 3 of the 1000 Genomes Project which sequenced the genomes of 2,504 individuals from 26 populations and characterized over 88 million variants, including 84.7 million SNPs and 3.6 million indels [28].

Findings

Accuracy and performance of VCF2Dis

VCF2Dis is a simple and straightforward command-line tool that enables users to obtain p-distance matrix directly from one or multiple VCF files, and infer distance-based population relationship using the external ape package (**Fig. 1A**). For the simplest usage, users only need to provide single or multiple input files via the "-InPut" parameter to quickly generate output files, including a p-distance matrix, a Newick format tree and associated figures in PDF and PNG formats. Additionally, users can reconstruct population phylogeny using other alternative phylogenetic software, such as MEGA, Phylip, and FastTree using the p-distance matrix output from VCF2Dis as input. For advanced or customized visualization, annotation, and management of

phylogenetic trees, users can upload the Newick format tree to powerful web-based tools, such as iTOL [29] and Evolview [30], or use the ggtree R package [23].

To test its accuracy, we extracted a small dataset from 2,504 human genomes via the parameter, "-SubPop", which contained 203 individuals and 81.2 million variants. The neighbor-joining phylogeny of this dataset revealed three distinct groups, with individuals from the same super population (YRI: Africa, CEU: European, Asian: CHB and JPT) clustering together (**Fig. 1B**). Notably, individuals from China (CHB) and Japan (JPT) were clearly distinguishable. Since its initial release, it has been used in studies investigating population relationships in various organisms, including wheat[24], *Rhesus macaque* [25], lablab [26], and watermelon [27]. These evidences demonstrate the accuracy and utility of VCF2Dis in population genetic researches.

VCF2Dis is highly memory-efficient, as it processes input files in a line-by-line manner. This approach ensures that memory consumption depends solely on the number of individuals, rather than the total size of the dataset, making it particularly suitable for handling large-scale genotype data. For instance, analyzing 81.2 million variants across 203 individuals required only 0.17 GB of memory. Even when analyzing 2,504 individuals with 81.2 million variants, the memory usage only increased to 0.37 GB, demonstrating that a substantial increase in sample size does not significantly impact memory usage.

VCF2Dis is also exceptionally fast. To speedup runtime, we utilize pointer-based string operations to reduce memory allocation and assignment operation during data parsing. Furthermore, we only calculate upper-triangle matrix to reduce the computational workload by eliminating redundant operations (Methods and Supplemental Note 1 in Additional file 1). It completed the analysis of 81.2 million variants across 203 individuals in just about 3 hours. To accelerate the analysis of large-scale genotype data, we also provide a multiple threading version of VCF2Dis (named "VCF2Dis_multi") by paralleling *for* loop using OpenMP library. We tested the performance of VCF2Dis_multi in distance calculation step on different thread counts (n=2,4,8,16,32) with a dataset containing 1 million variants across 2,504 samples from the 1000 Genomes Project. The result showed that the runtime generally decreases as

the number of threads increases, but the reduction is not perfectly linear (**Fig. S1** in Additional file 1). In these tests, the best speedup was 19-fold achieved using 32 threads that VCF2Dis_multi took 8.1 minutes while the single-threaded VCF2Dis took 157.8 minutes (**Fig. S1** in Additional file 1 and **Table S3** in Additional file 2). We also compared and tested the performance of VCF2Dis and VCF2Dis_multi in distance calculation step across the number of variants and samples. The runtime of both single-threaded and multi-threaded VCF2Dis exhibited a linear relationship with the number of variants (**Fig. S2A** in Additional file 1). In this scenario, the multi-threaded version achieved a speedup of 2-3 times compared to the single-threaded implementation. For the tested sample sizes ranging from 100 to 2,500, the runtime of multi-threaded VCF2Dis demonstrated significant improvement, achieving over an 11-fold speedup when the sample size exceeded 600 (**Fig. S2B** in Additional File 1 and **Table S2** in Additional file 2). Therefore, the multi-threaded VCF2Dis is highly suitable for analyzing large-scale genomic datasets, particularly those involving thousands of individuals.

Performance comparison with other existing tools

Two tools, VCF2PopTree and fastreeR offer functions for pairwise distance calculation and constructing population phylogeny directly from VCF files (**Table 1**). However, VCF2PopTree, a JavaScript-based local client program, failed to process datasets with a large number of samples and variants (*e.g.*, 91 samples with 3M variants). We also found another tool, ngsDist[31], developed in C/C++, which is capable of calculating p-distance. However, this tool requires an additional preprocessing step - converting VCF format into PLINK format - to function correctly (**Table 1**). The runtime complexity of VCF2Dis is primarily determined by the distance calculation step (see Methods for details). Additionally, since VCF2Dis focuses on p-distance while tree reconstruction is handled by an external tool, we compared the performance of VCF2Dis, fastreeR and ngsDist in terms of runtime and memory usage during the distance calculation process. We also consider the number of samples and the number of variants on the performance (**Methods**).

In terms of memory usage, VCF2Dis consumed extremely low memory compared to other two tools (**Fig. 2A** and **2C**). For example, VCF2Dis required only 10 MB of memory to analyze 1,000 samples with 2 million variants, whereas fastreeR and ngsDist consumed 55.36 GB and 92.83 GB, respectively (**Table S1** in the Additional file 2). The memory usage of VCF2Dis is independent of the number of variants and increases slightly with the number of samples (**Fig. 2**). In contrast, the memory usage of fastreeR approximately follows a logarithmic increase with the number of variants and samples, whereas ngsDist exhibits a linear relationship with both the number of samples and the number of variants.

In terms of runtime performance, the runtime of all three tools shows a linear increase with the number of variants. In this situation, VCF2Dis demonstrates the fastest performance, being approximately 12 times and 36 times faster than fastreeR and ngsDist, respectively (**Fig. 2B**). Regarding the number of samples, the runtime of all three tools approximately follows a pattern where the time taken is proportional to the square of the sample size. However, VCF2Dis showed the fastest performance, being approximately 3 times and 45 times faster than fastreeR and ngsDist, respectively (**Fig. 2D**). For instance, when analyzing 1,000 individuals, VCF2Dis took 49.84 seconds, while fastreeR took 173.64 seconds and ngsDist took 2,381.68 seconds, which is approximately 3.48 times and 47.78 times faster (**Table S1** in Additional file 2), respectively. Therefore, VCF2Dis consistently outpaced fastreeR and ngsDist, particularly as the sample size increased.

Unlike VCF2Dis, which uses the p-distance method and the external ape package for tree construction, fastreeR employs a “cosine” distance metric and constructs a Neighbor-joining phylogeny using its own built-in implementation. To compare the accuracy of the two software, we conducted a test using 203 individuals with 3,492 variants from the 1000 Genomes Project which was included as a test dataset used in VCF2PCACluster software [32]. The tree result showed consistency in the major clusters using these two tools (**Fig. S3**). However, the population phylogeny reconstructed by VCF2Dis appeared to be more accurate, as individuals from the same region consistently clustered together which is the same with the PCA result generated

by VCF2PCACluster [32]. In contrast, fastreeR grouped some individuals clustered with population from different regions. For instance, one individual (sample name: NA19006) from the Asian population was clustered with the European population (**Fig. S3**). We also compared the distance results and found them to be identical between the tools. Therefore, the inconsistencies observed in the phylogenetic trees generated by VCF2Dis and fastreeR may be attributed to the different methods employed for tree construction. We recommend users of fastreeR to utilize alternative tools (*e.g.*, ape) for constructing population phylogeny after completing the distance calculation. Overall, these comparisons highlight the accuracy and high performance of VCF2Dis in handing large-scale population genetics analyses.

Discussion

VCF2Dis is a simple and efficient tool designed to facilitate the calculation of genetic distance and reconstruction of population relationships directly from large VCF files, offering significant advantages for large-scale genomic studies. Since its first release, it has been widely applied and cited in studies of population relationships, such as wheat[24], *Rhesus macaque* [25], lablab [26], and watermelon [27]. One of the key strengths of VCF2Dis lies in its ability to calculate p-distance quickly with extremely low running memory, even for large datasets involving thousands of individuals. This is especially useful given the increasing size of population genomic datasets generated by projects such as the UKB whole-genome sequencing (WGS) consortium and other large-scale sequencing efforts [2, 33]. The integration of multithreading further enhances its performance, providing significant time savings in computationally intensive tasks, as demonstrated by its 19-fold speed improvement over single-threaded execution in our benchmarking tests of 2,504 samples with 1 million variants using 32 threads. It is important to note that the speedup achieved by “VCF2Dis_multi” is often nonlinear compared to the single-threaded version of VCF2Dis. Factors such as the overhead of thread management, uneven workload distribution among threads, and the fact that not all steps (*e.g.*, I/O) in the process are fully parallelizable can impact parallel efficiency. Consequently, we recommend employing the multi-threaded version of

VCF2Dis for studies involving thousands of individuals, as it provides substantial computational advantages.

In addition to its efficiency, VCF2Dis offers flexibility. The output files, including p-distance matrices and the Newick format tree, can be easily used as inputs for other popular phylogenetic analysis tools like MEGA [21], Phylip, and FastTree, allowing users to build and refine their phylogenetic tree using a variety of software. Moreover, for users who require more advanced visualization and annotation capabilities, the compatibility with tools such as iTOL, Evolview, and the ggtree R package provides extensive options for tree manipulation and display.

However, some limitations should also be considered in the future work. First, VCF2Dis is highly effective for generating p-distance matrices and its utility is dependent on the quality of the input VCF data. In cases where the VCF contains missing or erroneous data, the resulting distance matrix and phylogenetic tree may not accurately reflect the true population structure. Secondly, the current version of VCF2Dis focuses solely on p-distance, which may not be the best metric for all phylogenetic analyses. Future incorporation of additional genetic distance metrics could expand the functionality of VCF2Dis and enhance its applicability to a broader range of evolutionary studies. Thirdly, future developments of VCF2Dis could also address user needs for more interactive features, such as a graphical user interface (GUI), which would lower the entry barrier for non-technical users. Although VCF2PopTree was not included in the performance comparison due to its failure in the most tests, its user-friendly interface, which requires just one click, makes it a viable option for scientific experts without advanced computational skills, particularly for the analysis of small datasets.

In conclusion, VCF2Dis provides a valuable tool for researchers conducting large-scale population genetic studies, offering a fast, flexible, and user-friendly solution for generating p-distance matrices and constructing population phylogenies from VCF files. It enables users to infer distance-based population phylogeny directly from VCF files, significantly streamlining the workflow. Despite some limitations, it remains a powerful option for users seeking to streamline their phylogenetic analysis workflows.

Methods

Overview of VCF2Dis workflow

VCF2Dis is implemented with C/C++ and R programming languages, and runs on Linux/Unix and MacOS operating systems. The C/C++ components are mainly used for computational tasks, while R is utilized for generating visualizations (**Fig. 1A**). We have also provided both Docker and Singularity containerized versions of VCF2Dis, enabling users to bypass the compilation and installation process for a seamless experience. VCF2Dis can utilize compressed or uncompressed input files with formats of VCF, fasta, and "phy", via "-InPut" and "-InFormat" parameters. Users can provide one or several input files separated by a space or provide a list file with path of input files via "-InList" parameter. Specifically, VCF2Dis can analyze bgzipped/gzip VCF files which allows random access and widely used in big genomic data storage and search. By default, VCF2Dis performs calculation for all samples defined in the input. Recognizing the common need in population genetics to construct phylogenies for specific sub-populations, we provide the '-SubPop' parameter. This feature enables users to easily generate trees for selected sample subsets by specifying them through this parameter. For input of "phy" format, it is firstly converted into fasta format and then calculates p-distance. VCF2Dis employs an external R package, ape [22], to construct population phylogeny and users could choose neighbor-joining or UPGMA algorithms via "-TreeMethod" parameter. To meet the requirement of showing bootstrap values on the branch of phylogeny for some users, we also employed a method of sampling with replacement. For this scenario, users can randomly set a certain ratio (default: 0.25) of all the sites via the parameter, "-Rand", and run VCF2Dis with given repeated times, such as 100 times, to separately construct trees. After that, trees are combined and subject to the fconsense program implemented in the PHYLIPNEW package [34] to construct a consensus tree with bootstrap values. In addition, VCF2Dis employs another R package, ggtree [23], to provide an initial display of population relationship. Users could optionally provide prior group information of individuals for color labelling in the tree figure via "-InSampleGroup" parameter. The outputs of VCF2Dis include p-distance matrix, phylogeny in Newick format, and

related figures in PDF and PNG formats. With the output of p-distance matrix, users could use other phylogenomic software to reconstruct population phylogeny, such as MEGA [21], FastMe 2.0 [12], Phylip [13], and PHYLIPNEW package [34]. For advanced and customized visualization of the phylogeny, users can set additional attributes (*e.g.*, layout, color, shape) and modify in our provided custom R script for tree display or use other alternative excellent online or localized interactive tools, such as iTOL [29], Evolview [30] and MEGA [21].

The p-distance calculation

The p-distance is a straightforward approach to estimate genetic distance between two genomes [21]. For genotyping data, the following formula is used to calculate distance (D_{ij}) for individual i and j with the total length of L where variants can be identified:

$$D_{ij} = \frac{\sum_{l=1}^{L} d_l}{L}$$

For instance, assuming alleles at the position l are A/C and d_l could be set as followings:

If genotypes of two individuals are the same (AA, CC, or AC) then $d_l = 0$;

If genotypes of two individuals are AA and AC respectively, then $d_l = 0.5$;

If genotypes of two individuals are AA and CC respectively, then $d_l = 1$.

Only bi-allelic variants are considered by most genetic distance calculation tools, such as Vcf2popTree and PLINK. However, multiallelic variants are frequent in population and ignoration could lead to loss of effective genetic information. Thus, we didn’t perform any preprocessing of VCF files and compared their genotypes. We adopt a site-by-site of pairwise distance calculation and summed them into a total dissimilarity of the whole genome, namely pairwise distance matrix, which is subjected to external phylogenetic software (ape) for population phylogeny construction. Furthermore, VCF2Dis also considers genotype data from phased genomes. In phased genomes:

if genotypes of two individuals are AC and AC respectively, then $d_l = 0$;

if genotypes of two individuals are CA and AC respectively, then $d_l = 1$;

Accelerated methods of VCF2Dis

Large-scale genome sequencing projects generate millions of variants across hundreds of accessions, leading to an extensive memory usage and long runtime. For instance, the popular tool PLINK [35] (v1.9) can require more than 257 GB of memory when analyzing a large dataset containing 78 million biallelic SNPs across 2500 human genomes[32], which is challenging to run on a standard computer. To address memory concerns, VCF2Dis adopts a streaming processing approach, reading and calculating data line-by-line rather than loading the entire VCF file into memory before processing. This method enables efficient handling of large dataset within minimal memory usage (e.g., less than 0.1 GB for analyzing 2500 individuals in distance calculation step). To accelerate its runtime, we have made two major improvements during data processing. First, we utilized pointer-based string operations, reducing overhead associated with memory allocation and assignment operation during data parsing. This results in faster extraction of relevant fields from input files, as compared to traditional string manipulation methods. Furthermore, we have optimized the computation process by employing upper-triangle calculations, which significantly reduce the computational workload by eliminating redundant operations. These optimizations ensure that VCF2Dis is both faster and more memory-efficient. The pseudo-code for these major improvements and details were shown in Supplemental Note 1 in Additional file 1. In addition, we also implemented a multiple thread version of VCF2Dis ("VCF2Dis_multi") by paralleling *for* loop using OpenMP library [36].

The runtime complexity of VCF2Dis

The runtime complexity of VCF2Dis is primarily determined by two main components: p-distance matrix calculation and tree construction. For p-distance matrix calculation, this step has a complexity of $O(n^2 \ m)$, where n represents the number of samples and m represents the number of variants. Each pair of samples requires a comparison across m variants. For tree construction step, the Neighbor-Joining method used for tree construction has a complexity of $O(n^3)$, as it involves iterative clustering of n samples. The overall runtime complexity is therefore $O(n^2 \ m) + O(n^3)$. Given that m (commonly $>10^6$) is typically much larger than n (commonly $<10^3$), the runtime

complexity is predominantly determined by the p-distance matrix calculation step, making it is nearly $O(n^2 \ m)$ of VCF2Dis in practical scenarios.

Evaluation of performance in memory usage and runtime of existing tools

To evaluate performance, we assessed the memory usage and runtime of existing tools, VCF2Dis, fastreeR and ngsDist, which are designed for calculating genetic distance and/or reconstruct distance-based population phylogeny. fastreeR was installed via the Bioconductor package, while ngsDist was downloaded from its GitHub repository (https://github.com/fgvieira/ngsDist). Test datasets were generated from the 1000 Genome Project. To evaluate the number of samples on performance, we used a dataset containing 2 million variants across 2,504 individuals from the 1000 Genome Project. However, fastreeR was unable to complete the calculations within a reasonable timeframe, while ngsDist consumed excessive memory resources and was terminated by the system when processing datasets with more than 1,000 samples. Consequently, we conducted performance tests on datasets with fewer than 1,000 samples (100, 200, 300, …, up to 1000), each containing 2 million variants. To evaluate the effect of the number of variants on performance, datasets were created with fixed 91 samples, containing 1 million, 2 million, 3 million, …, up to 10 million variants each. The tools were executed according to their respective documentation, and the memory usage and runtime of completed jobs were recorded. Results were visualized using the ggplot2 package and have been shown in Additional file 2. All evaluations were performed on a computational node with 64 cores and 512 GB of memory, managed using the qsub job scheduler.

Availability of Source Code and Requirements

Project name: VCF2Dis

Project homepage: https://github.com/hewm2008/VCF2Dis

Operating systems(s): Linux/Unix, MacOS

Programming language: C/C++, R

License: MIT License

VCF2Dis requires minimal external dependencies, making installation simple. It can generate the p-distance matrix without R or related packages, though the visualization features will not be available in this case.

Additional Files

Additional file 1: Figure S1. The performance of multi-threaded VCF2Dis (VCF2Dis_multi) with different thread counts**. Figure S2.** The performance of multi-threaded VCF2Dis (VCF2Dis_multi) with different number of variants and samples compared to the single-threaded VCF2Dis**. Figure S3.** The accuracy comparison of Neighbor-joining based phylogenetic trees generated by VCF2Dis (using the ape package) and fastreeR (using the dist2tree function implemented in fastreeR package) using the same test dataset. Supplementary Note 1: The pseudocode for improving memory and runtime of VCF2Dis.

Additional file 2: Table S1. The performance comparison of the distance calculation step using VCF2Dis, fastreeR, and ngsDist was conducted across varying numbers of variants and individuals. **Table S2.** The performance comparison of the distance calculation step between VCF2Dis_multi and VCF2Dis was conducted across varying sample sizes and numbers of variants. **Table S3.** The performance of the distance calculation step in the multi-threaded version of VCF2Dis (VCF2Dis_multi) was evaluated using different thread counts.

Abbreviations

VCF: Variant Call Format; VCF2Dis: Variant Call Format to distance; SNP: Single-Nucleotide Polymorphism; Indel: insertion/deletion; M: million; GB: Gigabyte; MB: Megabyte; NJ: Neighbor-Joining; UPGMA: unweighted pair group method with arithmetic mean.

Author Contributions

NNJ, JHS and WMH conceived the study. WMH developed the tool and performed the analysis. LX, NNJ and JHS provided suggestion for software improvement. LX wrote the draft manuscript. SST, XLH, MMQ, XL, JY, YJ, and XDF involved in the discussion and contributed to manuscript. All authors read and approved the final manuscript.

Funding

This work was supported by the National Natural Science Foundation of China (Grant No. 82171425), the Scientific Research Foundation for High-Level Talents of the Second Affiliated Hospital of Nantong University (Grant No. YJRCJJ001 and YJRCJJ004), the Shuangchuang Doctor program of Jiangsu Province (Grant No. JSSCBS20211127), Hainan Seed Industry Laboratory (JBGS-B23YQ2001, JBGS-B23YQ201P) and Project of Sanya Yazhou Bay Science and Technology City, Grant No: (SKJC-2023-02-002).

Data Availability

The datasets used in this study are freely available from the 1000 Genome Project-Phase 3 dataset (https://ftp.1000genomes.ebi.ac.uk/vol1/ftp/release/20130502/) [3].

Competing Interests

The authors declare no potential competing interests.

Table 1. The comparison of VCF2Dis and other distance-based tools

Software	Programming*	Input format			Multiple	Sub-	Algorithm		Output			Memory
		VCF	FASTA	Phy	input files	population	Distance	Tree	figure	distance matrix	newick tree	
VCF2Dis	C/C++	√	√	√	√	√	p-distance	NJ,UPGMA	√	√	√	low
VCF2PopTree	JavaScript	√	×	×	×	√	p-distance	NJ,UPGMA	√	√	√	low
fastreeR	Java	√	√	×	×	×	cosine distance#	NJ	√	√	√	high
ngsDist	C/C++	×	×	×	×	×	p-distance	×	×	√	×	high

*Major programming languages. #defined in the fastreeR. Red text indicated the tool developed in this study.

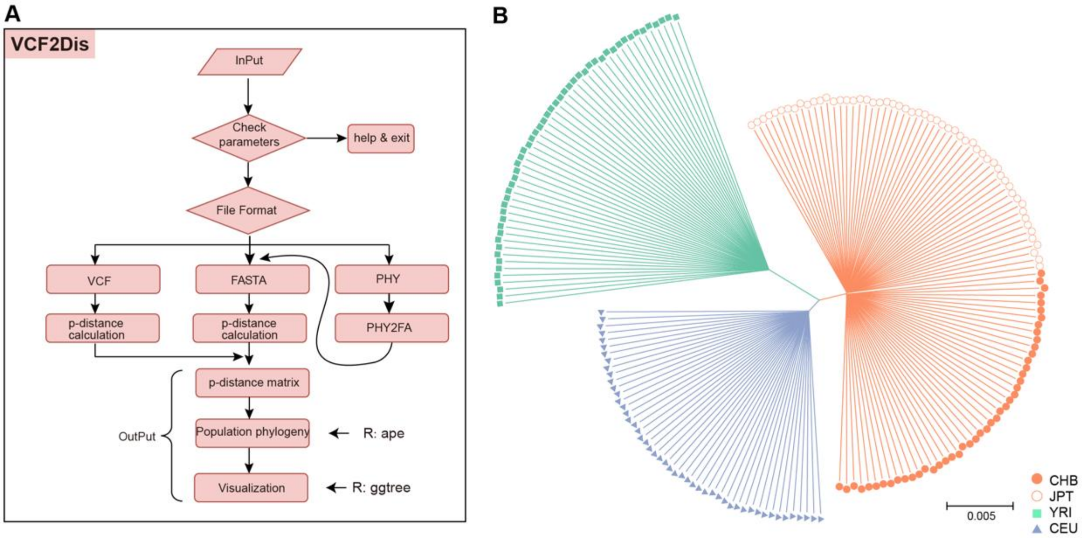

Figure 1. The workflow of VCF2Dis and neighbor-joining phylogeny generated from a test dataset consisting 203 samples and 81.2 million bi-allele SNPs isolated

from the 1000 human genomes. **A**, The VCF2Dis workflow involves several key steps, including parameter checks (*e.g.*, input format), p-distance calculation, construction of population phylogeny and phylogeny visualization. VCF2Dis could adopt input with formats of VCF, fasta and "phy". The outputs include a p-distance matrix, a population phylogeny in newick format and associated figure. **B**, Neighbor-joining phylogeny of 203 individuals. Colors indicated individuals from distinct populations. YRI: Africa; CEU: European; CHB: China; JPT: Japan.

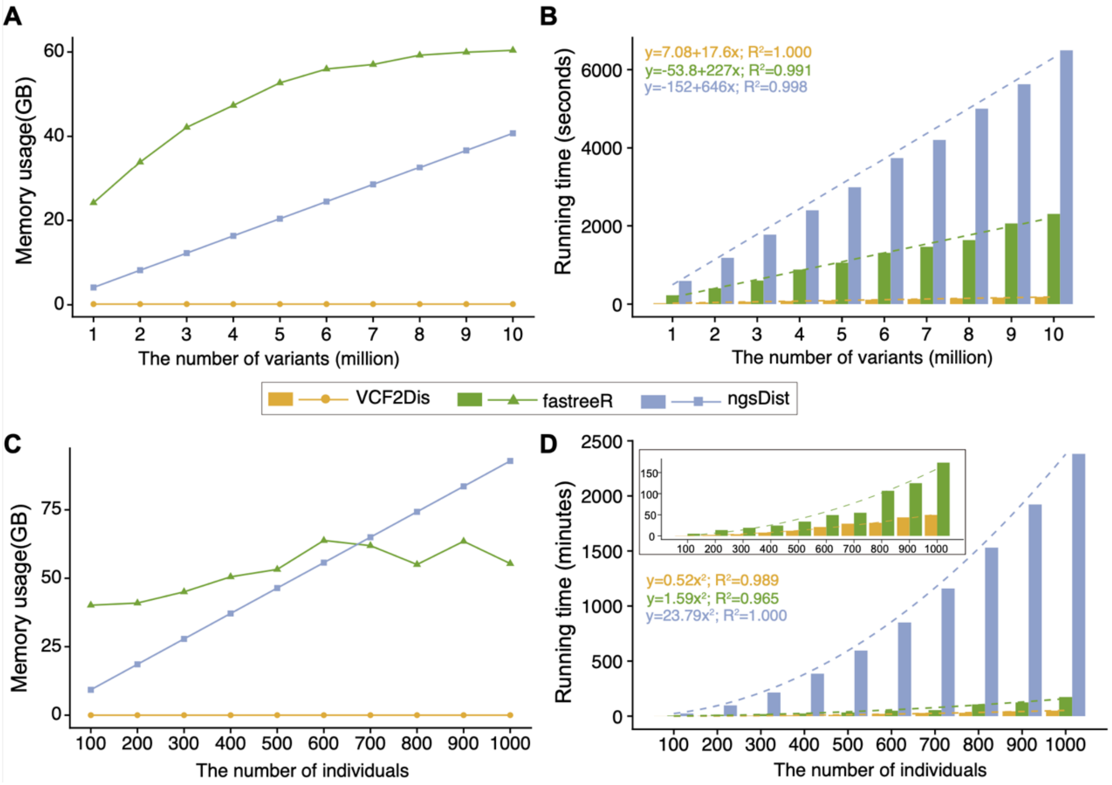

Figure 2. The memory and runtime performance of VCF2Dis, fastreeR, and ngsDist were assessed based on the number of variants and samples in calculating genetic distance. A, The memory test with an increasing number of variants in a dataset containing 91 samples. **B**, The runtime test with an increasing number of variants in a dataset containing 91 samples. **C**, The memory test with an increasing number of individuals, each containing 2 million variants. **D**, The runtime test with an increasing number of individuals, each containing 2 million variants. The runtime of VCF2Dis and fastreeR were also separately shown in the inner box.

References

1. Palmer LJ. UK Biobank: bank on it. Lancet. 2007;369 9578:1980-2. doi:10.1016/S0140-6736(07)60924-6.

2. The 3,000 rice genomes project. The 3,000 rice genomes project. Gigascience. 2014;3:7. doi:10.1186/2047-217X-3-7.
3. Siva N. 1000 Genomes project. Nat Biotechnol. 2008;26 3:256. doi:10.1038/nbt0308-256b.
4. Holder M and Lewis PO. Phylogeny estimation: traditional and Bayesian approaches. Nat Rev Genet. 2003;4 4:275-84. doi:10.1038/nrg1044.
5. Pardi F and Gascuel O. Combinatorics of distance-based tree inference. Proc Natl Acad Sci U S A. 2012;109 41:16443-8. doi:10.1073/pnas.1118368109.
6. Vaz C, Nascimento M, Carrico JA, Rocher T and Francisco AP. Distance-based phylogenetic inference from typing data: a unifying view. Brief Bioinform. 2021;22 3 doi:10.1093/bib/bbaa147.
7. Stamatakis A. RAxML version 8: a tool for phylogenetic analysis and post-analysis of large phylogenies. Bioinformatics. 2014;30 9:1312-3. doi:10.1093/bioinformatics/btu033.
8. Minh BQ, Schmidt HA, Chernomor O, Schrempf D, Woodhams MD, von Haeseler A and Lanfear R. IQ-TREE 2: New Models and Efficient Methods for Phylogenetic Inference in the Genomic Era. Mol Biol Evol. 2020;37 5:1530-4. doi:10.1093/molbev/msaa015.
9. Guindon S, Dufayard JF, Lefort V, Anisimova M, Hordijk W and Gascuel O. New algorithms and methods to estimate maximum-likelihood phylogenies: assessing the performance of PhyML 3.0. Syst Biol. 2010;59 3:307-21. doi:10.1093/sysbio/syq010.
10. Price MN, Dehal PS and Arkin AP. FastTree 2--approximately maximum-likelihood trees for large alignments. PLoS One. 2010;5 3:e9490. doi:10.1371/journal.pone.0009490.
11. Edgar RC. MUSCLE: multiple sequence alignment with high accuracy and high throughput. Nucleic Acids Res. 2004;32 5:1792-7. doi:10.1093/nar/gkh340.
12. Lefort V, Desper R and Gascuel O. FastME 2.0: A Comprehensive, Accurate, and Fast Distance-Based Phylogeny Inference Program. Mol Biol Evol. 2015;32 10:2798-800. doi:10.1093/molbev/msv150.
13. Felsenstein J. PHYLIP (phylogeny inference package), version 3.5 c. Joseph Felsenstein.; 1993.
14. Lee TH, Guo H, Wang X, Kim C and Paterson AH. SNPhylo: a pipeline to construct a phylogenetic tree from huge SNP data. BMC Genomics. 2014;15:162. doi:10.1186/1471-2164-15-162.
15. Cook DE and Andersen EC. VCF-kit: assorted utilities for the variant call format. Bioinformatics. 2017;33 10:1581-2. doi:10.1093/bioinformatics/btx011.
16. Xu D, Jaber Y, Pavlidis P and Gokcumen O. VCFtoTree: a user-friendly tool to construct locus-specific alignments and phylogenies from thousands of anthropologically relevant genome sequences. BMC Bioinformatics. 2017;18 1:426. doi:10.1186/s12859-017-1844-0.
17. Dereeper A, Homa F, Andres G, Sempere G, Sarah G, Hueber Y, et al. SNiPlay3: a web-based application for exploration and large scale analyses of genomic variations. Nucleic Acids Res. 2015;43 W1:W295-300. doi:10.1093/nar/gkv351.
18. Kaas RS, Leekitcharoenphon P, Aarestrup FM and Lund O. Solving the problem of comparing whole bacterial genomes across different sequencing platforms. PLoS One. 2014;9 8:e104984. doi:10.1371/journal.pone.0104984.
19. Subramanian S, Ramasamy U and Chen D. VCF2PopTree: a client-side software to construct population phylogeny from genome-wide SNPs. PeerJ. 2019;7:e8213. doi:10.7717/peerj.8213.
20. Gkanogiannis A. fastreeR: Phylogenetic, Distance and Other Calculations on VCF and Fasta Files. 2024.

21. Tamura K, Dudley J, Nei M and Kumar S. MEGA4: molecular evolutionary genetics analysis (MEGA) software version 4.0. Molecular biology and evolution. 2007;24 8:1596-9.
22. Paradis E and Schliep K. ape 5.0: an environment for modern phylogenetics and evolutionary analyses in R. Bioinformatics. 2019;35 3:526-8. doi:10.1093/bioinformatics/bty633.
23. Xu S, Li L, Luo X, Chen M, Tang W, Zhan L, et al. Ggtree: A serialized data object for visualization of a phylogenetic tree and annotation data. Imeta. 2022;1 4:e56. doi:10.1002/imt2.56.
24. Cheng S, Feng C, Wingen LU, Cheng H, Riche AB, Jiang M, et al. Harnessing landrace diversity empowers wheat breeding. Nature. 2024;632 8026:823-31. doi:10.1038/s41586-024-07682-9.
25. Ding W, Li X, Zhang J, Ji M, Zhang M, Zhong X, et al. Adaptive functions of structural variants in human brain development. Sci Adv. 2024;10 14:eadl4600. doi:10.1126/sciadv.adl4600.
26. Njaci I, Waweru B, Kamal N, Muktar MS, Fisher D, Gundlach H, et al. Chromosome-level genome assembly and population genomic resource to accelerate orphan crop lablab breeding. Nat Commun. 2023;14 1:1915. doi:10.1038/s41467-023-37489-7.
27. Zhang Y, Zhao M, Tan J, Huang M, Chu X, Li Y, et al. Telomere-to-telomere Citrullus super-pangenome provides direction for watermelon breeding. Nat Genet. 2024;56 8:1750-61. doi:10.1038/s41588-024-01823-6.
28. The 1000 Genomes Project Consortium. A global reference for human genetic variation. Nature. 2015;526 7571:68-74. doi:10.1038/nature15393.
29. Letunic I and Bork P. Interactive Tree of Life (iTOL) v6: recent updates to the phylogenetic tree display and annotation tool. Nucleic Acids Res. 2024;52 W1:W78-W82. doi:10.1093/nar/gkae268.
30. Subramanian B, Gao S, Lercher MJ, Hu S and Chen WH. Evolview v3: a webserver for visualization, annotation, and management of phylogenetic trees. Nucleic Acids Res. 2019;47 W1:W270-W5. doi:10.1093/nar/gkz357.
31. Vieira FG, Lassalle F, Korneliussen TS and Fumagalli M. Improving the estimation of genetic distances from Next-Generation Sequencing data. Biological journal of the Linnean Society. 2016;117 1:139-49.
32. He W, Xu L, Wang J, Yue Z, Jing Y, Tai S, et al. VCF2PCACluster: a simple, fast and memory-efficient tool for principal component analysis of tens of millions of SNPs. BMC Bioinformatics. 2024;25 1:173. doi:10.1186/s12859-024-05770-1.
33. Halldorsson BV, Eggertsson HP, Moore KHS, Hauswedell H, Eiriksson O, Ulfarsson MO, et al. The sequences of 150,119 genomes in the UK Biobank. Nature. 2022;607 7920:732-40. doi:10.1038/s41586-022-04965-x.
34. Rice P, Longden I and Bleasby A. EMBOSS: the European Molecular Biology Open Software Suite. Trends Genet. 2000;16 6:276-7. doi:10.1016/s0168-9525(00)02024-2.
35. Chang CC, Chow CC, Tellier LC, Vattikuti S, Purcell SM and Lee JJ. Second-generation PLINK: rising to the challenge of larger and richer datasets. Gigascience. 2015;4:7. doi:10.1186/s13742-015-0047-8.
36. Dagum L and Menon R. OpenMP: an industry standard API for shared-memory programming. IEEE computational science and engineering. 1998;5 1:46-55.

 Click here to access/download;Table;Table1.xlsx

Software	Programming*	Input format			Multiple input files	Sub-population
		VCF	FASTA	Phy		
VCF2Dis	C/C++	√	√	√	√	√
VCF2PopTree	JavaScript	√	×	×	×	√
fastreeR	Java	√	√	×	×	×
ngsDist	C/C++	×	×	×	×	×

*Major programming languages. #defined in the fastreeR. Red text indicated the tool de

Algorithm		Output			Memory
**Distance**	**Tree**	**figure**	**distance matrix**	**newick tree**	
p-distance	NJ,UPGMA	√	√	√	low
p-distance	NJ,UPGMA	√	√	√	low
cosine distance#	NJ	√	√	√	high
p-distance	×	×	√	×	high

eveloped in this study.

Figure 1

Click here to access/download;Figure;Figure1.pdf

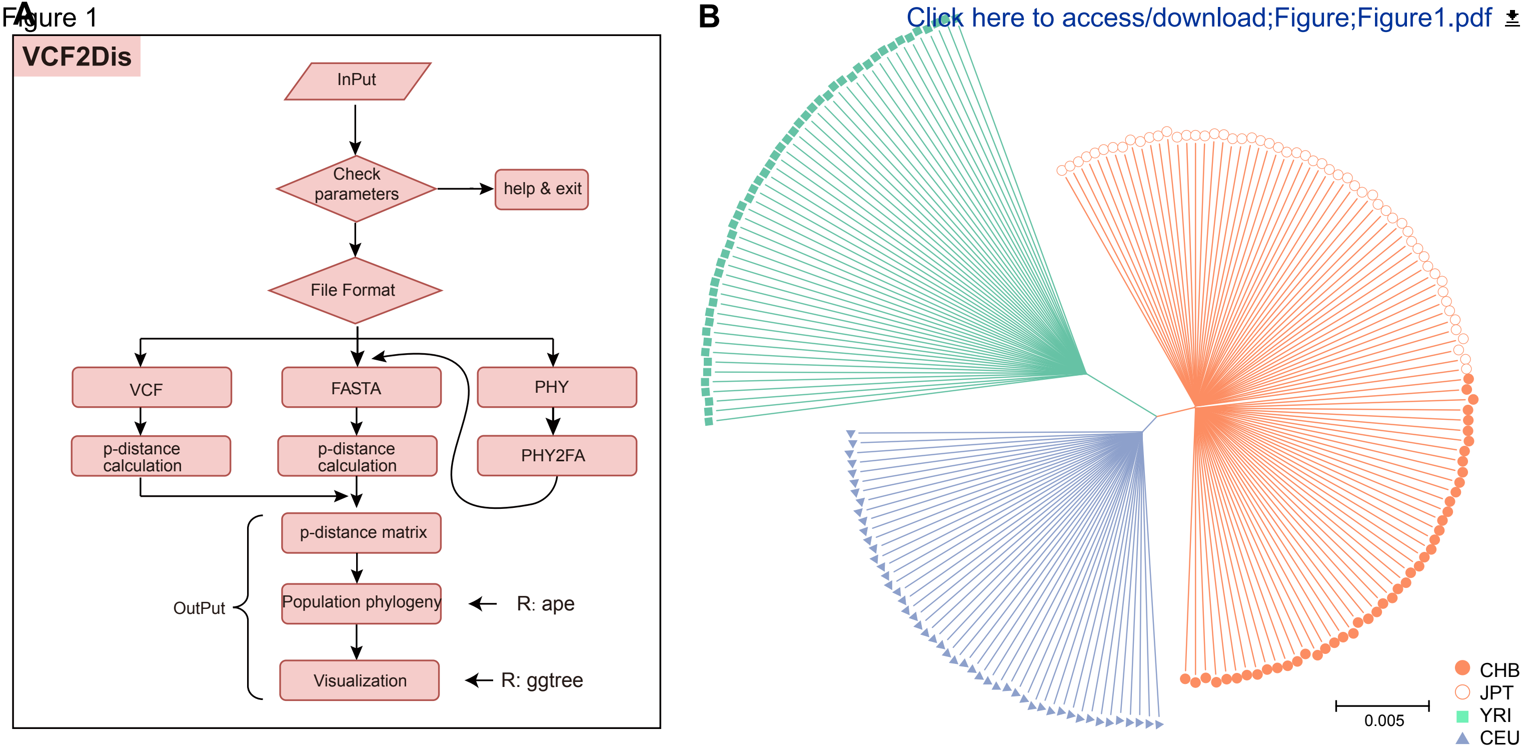

Figure 2

Click here to access/download;Figure;Figure2.pdf

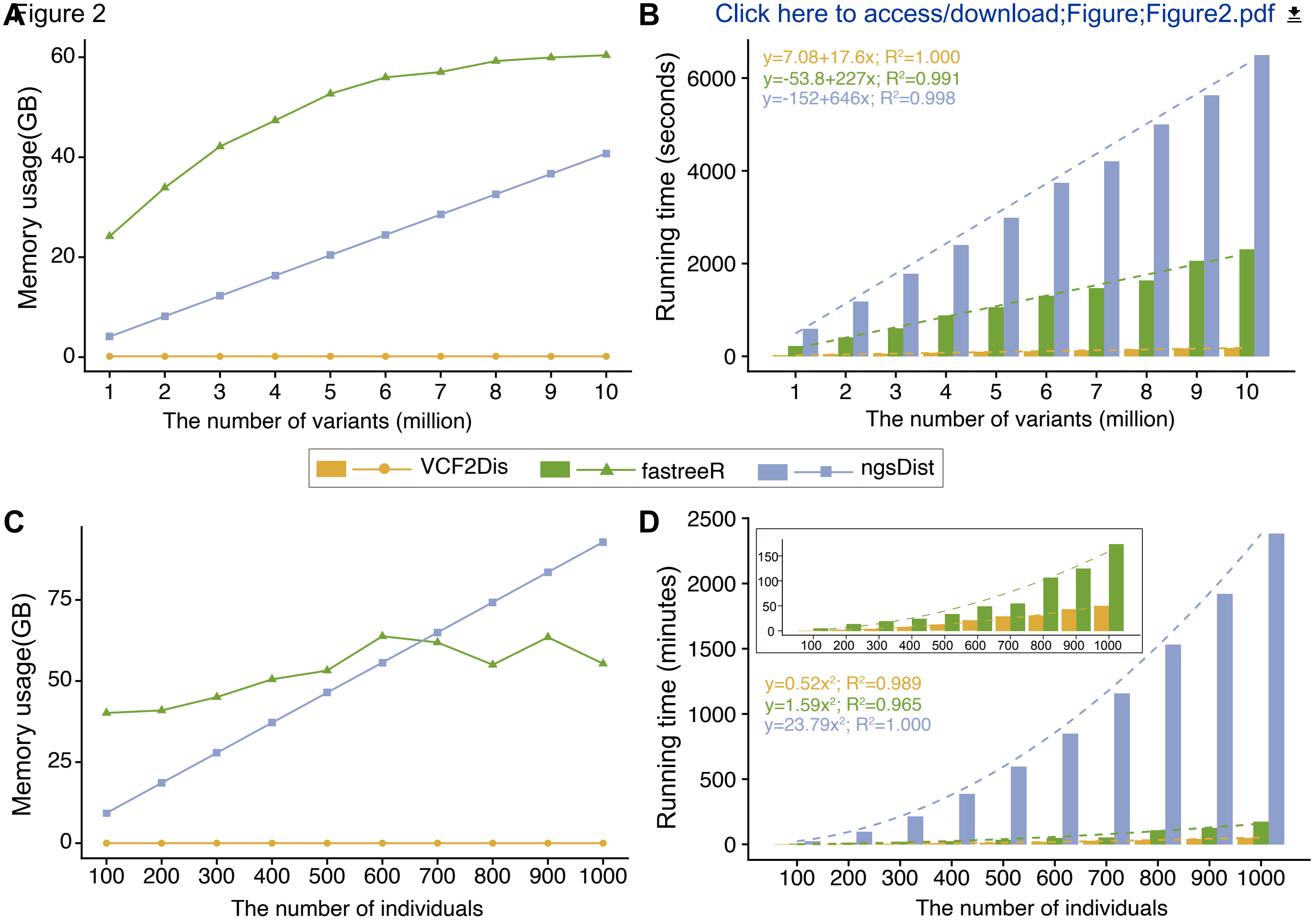

Figs. S1-S3 and Supplemental Note 1

Click here to access/download
Supplementary Material
Addtionally_File1.docx

Tables S1-S3 for performance of running test

Click here to access/download
Supplementary Material
Additional file 2.xlsx

Cover letter and Response with highlights

Click here to access/download

Supplementary Material

coverLetter_Response.docx
