# Supplementary material for: VCF2Dis: an ultra-fast and efficient tool to calculate pairwise genetic distance and construct population phylogeny from VCF files
Source: Gigascience. 2025 Apr 4;14:giaf032. doi: 10.1093/gigascience/giaf032 (PMC11970368; doi:10.1093/gigascience/giaf032)
Supplement: giaf032_GIGA-D-24-00393_Revision_2 [file giaf032_giga-d-24-00393_revision_2.pdf]

GigaScience

VCF2Dis: an ultra-fast and efficient tool to calculate pairwise genetic distance and construct population phylogeny from VCF files

--Manuscript Draft--

**Manuscript Number:**	GIGA-D-24-00393R2
**Full Title:**	VCF2Dis: an ultra-fast and efficient tool to calculate pairwise genetic distance and construct population phylogeny from VCF files
**Article Type:**	Technical Note
**Funding Information:**	National Natural Science Foundation of China (82171425) — Dr Nana Jin<br>Scientific Research Foundation for High-Level Talents of the Second Affiliated Hospital of Nantong University (YJRCJJ001) — Dr Nana Jin<br>Scientific Research Foundation for High-Level Talents of the Second Affiliated Hospital of Nantong University (YJRCJJ004) — Dr Lian Xu<br>Shuangchuang Doctor program of Jiangsu Province (JSSCBS20211127) — Dr Lian Xu<br>Hainan Seed Industry Laboratory (JBGS-B23YQ2001) — Dr Xiaodong Fang<br>Hainan Seed Industry Laboratory (JBGS-B23YQ201P) — Dr Xiaodong Fang<br>Project of Sanya Yazhou Bay Science and Technology City (SKJC-2023-02-002) — Dr Xiaodong Fang
**Abstract:**	Background : Genetic distance metrics are crucial for understanding the evolutionary relationships and population structure of organisms. The advance of next-generation sequencing technology has given rise of genotyping data of thousands of individuals. The standard Variant Call Format (VCF) is widely used to store genomic variation information, but calculating genetic distance and constructing population phylogeny directly from large VCF files can be challenging. Moreover, the existing tools that implement such function remains limited and have low performance in processing large-scale genotype data, especially in the area of memory efficiency.<br>Findings: To address these challenges, we introduce VCF2Dis, an ultra-fast and efficient tool that calculates pairwise genetic distance directly from large VCF files and then constructs distance-based population phylogeny using the ape package. Benchmarking results demonstrate the tool's efficiency, with rapid processing times, minimal memory usage(e.g., 0.37 GB for the whole analysis of 2,504 samples with 81.2 million variants), and high accuracy, even when handling datasets with millions of variants from thousands of individuals. Its straightforward command-line interface, compatibility with downstream phylogenetic analysis tools (such as MEGA, Phylip, and FastTree), and support for multithreading make it a valuable tool for researchers studying population relationships. These advantages meaning VCF2Dis has already been widely utilized in many published genomic studies.<br>Conclusion: We present VCF2Dis, a straightforward and efficient tool for calculating genetic distance and constructing population phylogeny directly from large-scale genotype data. VCF2Dis has been widely applied, facilitating the exploration of population relationship in extensive genome sequencing studies.
**Corresponding Author:**	Lian Xu, PhD<br>the Second Affiliated Hospital of Nantong University, Nantong University<br>Nantong, CHINA
**Corresponding Author Secondary Information:**	
**Corresponding Author's Institution:**	the Second Affiliated Hospital of Nantong University, Nantong University

Powered by Editorial Manager® and ProduXion Manager® from Aries Systems Corporation

**Corresponding Author's Secondary Institution:**	
**First Author:**	Lian Xu, PhD
**First Author Secondary Information:**	
**Order of Authors:**	Lian Xu, PhD
	Weiming He
	Shuaishuai Tai
	Xiaoli Huang
	Mumu Qin
	Xun Liao
	Yi Jing
	Jian Yang
	Xiaodong Fang
	Jianhua Shi
	Nana Jin
**Order of Authors Secondary Information:**	
**Response to Reviewers:**	4 Feb, 2025<br>To<br>The Editor,<br>GigaScience<br><br>Dear Editor,<br>Thank you for carefully reviewing our manuscript, "VCF2Dis: an ultra-fast and efficient tool to calculate pairwise genetic distance and construct population phylogeny from VCF files" (GIGA-D-24-00393R1).<br><br>First, we appreciate for giving an opportunity to submit our revised manuscript, and also your kindly positive comments and critical suggestions, as well as two reviewers' comments, which have helped us to greatly improved our submitted manuscript as well as our software, VCF2Dis.<br><br>We have carefully read the referees' comments. We would like to express our sincere thanks to the reviewers for their constructive and positive comments. We have addressed all their suggestions, and the manuscript has been edited accordingly, with changes highlighted in red text.<br><br>The major amendments were listed below:<br>1.We have revised and resubmitted the manuscript as the "Technical Note" section.<br>2.The results and discussion on the population phylogeny comparison generated by fastreeR and VCF2Dis have been removed in response to reviewer #1's comments.<br>3.A concise pseudocode for the p-distance calculation and accelerated methods in VCF2Dis has been added to the manuscript in response to reviewer #2's comments.<br><br>We hope that with the amendments made in response to the reviewers' comments, the manuscript is now acceptable for publication in GigaScience.<br><br>Sincerely,<br>Dr. Lian Xu<br>(On behalf of the authors)<br>Institute for translational neuroscience, the Second Affiliated Hospital of Nantong University, Nantong University, Nantong, Jiangsu, 226001, China.<br>Email: xulian@ntu.edu.cn

Powered by Editorial Manager® and ProduXion Manager® from Aries Systems Corporation

Response to comments point by point:

Ekaterina Noskova (Reviewer 1)
1.Reviewer #1: The authors have carefully addressed my previous suggestions and comments, resulting in a much clearer and improved manuscript. This has allowed me to form a more comprehensive opinion about both the manuscript and the tool, VCF2Dis, presented within it. The authors effectively highlight the problem of evaluating distance matrices for large datasets and provide evidence of the limitations in existing tools. Their benchmarking results demonstrate that VCF2Dis is indeed significantly faster than comparable tools.

Response: We sincerely appreciate your thoughtful feedback and positive evaluation of our revised manuscript and VCF2Dis.

2. Despite these improvements, I believe the manuscript lacks a substantial scientific contribution and would not recommend it for publication in GigaScience as a full research article. However, it may be better suited for a shorter format, such as a technical note, similar to how the tool VCF2PopTree was published.

Response: Thank you for your professional suggestions. We did not shorten the paper, as there is no strict length restriction for Technical Notes. We have resubmitted the manuscript as a "Technical Note."

3. In response to my comments, the authors provided detailed explanations of VCF2Dis's implementation, which contributes to its efficiency. These include line-by-line reading of VCF files, pointer-based string operations, eliminating redundant computations using upper triangle matrix evaluations only, and parallelization using OpenMP. While these features contribute to the tool's performance, I find these approaches to be straightforward and relatively standard methods for handling large datasets in C++, rather than technically innovative or advanced.

Response: We greatly appreciate your insightful feedback and your recognition of the key features contributing to VCF2Dis's efficiency. While we agree that methods such as line-by-line reading, pointer-based string operations, and parallelization using OpenMP are common strategies for handling large datasets, our intention was to highlight how their combined implementation in VCF2Dis leads to a significant performance improvement.
In line with your suggestion, we have resubmitted the manuscript as a "Technical Note."

4. The manuscript includes an experiment using a real dataset from the 1000 Genomes Project, where VCF2Dis is compared with fastreeR. The results demonstrate that VCF2Dis produces a more accurate phylogenetic tree. Upon further investigation, the authors determined that the difference arises from the methods used for phylogenetic tree construction, as the distance matrices generated by both tools were identical. I believe this section may be confusing for the reader and requires revision. The main feature of VCF2Dis is its ability to evaluate distance matrices quickly, and I would therefore expect a direct comparison of this step between the two tools. The observed differences in phylogenetic trees and the more accurate results obtained using VCF2Dis could instead be presented as supporting evidence for why the ape package was chosen as the phylogenetic method in VCF2Dis. However, I would argue that a single case is not sufficient to demonstrate the superiority of ape over the method used in fastreeR. Additional experiments and comparisons are necessary to substantiate this claim.

Response: We greatly appreciate your valuable feedback and professional comments. We agree that a single case is not sufficient to conclusively demonstrate the superiority of the ape method over that used in fastreeR. In response to your suggestion, we have removed the results and discussion comparing the population phylogeny generated by VCF2Dis+ape and fastreeR.

Powered by Editorial Manager® and ProduXion Manager® from Aries Systems Corporation

	5. That said, the manuscript serves as a solid technical paper. It identifies a genuine problem—computing distance matrices for large datasets—presents an efficient solution, and provides a fair comparison with existing tools to demonstrate its superiority. While its current contribution may not be sufficient for a full research article, it could still hold value as a Technical Note with some additional experiments or analyses to enhance its impact.<br><br>Response: Thank you for your professional suggestions. We have resubmitted the manuscript as a “Technical Note.”<br><br><br>Reviewer #2:<br>Catia Vaz (Reviewer 2)<br>1.It thank the authors for their answers, but I have still some doubts.<br><br>When the authors say:<br>"For memory efficiency, we employed a strategy by reading and processing in a manner of line-by-line, ensuring that memory usage is affected only by the number of samples and remains independent of the number of variants. For runtime efficiency, the distance complexity is O(n^2 M) ".<br>I would like to see more details in the paper (perhaps a pseudo-code) of how you, in runtime, depend on the number of variants but in memory you don't depend on it. You don't keep it in memory? If so, how you process the distance depending on that? That should be clarified better in my opinion.<br>Also, when you refer:<br>" is not solely due to the programming language but also its optimized implementation."<br>So, what specific optimizations that you have and others don't allow to do this affirmation? Again, a pseudo code with some comments will help to see what is the innovation of your implementation.<br><br>Response: Thank you for your kind comments. The optimized implementation of VCF2Dis incorporates several features, including line-by-line reading of VCF files, pointer-based string operations, eliminating redundant computations through upper triangle matrix evaluations, and parallelization using OpenMP.<br>We have added a pseudo-code and a detailed explanation of the calculation of p-distance and the optimization process to the Supplementary Note 1. Additionally, as per your suggestion, we have now included a concise version of the pseudocode in the manuscript.<br><br><br>2. Can we reproduce your tests to experiment with other datasets, namely the comparison of your approach with the other one that you mention? I am sorry, I did not find that to reproduce on the github.<br><br>Response: Thank you for your kind comments. Due to the large size of the test data, we did not upload it to GitHub previously. However, we have provided all the scripts and test datasets used in this study on the FTP server hosted by GigaDB (the username and password are listed below) to allow others to reproduce the results. Additionally, we have now uploaded the test scripts for downloading and generating the test dataset, as well as the running scripts, to GitHub at https://github.com/hewm2008/VCF2Dis/tree/main/RunTest. Furthermore, this tool has been widely used in real datasets and has been cited more than 203 times (according to a Google Scholar search on February 3, 2025).<br><br>The username and password to access the test dataset and scripts to the ftp provided by GigaDB.<br>username = user270<br>password = SNBjQos8sIubL<br>FTP server = files.gigadb.org<br><br>3. I also thank the authors for making available a docker image to test it.<br><br>Response: Thank you for your professional suggestion again.
**Additional Information:**	

Question	Response
Are you submitting this manuscript to a special series or article collection?	No
**Experimental design and statistics**<br><br>Full details of the experimental design and statistical methods used should be given in the Methods section, as detailed in our Minimum Standards Reporting Checklist. Information essential to interpreting the data presented should be made available in the figure legends.<br><br>Have you included all the information requested in your manuscript?	Yes
**Resources**<br><br>A description of all resources used, including antibodies, cell lines, animals and software tools, with enough information to allow them to be uniquely identified, should be included in the Methods section. Authors are strongly encouraged to cite Research Resource Identifiers (RRIDs) for antibodies, model organisms and tools, where possible.<br><br>Have you included the information requested as detailed in our Minimum Standards Reporting Checklist?	Yes
**Availability of data and materials**<br><br>All datasets and code on which the conclusions of the paper rely must be either included in your submission or deposited in publicly available repositories (where available and ethically appropriate), referencing such data using a unique identifier in the references and in the "Availability of Data and Materials" section of your manuscript.	Yes

Have you have met the above requirement as detailed in our Minimum Standards Reporting Checklist?	
GigaScience has policies and guidelines in place for the use of generative AI-writing tools such as ChatGPT. If you have used such writing tools to assist with writing the manuscript this must be declared and cited in the text. Authors should not list AI-writing tools and other AI-assisted technologies as an author or co-author and should acknowledge that they are fully responsible for text generated or refined by AI-writing tools.<br><br>A summary of use (particularly in the introduction or among methods) needs to be included at the end of the paper, and the outputs should also be included as a supplementary file hosted in GigaDB or other open repositories. Please read our guidelines for more information. <br>By submitting to GigaScience, you are aware of the journal's AI-writing tools policy, and if you have declared use of such tools below, you have acknowledged this where appropriate in your manuscript and have made a summary of use and outputs available. <br><br>**AI-assisted writing tools have been used in the preparation of this manuscript?<br>** 	

Powered by Editorial Manager® and ProduXion Manager® from Aries Systems Corporation

Manuscript
Click here to access/download;Manuscript;VCF2DIS_for_production.docx

VCF2Dis: an ultra-fast and efficient tool to calculate pairwise genetic distance and construct population phylogeny from VCF files

Lian Xu[1,2#], Weiming He[3,4#], Shuaishuai Tai[3], Xiaoli Huang[1], Mumu Qin[4], Xun Liao[3], Yi Jing[4], Jian Yang[2], Xiaodong Fang[3,4], Jianhua Shi[1*], Nana Jin[1,2*]

[1]Institute for translational neuroscience of Affiliated Hospital 2 of Nantong University; Center for neural developmental and degenerative research of Nantong University, Nantong, Jiangsu, 226001, China.

[2]Key Laboratory of Neuroregeneration, Ministry of Education and Jiangsu Province, Co-innovation Center of Neuroregeneration, NMPA Key Laboratory for Research and Evaluation of Tissue Engineering Technology Products, Nantong University, Nantong, Jiangsu, 226001, China.

[3]BGI Research, Shenzhen, 518083, China.

[4]BGI Research, Sanya, 572025, China.

*To whom correspondence: Jianhua Shi (ntshijianhua@ntu.edu.cn) and Nana Jin (yongna0321@126.com).

[#]These authors contributed equally.

ORCID Lian Xu [0000-0003-1685-605X]; Weiming He [0000-0003-0483-5390]; Shuaishuai Tai [0000-0001-8204-6982]; Xiaoli Huang [0009-0003-7923-4386]; Mumu Qin [0009-0008-9940-6225]; Xun Liao [0000-0002-6789-4358]; Yi Jing [0000-0002-5424-9106]; Jian Yang [0000-0001-6318-8854]; Xiaodong Fang [0000-0001-7061-3337]; Jianhua Shi [0000-0002-5351-406X]; Nana Jin [0009-0006-5522-3991]

Abstract

Background: Genetic distance metrics are crucial for understanding the evolutionary relationships and population structure of organisms. The advance of next-generation sequencing technology has given rise of genotyping data of thousands of individuals. The standard Variant Call Format (VCF) is widely used to store genomic variation

information, but calculating genetic distance and constructing population phylogeny directly from large VCF files can be challenging. Moreover, the existing tools that implement such function remains limited and have low performance in processing large-scale genotype data, especially in the area of memory efficiency.

Findings: To address these challenges, we introduce VCF2Dis, an ultra-fast and efficient tool that calculates pairwise genetic distance directly from large VCF files and then constructs distance-based population phylogeny using the ape package. Benchmarking results demonstrate the tool's efficiency, with rapid processing times, minimal memory usage (*e.g.*, 0.37 GB for the whole analysis of 2,504 samples with 81.2 million variants), and high accuracy, even when handling datasets with millions of variants from thousands of individuals. Its straightforward command-line interface, compatibility with downstream phylogenetic analysis tools (such as MEGA, Phylip, and FastTree), and support for multithreading make it a valuable tool for researchers studying population relationships. These advantages meaning VCF2Dis has already been widely utilized in many published genomic studies.

Conclusion: We present VCF2Dis, a straightforward and efficient tool for calculating genetic distance and constructing population phylogeny directly from large-scale genotype data. VCF2Dis has been widely applied, facilitating the exploration of population relationship in extensive genome sequencing studies.

Keywords: VCF2Dis, p-distance, population phylogeny, VCF

Introduction

With the advance and decreased cost of sequencing technologies, increasing amounts of large-scale genome sequencing of individuals has been performed, such as the 1000 Genomes Project, UK Biobank and 3000 Rice Genomes Project [1-3]. These large-scale genome projects generate a large amount of genetic variation, including single nucleotide polymorphisms (SNPs) and insertions/deletions (indels), and are stored in standard Variant Call Format (VCF). These datasets provide tremendous resource for further exploring genetic diversity. Exploring population structure and relationships are fundamental tasks in evolutionary biology and population genetics, requiring robust

methods to infer evolutionary history[4]. Among these methods, distance-based approaches for phylogenetic tree construction, such as neighbor-joining and UPGMA, are computationally efficient and utilize evaluated pairwise distances between genomes to construct trees[4-6]. These methods are particularly well-suited for analyzing large datasets, including those in VCF format, as they do not require sequence alignment. In contrast, another category of phylogenetic tools, such as RAxML[7], IQ-TREE[8], PhyML[9], and FastTree[10], employs maximum likelihood estimation. These tools rely on substitution models to infer phylogenies and require alignment data as input. This class of methods is more complex and provides more accurate evolutionary inferences, but it is generally more computationally intensive. Although capable of handling large sample counts, their applicability is often constrained to gene-level analyses.

Most current tools for constructing population phylogeny from VCF files firstly convert VCF format into an alignment format (e.g., FASTA and “Phy”) and then employ third-party evolutionary phylogenetic software, such as MUSCLE [11], FastME [12], FastTree [10], IQ-TREE [8] and Phylip [13]. These tools include local pipelines or programs, such as SNPhylo [14], VCF-Kit [15], VCFToTree [16], and web-based applications, such as SNiPlay3 [17] and CSI Phylogeny [18]. However, alignment-based methods are computationally demanding and are not well-suited for large-scale genotype datasets due to their high resource consumption, including both computational power and memory.

Currently, two programs, VCF2PopTree [19] and fastreeR [20], are commonly used to calculate genetic distance and then construct distance-based population phylogeny directly from VCF files. VCF2PopTree, a JavaScript-based client-side application, calculates p-distance and constructs a distance-based phylogeny using either the UPGMA or Neighbor-Joining algorithms. While this tool requires minimal memory, its scalability is limited, as it can only process populations with fewer than 1,500 individuals (as inferred from its source code). Furthermore, it is slow and becomes unresponsive when handling a large input file. FastreeR, a R package, implements calculating “cosine” distance and constructs neighbor-joining phylogeny using the Java

programming language. It needs several functions for users to calculate distance, construct phylogeny, and display trees, making it difficult for researchers without advanced programming skills. Furthermore, it is difficult to control memory usage based on Java. Both tools only able to adopt one input file. Many efficient tools for such distance-based phylogeny reconstruction have been developed [6]. Nevertheless, the distance calculation step remains a major bottleneck, especially when processing large-scale genomic datasets. To address these challenges, we developed VCF2Dis, a command-line tool designed to efficiently calculate the p-distance (proportion (p) of nucleotide sites at which two sequences differ[21], **Methods**) matric from single or multiple VCF files with minimal memory consumption (*e.g.*, 0.37 GB for the whole analysis of 2,504 samples with 81.2 million variants) and high computational speed (*e.g.*, 3.48 times and 47.78 times faster than fastreeR and ngsDist, respectively, when calculating the genetic distance for 1,000 individuals with 2 million variants). In addition, it could construct a phylogenetic tree using the UPGMA or the Neighbor-Joining (NJ) method by calling the external ape package[22], and display the tree using the ggtree package[23]. Upon its first release, VCF2Dis has undergone continuous refinement, including running time, and has been cited in many high-quality scientific studies, including studies of population relationships in wheat[24], *Rhesus macaque* [25], lablab [26], and watermelon [27].

Data Description

To evaluate the performance of VCF2Dis, we used the popular dataset from phase 3 of the 1000 Genomes Project which sequenced the genomes of 2,504 individuals from 26 populations and characterized over 88 million variants, including 84.7 million SNPs and 3.6 million indels [28].

Findings

Accuracy and performance of VCF2Dis

VCF2Dis is a simple and straightforward command-line tool that enables users to obtain p-distance matrix directly from one or multiple VCF files, and infer distance-

based population relationship using the external ape package (**Fig. 1A**). For the simplest usage, users only need to provide single or multiple input files via the "-InPut" parameter to quickly generate output files, including a p-distance matrix, a Newick format tree and associated figures in PDF and PNG formats. Additionally, users can reconstruct population phylogeny using other alternative phylogenetic software, such as MEGA, Phylip, and FastTree using the p-distance matrix output from VCF2Dis as input. For advanced or customized visualization, annotation, and management of phylogenetic trees, users can upload the Newick format tree to powerful web-based tools, such as iTOL [29] and Evolview [30], or use the ggtree R package [23].

To test its accuracy, we extracted a small dataset from 2,504 human genomes via the parameter, "-SubPop", which contained 203 individuals and 81.2 million variants. The neighbor-joining phylogeny of this dataset revealed three distinct groups, with individuals from the same super population (YRI: Africa, CEU: European, Asian: CHB and JPT) clustering together (**Fig. 1B**). Notably, individuals from China (CHB) and Japan (JPT) were clearly distinguishable. Since its initial release, it has been used in studies investigating population relationships in various organisms, including wheat[24], *Rhesus macaque* [25], lablab [26], and watermelon [27]. These evidences demonstrate the accuracy and utility of VCF2Dis in population genetic researches.

VCF2Dis is highly memory-efficient, as it processes input files in a line-by-line manner. This approach ensures that memory consumption depends solely on the number of individuals, rather than the total size of the dataset, making it particularly suitable for handling large-scale genotype data. For instance, analyzing 81.2 million variants across 203 individuals required only 0.17 GB of memory. Even when analyzing 2,504 individuals with 81.2 million variants, the memory usage only increased to 0.37 GB, demonstrating that a substantial increase in sample size does not significantly impact memory usage.

VCF2Dis is also exceptionally fast. To speedup runtime, we utilize pointer-based string operations to reduce memory allocation and assignment operation during data parsing. Furthermore, we only calculate upper-triangle matrix to reduce the computational workload by eliminating redundant operations (Methods and

Supplemental Note 1 in Additional file 1). It completed the analysis of 81.2 million variants across 203 individuals in just about 3 hours. To accelerate the analysis of large-scale genotype data, we also provide a multiple threading version of VCF2Dis (named "VCF2Dis_multi") by paralleling *for* loop using OpenMP library. We tested the performance of VCF2Dis_multi in distance calculation step on different thread counts (n=2,4,8,16,32) with a dataset containing 1 million variants across 2,504 samples from the 1000 Genomes Project. The result showed that the runtime generally decreases as the number of threads increases, but the reduction is not perfectly linear (**Fig. S1** in Additional file 1). In these tests, the best speedup was 19-fold achieved using 32 threads that VCF2Dis_multi took 8.1 minutes while the single-threaded VCF2Dis took 157.8 minutes (**Fig. S1** in Additional file 1 and **Table S3** in Additional file 2). We also compared and tested the performance of VCF2Dis and VCF2Dis_multi in distance calculation step across the number of variants and samples. The runtime of both single-threaded and multi-threaded VCF2Dis exhibited a linear relationship with the number of variants (**Fig. S2A** in Additional file 1). In this scenario, the multi-threaded version achieved a speedup of 2-3 times compared to the single-threaded implementation. For the tested sample sizes ranging from 100 to 2,500, the runtime of multi-threaded VCF2Dis demonstrated significant improvement, achieving over an 11-fold speedup when the sample size exceeded 600 (**Fig. S2B** in Additional File 1 and **Table S2** in Additional file 2). Therefore, the multi-threaded VCF2Dis is highly suitable for analyzing large-scale genomic datasets, particularly those involving thousands of individuals.

Performance comparison with other existing tools

Two tools, VCF2PopTree and fastreeR offer functions for pairwise distance calculation and constructing population phylogeny directly from VCF files (**Table 1**). However, VCF2PopTree, a JavaScript-based local client program, failed to process datasets with a large number of samples and variants (*e.g.*, 91 samples with 3M variants). We also found another tool, ngsDist[31], developed in C/C++, which is capable of calculating p-distance. However, this tool requires an additional preprocessing step - converting

VCF format into PLINK format - to function correctly (**Table 1**). The runtime complexity of VCF2Dis is primarily determined by the distance calculation step (see Methods for details). Additionally, since VCF2Dis focuses on p-distance while tree reconstruction is handled by an external tool, we compared the performance of VCF2Dis, fastreeR and ngsDist in terms of runtime and memory usage during the distance calculation process. We also consider the number of samples and the number of variants on the performance (**Methods**).

In terms of memory usage, VCF2Dis consumed extremely low memory compared to other two tools (**Fig. 2A** and **2C**). For example, VCF2Dis required only 10 MB of memory to analyze 1,000 samples with 2 million variants, whereas fastreeR and ngsDist consumed 55.36 GB and 92.83 GB, respectively (**Table S1** in the Additional file 2). The memory usage of VCF2Dis is independent of the number of variants and increases slightly with the number of samples (**Fig. 2**). In contrast, the memory usage of fastreeR approximately follows a logarithmic increase with the number of variants and samples, whereas ngsDist exhibits a linear relationship with both the number of samples and the number of variants.

In terms of runtime performance, the runtime of all three tools shows a linear increase with the number of variants. In this situation, VCF2Dis demonstrates the fastest performance, being approximately 12 times and 36 times faster than fastreeR and ngsDist, respectively (**Fig. 2B**). Regarding the number of samples, the runtime of all three tools approximately follows a pattern where the time taken is proportional to the square of the sample size. However, VCF2Dis showed the fastest performance, being approximately 3 times and 45 times faster than fastreeR and ngsDist, respectively (**Fig. 2D**). For instance, when analyzing 1,000 individuals, VCF2Dis took 49.84 seconds, while fastreeR took 173.64 seconds and ngsDist took 2,381.68 seconds, which is approximately 3.48 times and 47.78 times faster (**Table S1** in Additional file 2), respectively. Therefore, VCF2Dis consistently outpaced fastreeR and ngsDist, particularly as the sample size increased.

Unlike VCF2Dis, which uses the p-distance method and fastreeR employs a “cosine” distance metric. To compare the accuracy of the two software, we conducted a test using

203 individuals with 3,492 variants from the 1000 Genomes Project which was included as a test dataset used in VCF2PCACluster software [32]. Our results showed that both tools produced identical distance values. Overall, these comparisons highlight the accuracy and high performance of VCF2Dis in handing large-scale population genetics analyses.

Discussion

VCF2Dis is a simple and efficient tool designed to facilitate the calculation of genetic distance and reconstruction of population relationships directly from large VCF files, offering significant advantages for large-scale genomic studies. Since its first release, it has been widely applied and cited in studies of population relationships, such as wheat[24], *Rhesus macaque* [25], lablab [26], and watermelon [27]. One of the key strengths of VCF2Dis lies in its ability to calculate p-distance quickly with extremely low running memory, even for large datasets involving thousands of individuals. This is especially useful given the increasing size of population genomic datasets generated by projects such as the UKB whole-genome sequencing (WGS) consortium and other large-scale sequencing efforts [2, 33]. The integration of multithreading further enhances its performance, providing significant time savings in computationally intensive tasks, as demonstrated by its 19-fold speed improvement over single-threaded execution in our benchmarking tests of 2,504 samples with 1 million variants using 32 threads. It is important to note that the speedup achieved by "VCF2Dis_multi" is often nonlinear compared to the single-threaded version of VCF2Dis. Factors such as the overhead of thread management, uneven workload distribution among threads, and the fact that not all steps (*e.g.*, I/O) in the process are fully parallelizable can impact parallel efficiency. Consequently, we recommend employing the multi-threaded version of VCF2Dis for studies involving thousands of individuals, as it provides substantial computational advantages.

In addition to its efficiency, VCF2Dis offers flexibility. The output files, including p-distance matrices and the Newick format tree, can be easily used as inputs for other

popular phylogenetic analysis tools like MEGA [21], Phylip, and FastTree, allowing users to build and refine their phylogenetic tree using a variety of software. Moreover, for users who require more advanced visualization and annotation capabilities, the compatibility with tools such as iTOL, Evolview, and the ggtree R package provides extensive options for tree manipulation and display.

However, some limitations should also be considered in the future work. First, VCF2Dis is highly effective for generating p-distance matrices and its utility is dependent on the quality of the input VCF data. In cases where the VCF contains missing or erroneous data, the resulting distance matrix and phylogenetic tree may not accurately reflect the true population structure. Secondly, the current version of VCF2Dis focuses solely on p-distance, which may not be the best metric for all phylogenetic analyses. Future incorporation of additional genetic distance metrics could expand the functionality of VCF2Dis and enhance its applicability to a broader range of evolutionary studies. Thirdly, future developments of VCF2Dis could also address user needs for more interactive features, such as a graphical user interface (GUI), which would lower the entry barrier for non-technical users. Although VCF2PopTree was not included in the performance comparison due to its failure in most tests, its user-friendly interface, which requires just one click, makes it a viable option for scientific experts without advanced computational skills, particularly for the analysis of small datasets.

In conclusion, VCF2Dis provides a valuable tool for researchers conducting large-scale population genetic studies, offering a fast, flexible, and user-friendly solution for generating p-distance matrices and constructing population phylogenies from VCF files. It enables users to infer distance-based population phylogeny directly from VCF files, significantly streamlining the workflow. Despite some limitations, it remains a powerful option for users seeking to streamline their phylogenetic analysis workflows.

Methods

Overview of VCF2Dis workflow

VCF2Dis (RRID:SCR_022513) is implemented with C/C++ and R programming languages, and runs on Linux/Unix and MacOS operating systems. The C/C++

components are mainly used for computational tasks, while R is utilized for generating visualizations (**Fig. 1A**). We have also provided both Docker and Singularity containerized versions of VCF2Dis, enabling users to bypass the compilation and installation process for a seamless experience. VCF2Dis can utilize compressed or uncompressed input files with formats of VCF, fasta, and "phy", via "-InPut" and "-InFormat" parameters. Users can provide one or several input files separated by a space or provide a list file with path of input files via "-InList" parameter. Specifically, VCF2Dis can analyze bgzipped/gzip VCF files which allows random access and widely used in big genomic data storage and search. By default, VCF2Dis performs calculation for all samples defined in the input. Recognizing the common need in population genetics to construct phylogenies for specific sub-populations, we provide the '-SubPop' parameter. This feature enables users to easily generate trees for selected sample subsets by specifying them through this parameter. For input of "phy" format, it is firstly converted into fasta format and then calculates p-distance. VCF2Dis employs an external R package, ape [22], to construct population phylogeny and users could choose neighbor-joining or UPGMA algorithms via "-TreeMethod" parameter. To meet the requirement of showing bootstrap values on the branch of phylogeny for some users, we also employed a method of sampling with replacement. For this scenario, users can randomly set a certain ratio (default: 0.25) of all the sites via the parameter, "-Rand", and run VCF2Dis with given repeated times, such as 100 times, to separately construct trees. After that, trees are combined and subject to the fconsense program implemented in the PHYLIPNEW package [34] to construct a consensus tree with bootstrap values. In addition, VCF2Dis employs another R package, ggtree [23], to provide an initial display of population relationship. Users could optionally provide prior group information of individuals for color labelling in the tree figure via "-InSampleGroup" parameter. The outputs of VCF2Dis include p-distance matrix, phylogeny in Newick format, and related figures in PDF and PNG formats. With the output of p-distance matrix, users could use other phylogenomic software to reconstruct population phylogeny, such as MEGA (RRID:SCR_000667) [21], FastMe 2.0 [12], Phylip (RRID:SCR_006244) [13], and PHYLIPNEW package [34]. For advanced and customized visualization of the phylogeny, users can set additional attributes (*e.g.*, layout, color, shape) and modify in our provided custom R script for tree display or use other alternative excellent online or localized interactive tools, such as iTOL [29], Evolview [30] and MEGA [21].

The p-distance calculation

The p-distance is a straightforward approach to estimate genetic distance between two genomes [21]. For genotyping data, the following formula is used to calculate distance (D_{ij}) for individual i and j with the total length of L where variants can be identified:

$$D_{ij} = \frac{\sum_{l=1}^{L} d_l}{L}$$

For instance, assuming alleles at the position l are A/C and d_l could be set as followings:

If genotypes of two individuals are the same (AA, CC, or AC) then d_l = 0;

If genotypes of two individuals are AA and AC respectively, then $d_l = 0.5$;

If genotypes of two individuals are AA and CC respectively, then $d_l = 1$.

Only bi-allelic variants are considered by most genetic distance calculation tools, such as Vcf2popTree and PLINK. However, multiallelic variants are frequent in population and ignoration could lead to loss of effective genetic information. Thus, we didn't perform any preprocessing of VCF files and compared their genotypes. We adopt a site-by-site of pairwise distance calculation and summed them into a total dissimilarity of the whole genome, namely pairwise distance matrix, which is subjected to external phylogenetic software (ape) for population phylogeny construction. Furthermore, VCF2Dis also considers genotype data from phased genomes. In phased genomes:

if genotypes of two individuals are AC and AC respectively, then $d_l = 0$;

if genotypes of two individuals are CA and AC respectively, then $d_l = 1$;

See **Algorithm 1** for the pseudocode of the p-distance calculation and see **Supplementary Note 1** for more details.

Accelerated methods of VCF2Dis

Large-scale genome sequencing projects generate millions of variants across hundreds of accessions, leading to an extensive memory usage and long runtime. For instance, the popular tool PLINK [35] (v1.9) can require more than 257 GB of memory when analyzing a large dataset containing 78 million biallelic SNPs across 2500 human genomes[32], which is challenging to run on a standard computer. To address memory

concerns, VCF2Dis adopts a streaming processing approach, reading and calculating data line-by-line rather than loading the entire VCF file into memory before processing. This method enables efficient handling of large dataset within minimal memory usage (e.g., less than 0.1 GB for analyzing 2500 individuals in distance calculation step). To accelerate its runtime, we have made two major improvements during data processing. First, we utilized pointer-based string operations, reducing overhead associated with memory allocation and assignment operation during data parsing. This results in faster extraction of relevant fields from input files, as compared to traditional string manipulation methods. Furthermore, we have optimized the computation process by employing upper-triangle calculations, which significantly reduce the computational workload by eliminating redundant operations. These optimizations ensure that VCF2Dis is both faster and more memory-efficient. See **Algorithm 1** for the pseudocode of the accelerated methods and see **Supplementary Note 1** for more details. In addition, we also implemented a multiple thread version of VCF2Dis ("VCF2Dis_multi") by paralleling *for* loop using OpenMP library [36].

```
## Pointer-based operations ##
void split2(const string& str, std::vector<const char*>& tokens, int
VecSizeNum)
{
        string::size_type lastPos = 0;
        string::size_type pos =1;
        const char* strPtr = str.c_str();
        for (int k = 0; k < VecSizeNum; k++)
        {
                pos = lastPos + 1;
                tokens[k] = (strPtr + pos);
                lastPos = str.find('\t', pos);
        }
}

####### main ####
open VCF file
sample_count = get_sample_count(VCF_file)       # Get the number of samples
diff_matrix[sample_count][sample_count] = 0   # Matrix to store the number of differences by
pairwise comparison
total_matrix[sample_count][sample_count] = 0  # Matrix to store the total number of variants


while (read each line in VCF file)
{
    fields = split2(line)     # Time complexity improved from O(n * m) to O(n)
                              # optimized for runtime performance

    # Compare upper triangle only to reduce computations
    # Traverse sample pairs to calculate p-distance
```

```
    for sample j from 0 to (sample_count – 1)
    {
        for sample k from (j + 1) to (sample_count – 1)
        {
                total_matrix[j][k] += 2
            if fields[j][0] != fields[k][0]  then  #8-wide SIMD char register
                   diff_matrix[j][k] +=1
            if fields[j][2] != fields[k][2]  then
                   diff_matrix[j][k] += 1

        }
     }
}
close VCF file

p_distance = diff_matrix / total_matrix    # Calculate p-distance
return p_distance
```

Algorithm 1: The calculation of p-distance and the main accelerated methods in VCF2Dis.

The runtime complexity of VCF2Dis

The runtime complexity of VCF2Dis is primarily determined by two main components: p-distance matrix calculation and tree construction. For p-distance matrix calculation, this step has a complexity of $\mathrm{O}(n^2\ m)$, where n represents the number of samples and m represents the number of variants. Each pair of samples requires a comparison across m variants. For tree construction step, the Neighbor-Joining method used for tree construction has a complexity of $\mathrm{O}(n^3)$, as it involves iterative clustering of n samples. The overall runtime complexity is therefore $O(n^2\ m) + O(n^3)$. Given that m (commonly >10^6) is typically much larger than n (commonly <10^3), the runtime complexity is predominantly determined by the p-distance matrix calculation step, making it is nearly $O(n^2\ m)$ of VCF2Dis in practical scenarios.

Evaluation of performance in memory usage and runtime of existing tools

To evaluate performance, we assessed the memory usage and runtime of existing tools, VCF2Dis, fastreeR and ngsDist, which are designed for calculating genetic distance and/or reconstruct distance-based population phylogeny. fastreeR was installed via the Bioconductor package, while ngsDist was downloaded from its GitHub repository (https://github.com/fgvieira/ngsDist). Test datasets were generated from the 1000

Genome Project. To evaluate the number of samples on performance, we used a dataset containing 2 million variants across 2,504 individuals from the 1000 Genome Project. However, fastreeR was unable to complete the calculations within a reasonable timeframe, while ngsDist consumed excessive memory resources and was terminated by the system when processing datasets with more than 1,000 samples. Consequently, we conducted performance tests on datasets with fewer than 1,000 samples (100, 200, 300, …, up to 1000), each containing 2 million variants. To evaluate the effect of the number of variants on performance, datasets were created with fixed 91 samples, containing 1 million, 2 million, 3 million, …, up to 10 million variants each. The tools were executed according to their respective documentation, and the memory usage and runtime of completed jobs were recorded. Results were visualized using the ggplot2 package and have been shown in Additional file 2. All evaluations were performed on a computational node with 64 cores and 512 GB of memory, managed using the qsub job scheduler.

Additional Files

Additional file 1: Figure S1. The performance of multi-threaded VCF2Dis (VCF2Dis_multi) with different thread counts**. Figure S2.** The performance of multi-threaded VCF2Dis (VCF2Dis_multi) with different number of variants and samples compared to the single-threaded VCF2Dis. **Supplementary Note 1**: The pseudocode for improving memory and runtime of VCF2Dis.

Additional file 2: Table S1. The performance comparison of the distance calculation step using VCF2Dis, fastreeR, and ngsDist was conducted across varying numbers of variants and individuals. **Table S2.** The performance comparison of the distance calculation step between VCF2Dis_multi and VCF2Dis was conducted across varying sample sizes and numbers of variants. **Table S3.** The performance of the distance calculation step in the multi-threaded version of VCF2Dis (VCF2Dis_multi) was evaluated using different thread counts.

Abbreviations

VCF: Variant Call Format; VCF2Dis: Variant Call Format to distance; SNP: Single-Nucleotide Polymorphism; Indel: insertion/deletion; M: million; GB: Gigabyte; MB: Megabyte; NJ: Neighbor-Joining; UPGMA: unweighted pair group method with arithmetic mean.

Availability of Source Code and Requirements

Project name: VCF2Dis
Project homepage: https://github.com/hewm2008/VCF2Dis
Operating systems(s): Linux/Unix, MacOS
Programming language: C/C++, R
License: MIT License
RRID:SCR_022513
VCF2Dis requires minimal external dependencies, making installation simple. It can generate the p-distance matrix without R or related packages, though the visualization features will not be available in this case.

Data Availability

The datasets used in this study are freely available from the 1000 Genome Project-Phase 3 dataset [3], [37]. All scripts for the tests, including downloading, generating small datasets, and running, are available in the github repository[38]. An archival copy of the code is available via Software Heritage [39].

Acknowledgments

We thank Longfei Wang for his help in producing a Docker image for VCF2Dis.

Author Contributions

NNJ, JHS and WMH conceived the study. WMH developed the tool and performed the analysis. LX, NNJ and JHS provided suggestion for software improvement. LX wrote the draft manuscript. SST, XLH, MMQ, XL, JY, YJ, and XDF involved in the

discussion and contributed to manuscript. All authors read and approved the final manuscript.

Funding

This work was supported by the National Natural Science Foundation of China (Grant No. 82171425), the Scientific Research Foundation for High-Level Talents of the Second Affiliated Hospital of Nantong University (Grant No. YJRCJJ001 and YJRCJJ004), the Shuangchuang Doctor program of Jiangsu Province (Grant No. JSSCBS20211127), Hainan Seed Industry Laboratory (JBGS-B23YQ2001, JBGS-B23YQ201P) and Project of Sanya Yazhou Bay Science and Technology City, Grant No: (SKJC-2023-02-002).

Competing Interests

The authors declare no potential competing interests.

Table 1. The comparison of VCF2Dis and other distance-based tools

Software	Programming*	Input format			Multiple	Sub-	Algorithm		Output			Memory
		VCF	FASTA	Phy	input files	population	Distance	Tree	figure	distance matrix	newick tree	
VCF2Dis	C/C++	√	√	√	√	√	p-distance	NJ,UPGMA	√	√	√	low
VCF2PopTree	JavaScript	√	×	×	×	√	p-distance	NJ,UPGMA	√	√	√	low
fastreeR	Java	√	√	×	×	×	cosine distance#	NJ	√	√	√	high
ngsDist	C/C++	×	×	×	×	×	p-distance	×	×	√	×	high

*Major programming languages. #defined in the fastreeR. Red text indicated the tool developed in this study.

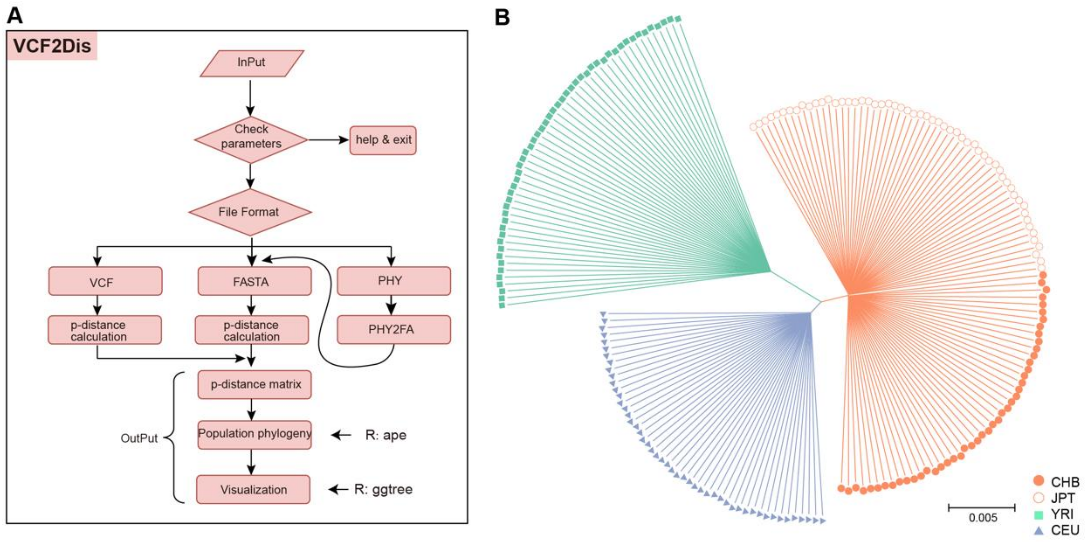

Figure 1. The workflow of VCF2Dis and neighbor-joining phylogeny generated from a test dataset consisting 203 samples and 81.2 million bi-allele SNPs isolated from the 1000 human genomes. **A**, The VCF2Dis workflow involves several key steps, including parameter checks (*e.g.*, input format), p-distance calculation, construction of population phylogeny and phylogeny visualization. VCF2Dis could adopt input with formats of VCF, fasta and “phy”. The outputs include a p-distance matrix, a population phylogeny in newick format and associated figure. **B**, Neighbor-joining phylogeny of 203 individuals. Colors indicated individuals from distinct populations. YRI: Africa; CEU: European; CHB: China; JPT: Japan.

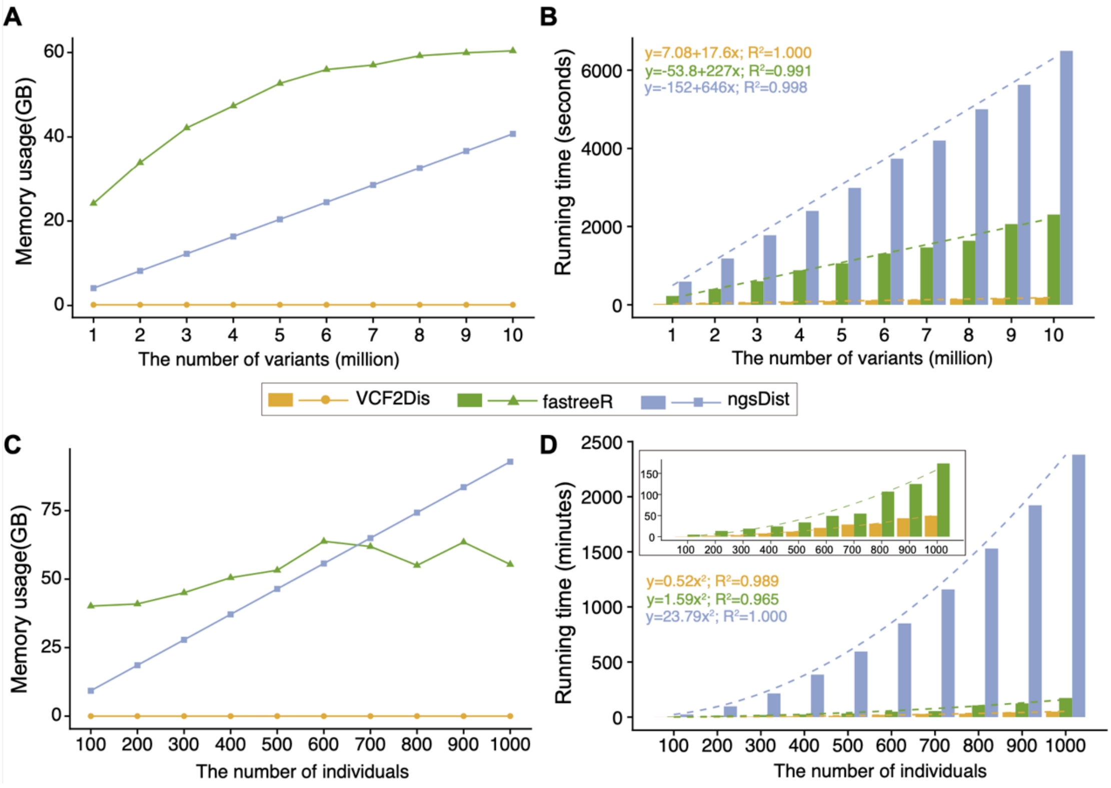

Figure 2. The memory and runtime performance of VCF2Dis, fastreeR, and ngsDist were assessed based on the number of variants and samples in calculating genetic distance. A, The memory test with an increasing number of variants in a dataset containing 91 samples. **B**, The runtime test with an increasing number of variants in a dataset containing 91 samples. **C**, The memory test with an increasing number of individuals, each containing 2 million variants. **D**, The runtime test with an increasing number of individuals, each containing 2 million variants. The runtime of VCF2Dis and fastreeR were also separately shown in the inner box.

References

1. Palmer LJ. UK Biobank: bank on it. Lancet. 2007;369 9578:1980-2. doi:10.1016/S0140-6736(07)60924-6.
2. The 3,000 rice genomes project. The 3,000 rice genomes project. Gigascience. 2014;3:7. doi:10.1186/2047-217X-3-7.
3. Siva N. 1000 Genomes project. Nat Biotechnol. 2008;26 3:256. doi:10.1038/nbt0308-256b.
4. Holder M and Lewis PO. Phylogeny estimation: traditional and Bayesian approaches. Nat Rev Genet. 2003;4 4:275-84. doi:10.1038/nrg1044.
5. Pardi F and Gascuel O. Combinatorics of distance-based tree inference. Proc Natl Acad Sci U S A. 2012;109 41:16443-8. doi:10.1073/pnas.1118368109.
6. Vaz C, Nascimento M, Carrico JA, Rocher T and Francisco AP. Distance-based phylogenetic inference from typing data: a unifying view. Brief Bioinform. 2021;22 3 doi:10.1093/bib/bbaa147.
7. Stamatakis A. RAxML version 8: a tool for phylogenetic analysis and post-analysis of large phylogenies. Bioinformatics. 2014;30 9:1312-3. doi:10.1093/bioinformatics/btu033.
8. Minh BQ, Schmidt HA, Chernomor O, Schrempf D, Woodhams MD, von Haeseler A and Lanfear R. IQ-TREE 2: New Models and Efficient Methods for Phylogenetic Inference in the Genomic Era. Mol Biol Evol. 2020;37 5:1530-4. doi:10.1093/molbev/msaa015.
9. Guindon S, Dufayard JF, Lefort V, Anisimova M, Hordijk W and Gascuel O. New algorithms and methods to estimate maximum-likelihood phylogenies: assessing the performance of PhyML 3.0. Syst Biol. 2010;59 3:307-21. doi:10.1093/sysbio/syq010.
10. Price MN, Dehal PS and Arkin AP. FastTree 2--approximately maximum-likelihood trees for large alignments. PLoS One. 2010;5 3:e9490. doi:10.1371/journal.pone.0009490.
11. Edgar RC. MUSCLE: multiple sequence alignment with high accuracy and high throughput. Nucleic Acids Res. 2004;32 5:1792-7. doi:10.1093/nar/gkh340.
12. Lefort V, Desper R and Gascuel O. FastME 2.0: A Comprehensive, Accurate, and Fast Distance-Based Phylogeny Inference Program. Mol Biol Evol. 2015;32 10:2798-800. doi:10.1093/molbev/msv150.
13. Felsenstein J. PHYLIP (phylogeny inference package), version 3.5 c. Joseph Felsenstein.; 1993.
14. Lee TH, Guo H, Wang X, Kim C and Paterson AH. SNPhylo: a pipeline to construct a phylogenetic tree from huge SNP data. BMC Genomics. 2014;15:162. doi:10.1186/1471-2164-15-162.
15. Cook DE and Andersen EC. VCF-kit: assorted utilities for the variant call format. Bioinformatics. 2017;33 10:1581-2. doi:10.1093/bioinformatics/btx011.
16. Xu D, Jaber Y, Pavlidis P and Gokcumen O. VCFtoTree: a user-friendly tool to construct locus-specific alignments and phylogenies from thousands of anthropologically relevant genome sequences. BMC Bioinformatics. 2017;18 1:426. doi:10.1186/s12859-017-1844-0.
17. Dereeper A, Homa F, Andres G, Sempere G, Sarah G, Hueber Y, et al. SNiPlay3: a web-based application for exploration and large scale analyses of genomic variations. Nucleic Acids Res. 2015;43 W1:W295-300. doi:10.1093/nar/gkv351.
18. Kaas RS, Leekitcharoenphon P, Aarestrup FM and Lund O. Solving the problem of comparing whole bacterial genomes across different sequencing platforms. PLoS One. 2014;9 8:e104984. doi:10.1371/journal.pone.0104984.
19. Subramanian S, Ramasamy U and Chen D. VCF2PopTree: a client-side software to construct

population phylogeny from genome-wide SNPs. PeerJ. 2019;7:e8213. doi:10.7717/peerj.8213.

20. Gkanogiannis A. fastreeR: Phylogenetic, Distance and Other Calculations on VCF and Fasta Files. 2024.

21. Tamura K, Dudley J, Nei M and Kumar S. MEGA4: molecular evolutionary genetics analysis (MEGA) software version 4.0. Molecular biology and evolution. 2007;24 8:1596-9.

22. Paradis E and Schliep K. ape 5.0: an environment for modern phylogenetics and evolutionary analyses in R. Bioinformatics. 2019;35 3:526-8. doi:10.1093/bioinformatics/bty633.

23. Xu S, Li L, Luo X, Chen M, Tang W, Zhan L, et al. Ggtree: A serialized data object for visualization of a phylogenetic tree and annotation data. Imeta. 2022;1 4:e56. doi:10.1002/imt2.56.

24. Cheng S, Feng C, Wingen LU, Cheng H, Riche AB, Jiang M, et al. Harnessing landrace diversity empowers wheat breeding. Nature. 2024;632 8026:823-31. doi:10.1038/s41586-024-07682-9.

25. Ding W, Li X, Zhang J, Ji M, Zhang M, Zhong X, et al. Adaptive functions of structural variants in human brain development. Sci Adv. 2024;10 14:eadl4600. doi:10.1126/sciadv.adl4600.

26. Njaci I, Waweru B, Kamal N, Muktar MS, Fisher D, Gundlach H, et al. Chromosome-level genome assembly and population genomic resource to accelerate orphan crop lablab breeding. Nat Commun. 2023;14 1:1915. doi:10.1038/s41467-023-37489-7.

27. Zhang Y, Zhao M, Tan J, Huang M, Chu X, Li Y, et al. Telomere-to-telomere Citrullus super-pangenome provides direction for watermelon breeding. Nat Genet. 2024;56 8:1750-61. doi:10.1038/s41588-024-01823-6.

28. The 1000 Genomes Project Consortium. A global reference for human genetic variation. Nature. 2015;526 7571:68-74. doi:10.1038/nature15393.

29. Letunic I and Bork P. Interactive Tree of Life (iTOL) v6: recent updates to the phylogenetic tree display and annotation tool. Nucleic Acids Res. 2024;52 W1:W78-W82. doi:10.1093/nar/gkae268.

30. Subramanian B, Gao S, Lercher MJ, Hu S and Chen WH. Evolview v3: a webserver for visualization, annotation, and management of phylogenetic trees. Nucleic Acids Res. 2019;47 W1:W270-W5. doi:10.1093/nar/gkz357.

31. Vieira FG, Lassalle F, Korneliussen TS and Fumagalli M. Improving the estimation of genetic distances from Next-Generation Sequencing data. Biological journal of the Linnean Society. 2016;117 1:139-49.

32. He W, Xu L, Wang J, Yue Z, Jing Y, Tai S, et al. VCF2PCACluster: a simple, fast and memory-efficient tool for principal component analysis of tens of millions of SNPs. BMC Bioinformatics. 2024;25 1:173. doi:10.1186/s12859-024-05770-1.

33. Halldorsson BV, Eggertsson HP, Moore KHS, Hauswedell H, Eiriksson O, Ulfarsson MO, et al. The sequences of 150,119 genomes in the UK Biobank. Nature. 2022;607 7920:732-40. doi:10.1038/s41586-022-04965-x.

34. Rice P, Longden I and Bleasby A. EMBOSS: the European Molecular Biology Open Software Suite. Trends Genet. 2000;16 6:276-7. doi:10.1016/s0168-9525(00)02024-2.

35. Chang CC, Chow CC, Tellier LC, Vattikuti S, Purcell SM and Lee JJ. Second-generation PLINK: rising to the challenge of larger and richer datasets. Gigascience. 2015;4:7. doi:10.1186/s13742-015-0047-8.

36. Dagum L and Menon R. OpenMP: an industry standard API for shared-memory programming. IEEE computational science and engineering. 1998;5 1:46-55.

37. 1000 Genome Project. Phase 3 dataset. [FTP site] https://ftp.1000genomes.ebi.ac.uk/vol1/ftp/release/20130502/
38. VCF2Dis: Run tests [github repository] https://github.com/hewm2008/VCF2Dis/tree/main/RunTest/
39. Xu L, He W, Tai S, Huang X, Qin M et al. VCF2Dis: an ultra-fast and efficient tool to calculate pairwise genetic distance and construct population phylogeny from VCF files (Version 1). [Computer software]. Software Heritage. 2025. https://archive.softwareheritage.org/swh:1:snp:c11bc2f184568c6555793b7e2fdcdc31ffbb28d1;origin=https://github.com/hewm2008/VCF2Dis.git

 Click here to access/download;Table;Table1.xlsx

Software	Programming*	Input format			Multiple input files	Sub-population
		VCF	FASTA	Phy		
VCF2Dis	C/C++	√	√	√	√	√
VCF2PopTree	JavaScript	√	×	×	×	√
fastreeR	Java	√	√	×	×	×
ngsDist	C/C++	×	×	×	×	×

*Major programming languages. #defined in the fastreeR. Red text indicated the tool de

**Algorithm**		**Output**			**Memory**
**Distance**	**Tree**	**figure**	**distance matrix**	**newick tree**	
p-distance	NJ,UPGMA	√	√	√	low
p-distance	NJ,UPGMA	√	√	√	low
cosine distance#	NJ	√	√	√	high
p-distance	×	×	√	×	high

eveloped in this study.

Figure 1

Click here to access/download;Figure;Figure1.pdf

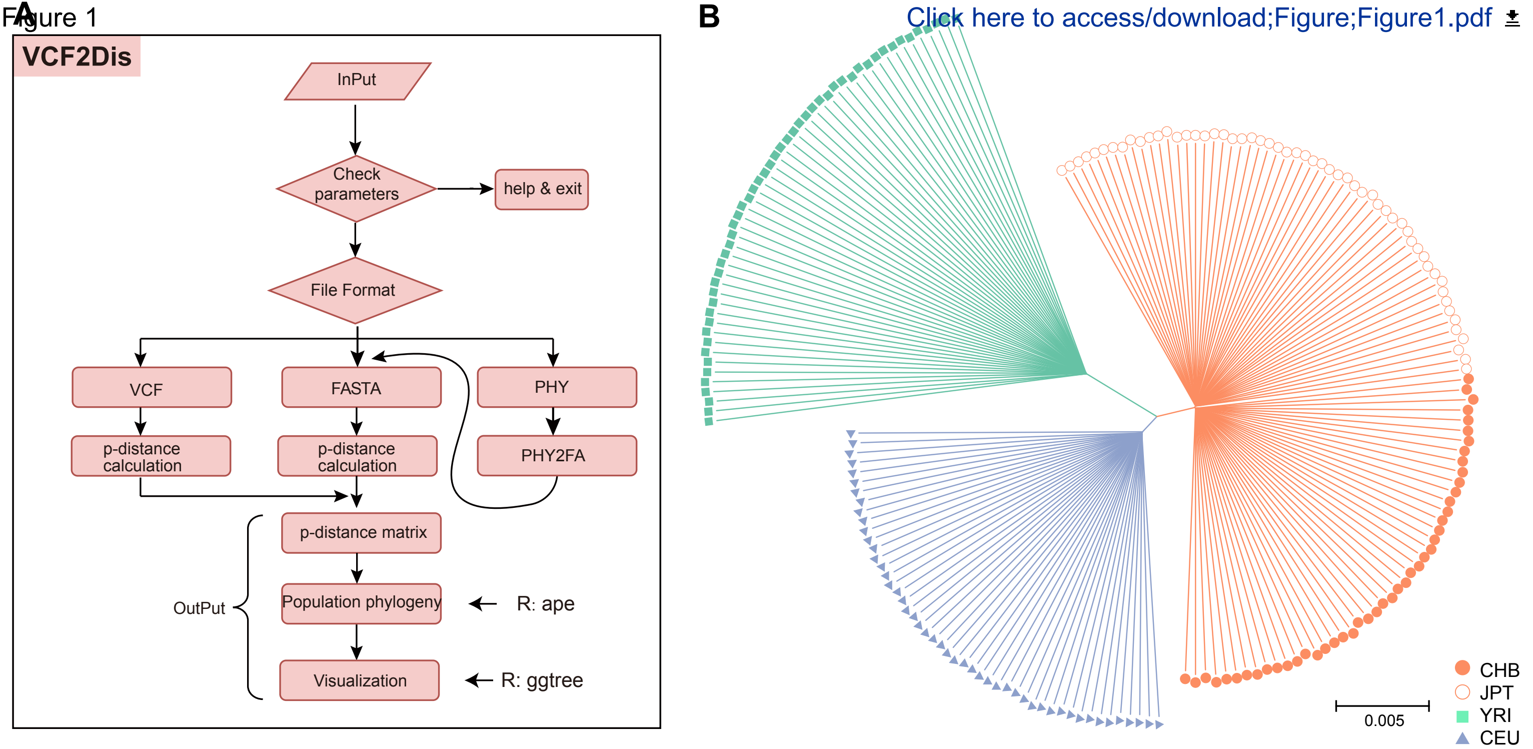

Figure 2

Click here to access/download;Figure;Figure2.pdf

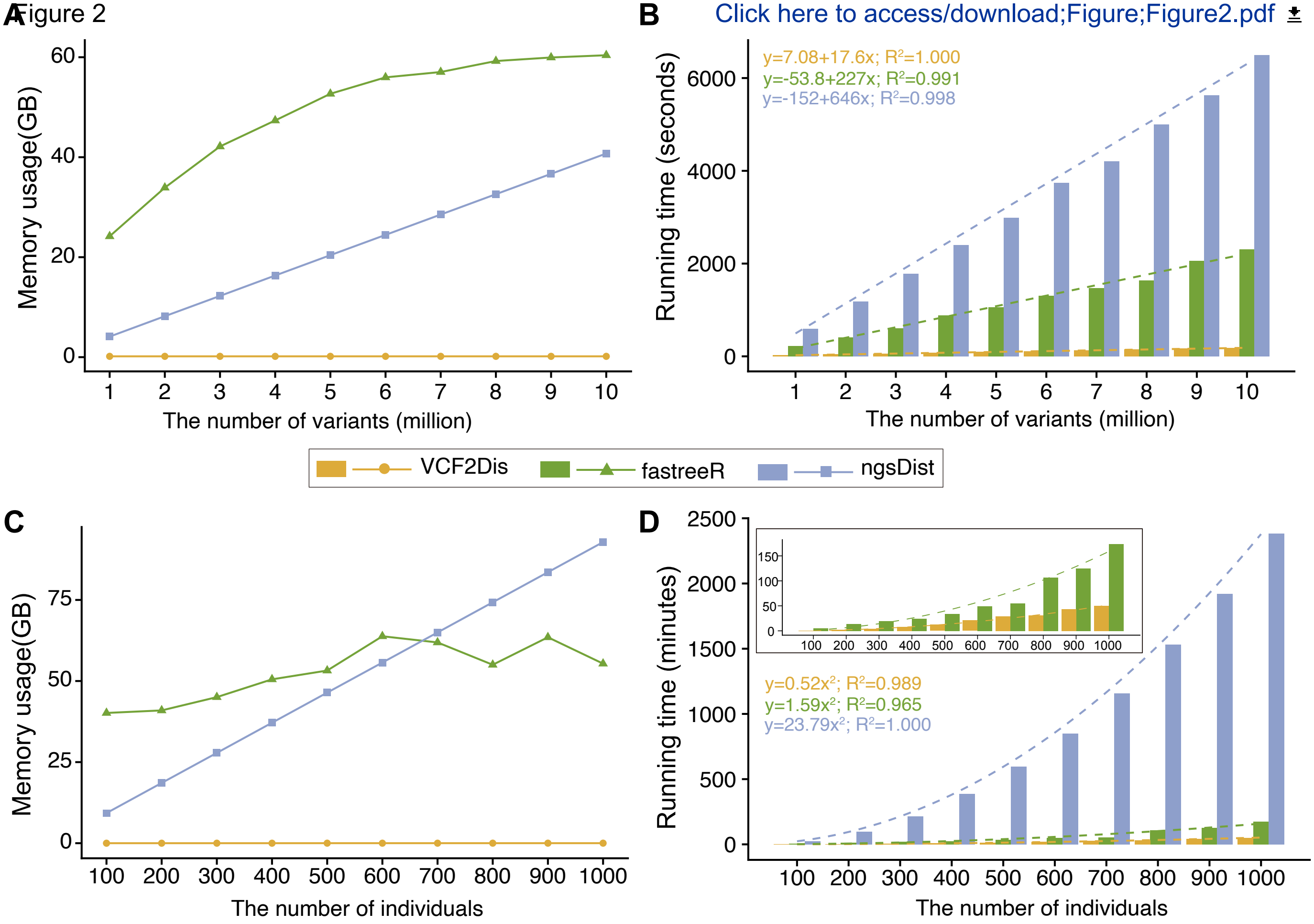

Click here to access/download
Supplementary Material
Addtionally_File1-revise.docx

Tables S1-S3 for performance of running test

Click here to access/download
Supplementary Material
Additional file 2.xlsx
