# Supplementary material for: VCF2Dis: an ultra-fast and efficient tool to calculate pairwise genetic distance and construct population phylogeny from VCF files
Source: Gigascience. 2025 Apr 4;14:giaf032. doi: 10.1093/gigascience/giaf032 (PMC11970368; doi:10.1093/gigascience/giaf032)
Supplement: giaf032_Response_to_Reviewer_Comments_Original_Submission [file giaf032_response_to_reviewer_comments_original_submission.pdf]

Author's Response To Reviewer Comments

10 Jan, 2025
To
The Editor,
GigaScience

Dear Editor,
Thank you for carefully reviewing our manuscript, “VCF2Dis: an ultra-fast and efficient tool to calculate pairwise genetic distance and construct population phylogeny from VCF files” (GIGA-D-24-00393).

First, we appreciate for giving an opportunity to submit our revised manuscript, and also your kindly positive comments and critical suggestions, as well as three reviewers’ comments, which have helped us to greatly improved our submitted manuscript as well as our software, VCF2Dis.

We have carefully read the referees’ comments. We would like to express our sincere thanks to the reviewers for their constructive and positive comments. We have addressed all their suggestions, and the manuscript has been edited accordingly, with changes highlighted in red text. Given the lost highlights and supplemented figures in the response text box, we have also uploaded a Word file (coverLetter_Response) containing the cover letter and response as a supplemental material.

The major amendments were listed below:
1) The readily executable version of VCF2Dis has been packaged in popular Docker and Singularity containers, which are available on GitHub (https://github.com/hewm2008/VCF2Dis). Users can easily pull the images and directly run VCF2Dis without the need for compilation and installation. Detailed instructions are provided in the GitHub repository.
2) Regarding the inclusion of VCF2PopTree for comparison, we conducted tests on a windows 10 computer (256GB memory, 24 threads, i9 10920x CPU) using a small dataset consisting of 91 samples with 1M, 2M and 3M variants. The source code of VCF2PopTree was slightly modified to include a function for displaying the runtime of the distance calculation step. The runtime for the 1M and 2M variants were 456 s and 596 s respectively. However, VCF2PopTree failed to respond when analyzing the 3M variants, even after 24 hours (a screenshot has been shown in the figure listed below, the figure was shown in the coverLetter_Response file which was uploaded as a attached supplementary material). Similar tests on a Mac M1 max computer yielded a runtime of 111 seconds for the 1M dataset, while the software failed to process the 2M and 3M variants. A similar issue has also been reported by a VCF2PopTree user (https://github.com/sansubs/vcf2pop/issues/1). Additionally, the VCF2PopTree developer reported that the software took 3.57 min (214 seconds) to analyze 100 genomes and 2 M variants (PMID: 31824783). Even so, VCF2Dis is approximately at least 10 times faster than VCF2PopTree. Due to these limitations, including the instability, slower performance and failure in running the tests of VCF2PopTree, we decided not to include it in the direct performance comparison. Instead, we discussed its advantages and disadvantages in the revised manuscript, noting its user-friendliness for non-technical users to analyze a small dataset.
Both #reviewer 1 and #reviewer 2 suggested that the efficiency of VCF2Dis might be attributed to the use of the C++ language. As pointed by #reviewer1, we should focus on the performance of distance calculation step because VCF2Dis aims to calculate p-distance. We agreed that the advantage of C++ language is one aspect. We found another C++ tool, ngsDist (https://github.com/fgvieira/ngsDist; https://doi.org/10.1111/bij.12511), which calculate only p-distance. Our tests showed that ngsDist consumes significantly more memory and runs much slower than VCF2Dis (updated Figure 2 in the revised manuscript). This highlights that the efficiency of VCF2Dis is not solely due to the programming language but also its optimized implementation. Additional details have been provided in the Methods section and additional file 1 of the revised manuscript.
3) Considering the importance of reconstructing population phylogeny from large-scale genotype data, the superior performance of VCF2Dis compared to existing genetic distance tools (e.g., VCF2PopTree, fastreeR, ngsDist), and its extensive application in scientific research (with over 190 citations according to Google Scholar, e.g., Nature (wheat, 2024, PMID: 38885696), Nature Genetics (watermelon, 2024, PMID: 38977857), Science Advance (honey bee, 2023, PMID: 37134176; human, 2024, PMID: PMID: 38579006)

) underscores its value in the field. We have revised the manuscript to better emphasize the advantages of VCF2Dis in this context.

We hope that with the amendments made in response to the reviewers' comments, the manuscript is now acceptable for publication in GigaScience.

Figure R1. the snapshots of running VCF2PopTree across a dataset of 91 samples with 1 million, 2 million and 3 million variants on a Windows 10 computer. (Note: the figure was shown in the coverLetter_Response file which was uploaded as an attached supplementary material)

Response (blue texts) to comments (black italic) point by point:

Reviewer #1:
comment: In this manuscript, the authors present a new tool, VCF2Dis, which facilitates the construction of phylogenetic trees from large datasets typically presented in VCF format. VCF2Dis provides a fast implementation for calculating pairwise distances and employs the ape tool to construct phylogenetic trees using either the neighbour-joining or UPGMA algorithms. It features an easy-to-use interface that does not require programming skills from the user. The authors compare the performance of VCF2Dis with a tool called fastreeR and demonstrate its superiority in terms of memory efficiency and speed.
Overall, while I find the concept of the manuscript interesting, I believe the introduction lacks sufficient motivation and background, and the comparison with other tools could be more detailed and accurate. Furthermore, the comparison is made against fastreeR, which is not published and does not have a preprint. The novelty of VCF2Dis appears to lie in its efficient implementation of distance evaluation, as it relies on the external tool ape for tree construction. I think this aspect is not adequately emphasized in the manuscript.
I have three major comments and several minor comments that I believe will help improve the manuscript.

Response: Thank you for your professional comments and suggestions, which have been invaluable in improving both our manuscript and the tool. We have thoroughly revised the manuscript in accordance with your professional and constructive suggestions. Below, we have provided detailed responses to each of your major and minor comments.

Major comments:
comment: 1) Lack of detailed introduction to existing phylogenetic tools. The manuscript does not provide sufficient background on the broader class of phylogenetic methods. VCF2Dis focuses on distance-based methods for phylogenetic tree construction, such as neighbour-joining and UPGMA, which are computationally efficient and use evaluated pairwise distances between samples to build trees. These methods are well-suited for working with large datasets, especially those provided in VCF format, and do not require sequence alignment.
In contrast, other groups of phylogenetic tools, such as RAxML, IQ-TREE, PhyML, and FastTree, use maximum likelihood estimation. These tools rely on substitution models to infer phylogenies, and they require alignment data as input. This class of methods is more complex and provides more accurate evolutionary inferences, but is generally more computationally intensive. They can handle large sample counts, but are restricted by gene-level analyses.
I believe it is essential for the manuscript to provide a broader context and highlight the distinctions between distance-based methods, like those implemented in VCF2Dis, and maximum likelihood methods, as they serve different purposes in phylogenetic analysis. It would also be helpful to explain why many tools use sequence-aligned data instead of VCF format—whether due to traditional practices, methodological requirements, or other reasons.

Response: Thank you for your thoughtful suggestions and kind comments. We apologize for any confusion caused by the unclear statements in the original manuscript. In response to your feedback, we have revised the introduction section to include a more detailed explanation of distance-based methods and maximum likelihood methods, highlighting their distinctions and respective applications in phylogenetic analysis. (lines 52-65)

comment: 2) Comparison with other tools. The manuscript compares VCF2Dis only with fastreeR, an unpublished tool, which makes it difficult to fully assess its strengths and weaknesses. I would expect the authors to compare VCF2Dis with other popular tools, especially VCF2PopTree, which they mention can handle up to 1,500 samples. While the authors mention that VCF2PopTree crashes on large datasets, I believe it should still be included in their comparative analysis to determine the extent to which it can be used effectively.
Due to VCF2Dis's feature for fast distance evaluation, I believe the comparison should focus solely on the distance conversion aspect (see my next comment). I suggest comparing it with PLINK's Hamming distance conversion, though I believe other methods also exist. However, this comparison must be conducted carefully—ideally by comparing conversions to the same distance types or to those with equivalent computational complexity.

Response: Thank you for your thoughtful suggestions and kind comments. FastreeR and VCF2PopTree are current other two tools that generated distance and construct population phylogeny directly from VCF files. FastreeR is indeed an unpublished tool, but it has been applied over 10 studies (https://scholar.google.com/scholar?q=fastreeR, e.g., PMID: 39215560, Mol Ecol, 2024; PMID: 39069530, Commun Biol,2024; PMID: 39443340, Planta,2024; PMID: 39461976, Sci Rep, 2024). Given its usage in the literature, we believe it is reasonable to compare VCF2Dis with FastreeR.

Regarding VCF2PopTree, although its original code limits the number of samples to less than 1500 (We also tested VCF2PopTree and found that it successfully processed a dataset containing 1,500 samples with 500 variants but failed when attempting to process a dataset with 1,501 samples and 500 variants), the performance tests presented in original publication were conducted on the test datasets with fewer than 100 samples and less than 4M variants (https://pmc.ncbi.nlm.nih.gov/articles/PMC6901002/). We conducted tests on a windows 10 computer (256GB memory, 24 threads, i9 10920x CPU) using a small dataset consisting of 91 samples with 1M, 2M and 3M variants. The source code of VCF2PopTree was slightly modified to include a function for displaying the runtime of the distance calculation step. The runtime for the 1M and 2M variants were 456 s and 596 s respectively. However, VCF2PopTree failed to respond when analyzing the 3M variants, even after 24 hours (a screenshot has been shown in the figure listed below, the figure was shown in the coverLetter_Response file which was uploaded as a attached supplementary material). Similar tests on a Mac M1 max computer yielded a runtime of 111 seconds for the 1M dataset, while the software failed to process the 2M and 3M variants. A similar issue has also been reported by a VCF2PopTree user (https://github.com/sansubs/vcf2pop/issues/1). Additionally, the VCF2PopTree developer reported that the software took 3.57 min (214 seconds) to analyze 100 genomes and 2 M variants (PMID: 31824783). Even so, VCF2Dis is approximately 10 times faster than VCF2PopTree. Due to these limitations, including the instability, slower performance and failure in running the tests of VCF2PopTree, we decided not to include it in the direct performance comparison. Instead, we discussed its advantages and disadvantages in the revised manuscript, noting its user-friendliness for non-technical users to analyze a small dataset. (lines 259-263)

We agreed with your suggestion to focus on distance comparison, as this is a key feature of VCF2Dis. As noted in the original paper of VCF2PopTree, other tools, PLINK and ngsDist can also calculate pairwise genetic distance. PLINK is capable of calculating Hamming distances but requires VCF files to be converted into its own format, which demands substantial memory resources (e.g., 257 GB for analyzing 78M variants across 2,504 human genomes). Additionally, PLINK employs dynamic multi-threading, utilizing most available threads, making direct performance comparisons with other tools less fair. The ngsDist (https://github.com/fgvieira/ngsDist; https://doi.org/10.1111/bij.12511), developed using C++ lanuage, on the other hand, cannot directly handle VCF files and requires external tools (e.g., bcftools → PLINK) to convert the file formats (VCF → BEAGLE). We have included ngsDist in our comparison, and as you suggested, we have updated the performance comparison to focus specifically on the distance calculation step. Our results show that VCF2Dis outperforms the other tools in this regard (updated Figure2 in the manuscript). We have revised the manuscript accordingly, with changes in red text. (e.g., lines 170-200)

Figure R1. the snapshots of running VCF2PopTree across a dataset of 91 samples with 1 million, 2 million and 3 million variants on a Windows 10 computer. (Note: the figure was shown in the coverLetter_Response file which was uploaded as an attached supplementary material)

Figure R2. The snapshot of a similar issue reported in the VCF2PopTree github. (https://github.com/sansubs/vcf2pop/issues/1)(Note: the figure was shown in the coverLetter_Response file which was uploaded as an attached supplementary material)

comment: 3) Comparison procedure. VCF2Dis consists of two components: distance evaluation and tree construction. The first part is novel, with the authors introducing an efficient implementation. The second part relies on the ape software for tree construction. The comparison presented in the manuscript evaluates VCF2Dis and fastreeR in terms of memory usage and speed. Unfortunately, due to the lack of a publication for fastreeR, I was unable to determine the method of tree construction used in that tool. If fastreeR also employs a method from ape, then the comparison is valid. However, if it does not, I believe the manuscript would benefit from conducting two separate comparisons: one for the distance evaluation methods and another for the tree construction methods. It is possible that the difference in efficiency may not stem from the distance evaluation that is a key feature for VCF2Dis, but rather from the fast tree construction in ape.

Response: Thank you for your thoughtful suggestions and kind comments. We have outputted the distance result generated by fastreeR and constructed the population phylogeny using ape package. The tree result is the consistent with the tree generated by VCF2Dis (ape). We also checked the distance method (cosine type distance) defined by the fastreeR author: (1-cosine_similarity)/2 where cosine_similarity could be one of -1(completely opposite samples), 0 (perpendicular samples), and 1 (identifical samples) and it only consider Biallelic or multiallelic (maximum 7 alternate alleles) SNP and/or INDEL variants (README in https://github.com/gkanogiannis/fastreeR). Therefore, for the Biallelic SNPs/INDELs, the "cosine type distance" is the same with p-distance and several branch discordances in population phylogeny generated by fastreeR and VCF2Dis is indeed led by different tree reconstructed software. We have corrected and clarified in the revised manuscript. (lines 202-204, 214-218)

Minor Comments:
comment: 1) The ape software used by VCF2Dis for phylogenetic analysis is mentioned at the end of the manuscript in the Methods section. I believe it should be introduced earlier, as its late mention may confuse readers and give the impression that VCF2Dis has its own implementation of phylogenetic tree construction.

Response: Thank you for your thoughtful suggestions and comments. As mentioned in the abstract and introduction, the population phylogeny step, based on the p-distance generated by VCF2Dis, utilizes the ape package. We appreciate your attention to this detail and thank you again for pointing it out. (e.g., lines 30,98, 114)

comment: 2) It would be helpful to provide a definition or at least a brief description of p-distance at the beginning of the manuscript.

Response: Thank you for your kind suggestions and comments. We have added a definition for the p-distance at the beginning of the manuscript in the revised version. (lines 91-92)
The p-distance refers to the proportion (p) of nucleotide sites at which two sequences differ. This metric is well-defined and thoroughly explained in the widely used MEGA software (https://www.megasoftware.net/mega1_manual/Distance.html).

comment: 3) It is unclear how the standard evaluation of p-distance is implemented in VCF2Dis. The manuscript would benefit from a figure illustrating the performance or a pseudocode listing of the implementation. I find the section "Accelerated Method of VCF2Dis" to be very brief.

Response: Thank you for your valuable feedback. We understand the need for further clarity regarding the standard evaluation of p-distance in VCF2Dis. The p-distance is well-defined and thoroughly explained in the widely used MEGA software (https://www.megasoftware.net/mega1_manual/Distance.html). We have added a description and citation in the appropriate location. (e.g., lines 92, 309)

We have provided a more detailed description of the implementation of p-distance in the revised manuscript. (lines 308-328).

Regarding the section “Accelerated Method of VCF2Dis,” we agree that it could benefit from more detailed explanation. In the revised manuscript, we have expanded this section to better describe the optimizations and techniques used to accelerate the computation, providing readers with a clearer understanding of the improvements made. (lines 334-348).

In addition, we have provided a pseudo-code to illustrate the implementation of p-distance calculation and to highlight the improvements in performance (running time and memory usage, Supplementary Note 1 in Additional file 1).

We appreciate your constructive suggestions, which will help improve the manuscript’s clarity and completeness.

comment: 4) To demonstrate that the proposed implementation is both correct and efficient, it would be beneficial to compare VCF2Dis with a naive implementation of p-distance evaluation, implemented in C++.

Response: Thank you for your thoughtful comments. To demonstrate the accuracy of VCF2Dis, it has been widely used in various research studies, with over 190 citations (as of Google Scholar search on December 20, 2024), including publications in high-impact journals such as Nature (PMID: 38885696), Nature Genetic (PMID: 38977857), Nature Communications (PMID: 37069152) and Science Advances (PMID: 38579006). These citations suggest the tool’s robustness and accuracy.

Furthermore, we performed a detailed comparison of the distance results generated by VCF2Dis, fastreeR using a small dataset containing biallelic SNPs without missing sites (Figure R3 listed below). The results were identical, although the distance metric defined by fastreeR (cosine type distance) differs. This confirms the correctness and consistency of VCF2Dis.

Regarding performance, we agreed that the advantage of C++ language is one aspect. VCF2Dis was originally developed in C++ and continues to be optimized. Significant improvements were made in versions 1.36, 1.44, and 1.53, with runtime reductions from 4 hours (version 1.36) to 1 hour (version 1.44), and further down to 50 minutes (version 1.53) for the same test dataset. Additionally, we found another C++ tool, ngsDist (https://github.com/fgvieira/ngsDist; https://doi.org/10.1111/bij.12511), which calculate only p-distance. Our tests showed that ngsDist consumes significantly more memory and runs much slower than VCF2Dis (updated Figure 2 in the revised manuscript). This highlights that the efficiency of VCF2Dis is not solely due to the programming language but also its optimized implementation. Additional details have been provided in the Methods section of the revised manuscript and Supplementary Note 1 in Additional file 1. (lines 329-348)

Figure R3. The snapshot includes the runtime performance of fastreeR and VCF2Dis, alongside their corresponding distance matrices, highlighting the computational differences and distance consistency between the two tools. (Note: the figure was shown in the coverLetter_Response file which was uploaded as an attached supplementary material)

comment: 4) I suggest including some final results in the abstract and introduction. These could be part of the results, but they should be specific and impressive. For example, "VCF2Dis requires only 0.37 GB of memory when analyzing 2,504 individuals with 81.2 million variants," or "VCF2Dis performs the analysis of 81.2 million variants across 203 individuals in just about 3 hours and is N times faster than fastreeR."

Response: Thank you for your professional comments. We have added this result in the abstract and introduction. (lines 32-33, 93-96)

comment: 5) The authors introduced a multithreaded version of VCF2Dis and demonstrated that it is 22 times faster when using 60 threads. However, it is unclear how the tool scales with the number of threads. The manuscript indicates that the speed-up is not linear; the speed is only 22 times faster, not 60 times. I suggest the authors provide benchmarking results for VCF2Dis to illustrate its performance with different

thread counts.

Response: Thank you for your professional comments. We are sorry for the confusion caused by our unclear statement.

“It seldom occurs that get an N times speedup when running a program parallelized using OpenMP on a N processor platform”. The performance of OpenMP is limited by factors including:
1) When a dependency exists, a process must wait until the data it depends on it computed;
2) When multiple processes share a non-parallel proof resource (like a file to write in), therefore, each thread must wait until the other thread releases the resource;
3) A large part of the program may not be parallelized by OpenMP, which means that the theoretical upper limit of speedup is limited according to Amdahl’s law.
4) Many other common problems affecting the final speedup in parallel computing also apply to OpenMP, like load balancing and synchronization overhead.
Above was copied from the wiki (https://en.wikipedia.org/wiki/OpenMP)

For multi-threaded VCF2Dis, the VCF input file is divided into multiple blocks, which are processed sequentially and calculated in parallel across available threads (Figure R4 of the response below, the figure was shown in the coverLetter_Response file which was uploaded as a attached supplementary material). The OpenMP library is utilized to accelerate the loop calculations within each block (as shown on line 583 in Figure R5 of the response below, the figure was shown in the coverLetter_Response file which was uploaded as an attached supplementary material).

The non-linear speedup using the multi-threaded VCF2Dis (VCF2Dis_multi) may be caused by following reasons:
First, not all steps (e.g., read and write) are separately parallelized. Second, our test of multi-threaded VCF2Dis was performed on a node computer with 64 threads, not all 60 working threads are running at all times during execution, as the CPU usage is dynamic. Third, although 60 threads are employed, the threads often wait for each other during computation, preventing the CPU utilization from reaching 100%. Additionally, the overhead from thread switching consumes a significant amount of CPU resources.

In the original version of VCF2Dis_multi, we implemented a dynamic parallel strategy that utilized all available CPU cores. In the updated version, we switched to a static parallel strategy, allowing users to specify the number of threads. As per your suggestion, we have included benchmarking results to demonstrate the performance of VCF2Dis_multi with varying thread counts. These tests were conducted on a dataset containing 2,504 samples with 1 million variants (see Figure R6 below and Fig. S1 in Additional File 1, the figure was shown in the coverLetter_Response file which was uploaded as an attached supplementary material). We have revised the manuscript accordingly. (lines 147-165, 235-241)

Figure R4. The illustration for multi-threaded VCF2Dis. (Note: the figure was shown in the coverLetter_Response file which was uploaded as an attached supplementary material)

Figure R5. The source code of VCF2Dis for parallel acceleration. (Note: the figure was shown in the coverLetter_Response file which was uploaded as an attached supplementary material)

Figure R6. The performance of multi-threaded VCF2Dis (VCF2Dis_multi) with different thread counts. The tests were performed on single-threaded mode VCF2Dis and multi-threaded VCF2Dis (threads=2,4,8,16,32). The left y-axis indicates the runtime and the right y-axis indicate thread efficiency. The fold of speedup was shown in yellow shaded texts. The thread efficiency was calculated as the ratio of speedup to the number of threads. (Note: this figure was also shown in Fig. S1 in additional file 1; the figure was shown in the coverLetter_Response file which was uploaded as an attached supplementary material)

comment: 6) It is stated that, in terms of runtime performance, VCF2Dis and fastreeR exhibited

exponential increases. How was this determined? Does it scale exponentially with the number of samples? Could the authors provide an explanation of that complexity? I would expect the complexity to derive from both the distance evaluation and tree construction processes. The distance complexity is O(n^2 M), where nnn is the number of samples and MMM is the number of variants (which also changes with the number of samples). The tree construction complexity should take O(n^3). Therefore, I expect the overall complexity to be O(n^2 M) +O n^3). Could the authors elaborate on this?

Response: Thank you for your professional comments and suggestions. We are sorry for the confusion caused by unclear statement. As you pointed correctly, the distance complexity is O(n^2 M) and the tree construction complexity is O(n^3) and the overall complexity is O(n^ M) + O(n^3). However, given the fact that M (the number of variants, 10^6) is often much bigger than N (the number of samples, 10^2~10^3), thus the total runtime is mainly determined by the step of distance calculation. Simply, time1 = O(n^2 M), time2 = O(n^3), and time2/time1 <0.01, namely, the runtime of tree construction only occupy less than 1% of the overall runtime. We have clarification this in the revised manuscript. (lines 350-360)
To compare the runtime affected by the number of samples and variants, we tested the runtime by fixing the number of variants and increased the number of samples (e.g., 100, 200, 400, 500, 800,1000), or fixing the number of samples and increased the number of variants (1M, 2M, 3M,…,10M). (the performance was shown in Fig.2 of the main text, the test datasets and scripts have been uploaded the giga FTP). We have clarified in the revised manuscript (lines 362-377).

comment: 7) Line 142: "In contrast, fastreeR grouped some 143 individuals clustered with population from different regions.". If fastreeR employs a different method for building the phylogenetic tree (as noted in Major Comment #3) than ape, then it is not surprising that the resulting tree could differ and potentially be less accurate. However, this observation does not provide any insight into the distance metric used. From the experiments, it is not possible to determine whether the discrepancies arise from the distance metrics (p-distance for VCF2Dis and cosine distance for fastreeR) or from the phylogenetic tree construction method. The authors should be clear and accurate in their conclusions.

Response: Thank you for your kind points and professional comments. We are sorry for the confusions caused by unclear statement and not thoroughly considered. We have carefully checked the intermediate distance output and found it was the same as we calculated.
The cosine type distance defined is calculated (1-cosine_similarity)/2, where cosine_similarity could be one of -1(completely opposite samples), 0 (perpendicular samples), and 1 (identifical samples) and it only consider Biallelic or multiallelic (maximum 7 alternate alleles) SNP and/or INDEL variants (README in https://github.com/gkanogiannis/fastreeR). Therefore, for the Biallelic SNPs/INDELs, the "cosine type distance" is the same with p-distance and several branch discordances in population phylogeny generated by fastreeR and VCF2Dis is indeed led by different tree reconstructed software. We have clarified in the revised manuscript (lines 214-218). Thank you for your professional comments.

Reviewer #2:
comment: the authors claim that they have developed an efficient tool to calculate pairwise genetic distance. Also, the tool after processing the pairwise distance, uses phylogenetic inference algorithms very know to produce the tree.
In my opinion, it is not clear on this work what are the algorithmic details of the authors approach that turns their approach more efficient, since, for instance the authors do not discuss a pseudo-code of their approach. Also, what is the asymptotical complexity of the approach? Did the authors did a formal study about that?

Response: Thank you for your kind points and professional comments. We are sorry for the confusions caused by unclear statement. We agreed with your suggestion that it is required the algorithmic details for improving efficiency of VCF2Dis. We have added the details in the revised manuscript. (lines 334-349)
For memory efficiency, we employed a strategy by reading and processing in a manner of line-by-line, ensuring that memory usage is affected only by the number of samples and remains independent of the number of variants. For runtime efficiency, the distance complexity is O(n^2 M) and the tree construction complexity is O(n^3) and the overall complexity is O(n^ M) + O(n^3). However, given the fact that M (the number of variants, 10^6) is often much bigger than N (the number of samples, 10^2~10^3), thus the

total runtime is mainly determined by the step of distance calculation. Simply, time1 = O(n^2 M), time2 = O(n^3), and time2/time1 <0.01, namely, the runtime of tree construction only occupy less than 1% of the overall runtime. We have added the details in the revised manuscript (lines 350-360). The pseudo-code of VCF2Dis has been illustrated and added in the supplementary materials (Supplemental Note 1 in Additional file 1).

comment: Moreover, it does not seem a fair comparison to the other tools/approaches since they are written in different languages, executing in different execution environments. It should be important to understand the asymptotical complexity of the different approaches and the experimental evaluation should discuss the diferences that result from the execution environment.

Response: Thank you for your kind points and professional comments. We are sorry for the confusions caused by unclear statement.
We agree to some extent that that development languages may lead to performance difference. VCF2Dis was originally developed in C++ and continues to be optimized. Significant improvements were made in versions 1.36, 1.44, and 1.53, with runtime reductions from 4 hours (version 1.36) to 1 hour (version 1.44), and further down to 50 minutes (version 1.53) for the same test dataset. According to the comment and suggestions from #reviewer1, that we should focus on the comparison performance in the step of distance calculation. We found another C++ tool, ngsDist (https://github.com/fgvieira/ngsDist; https://doi.org/10.1111/bij.12511), which calculate only p-distance. Our tests showed that ngsDist consumes significantly more memory and runs much slower than VCF2Dis (updated Figure 2 in the revised manuscript). This highlights that the efficiency of VCF2Dis is not solely due to the programming language but also its optimized implementation. Additional details have been provided in the Methods section of the revised manuscript. (lines 362-377)

comment: With respect to the repository, and taking into account that the tool should be executed by non computer science experts, the authors should provide an executable that allow to test the tool.

Response: Thank you for your kind comments for the repository, the readily executable version of VCF2Dis has been packaged in Docker and Singularity containers, which are available on GitHub (https://github.com/hewm2008/VCF2Dis). Users can easily pull the images and run VCF2Dis without the need for compilation and installation. Detailed instructions are provided in the GitHub repository. (lines 274-277)

[Reviewer #3](Additional technical advice)

comment: A number of software tools have been developed in the past to perform population genetic and evolutionary analysis using large-scale genomic data in Variant Call Format (VCF). The present study has developed another software program, 'VCF2Dis,' which receives a VCF file, computes pairwise genetic distance and infers population phylogeny using simple methods such as Neighbour-Joining and UPGMA methods. While this program has some small incremental benefits over the other available software, such as VCF2POPTREE and fastreeR, it does have limitations that the other software don't. Therefore, I don't think this manuscript has merits that warrant publication in GigaScience, but it may be suitable for other specialized journals.

Response: Thank you for your comments. We have clearly stated the limitation of VCF2Dis in the Discussion section: “However, some limitations should also be considered in the future work, …, Thirdly, future developments of VCF2Dis could also address user needs for more interactive features, such as a graphical user interface (GUI), which would lower the entry barrier for non-technical users.”. (lines 249-259)
We agree that user-friendly tools like VCF2PopTree enable non-computer science experts to perform pairwise distance calculations and generate NJ trees easily. We conducted tests on a windows 10 computer (256GB memory, 24 threads, i9 10920x CPU) using a small dataset consisting of 91 samples with 1M, 2M and 3M variants. The source code of VCF2PopTree was slightly modified to include a function for displaying the runtime of the distance calculation step. The runtime for the 1M and 2M variants were 456 s and 596 s respectively. However, VCF2PopTree failed to respond when analyzing the 3M variants, even after 24 hours (a screenshot has been shown in the figure listed below). Similar tests on a Mac M1 max computer yielded

a runtime of 111 seconds for the 1M dataset, while the software failed to process the 2M and 3M variants. A similar issue has also been reported by a VCF2PopTree user (https://github.com/sansubs/vcf2pop/issues/1). Additionally, the VCF2PopTree developer reported that the software took 3.57 min (214 seconds) to analyze 100 genomes and 2 M variants (PMID: 31824783). Even so, VCF2Dis is approximately 10 times faster than VCF2PopTree. We have discussed its advantages and disadvantages in the revised manuscript, noting its user-friendliness for non-technical users to analyze small dataset. (lines 259-263)

According to the suggestion from #reviewer 1, we also took another p-distance calculation tool, ngsDist developed using C++ language, for performance comparison. Compared to other two tools, fastreeR and ngsDist, VCF2Dis demonstrates superior performance in both memory efficiency and running speed (Figure 2 in the revised manuscript). Considering the importance of reconstructing distance-based population phylogeny from large-scale genotype data, the superior performance of VCF2Dis compared to existing p-distance tools (e.g., VCF2PopTree, fastreeR, ngsDist), and its extensive application in scientific research (with over 190 citations according to Google Scholar, e.g., Nature Genetic (PMID: 38977857), Nature Communications (PMID: 37069152) and Science Advances (PMID: 38579006)) underscores its value in the field.

To facilitate further evaluation, we have made all test datasets available on giga FTP server, and we welcome you to verify the results.

comment: The authors highlight the positive features of VCF2Dis, such as faster execution time compared to VCF2POPTREE and fastreeR. They failed to mention the limitations in comparison with the same software. For instance, VCF2POPTREE is a client-side browser program (no need for installation), and hence, it is easy to draw a phylogeny in a couple of clicks. On the other hand, VCF2Dis is a command line-based program, which has to be complied, installed, and run through a terminal that are not user-friendly, particularly for those who have limited computational skills. Hence, I suggest the authors list the limitations of their software as well. This will inform the users to choose the right software tool that serves their needs.

Response: Thank you for your kind comments and suggestions. We acknowledge that VCF2PopTree is indeed a user-friendly tool. In response, we plan to develop a graphical user interface (GUI) for VCF2Dis to make it more accessible and lower the entry barrier for non-technical users.
We have clearly stated the limitation of VCF2Dis in the Discussion section (lines 249-259). To avoid the compiling and installation, we have made popular docker and singularity containers of VCF2Dis deposited in the github (https://github.com/hewm2008/VCF2Dis/releases/tag/v1.53).(lines 274-277)

comment: Lines 57-58 states that VCF2POPTREE can analyze only <1500 genomes. Lines 120-121 say that fastreeR also crashes when the number of genomes (or samples) is >1000. However, the performance of VCF2Dis was compared only with that of fastreeR but not with that of VCF2POPTREE using 1000 genomes. The reason for this was stated as VCF2POPTREE crashes when the number of genomes was large (lines 116-117). This statement was contradictory to that mentioned in lines 57-58, which means that VCF2POPTREE is capable of analysing 1000 genomes. I suggest that the authors compare the performances of both VCF2POPTREE and fastreeR with VCF2Dis using 1000 or less genomes.

Response: Thank you for your comments. We apologize for the unclear statements and any inappropriate descriptions. We have revised the relevant statements in the revised manuscript.
For fastreeR, it was unable to complete the calculations within a reasonable timeframe when processing datasets with more than 1,000 samples.
For VCF2PopTeee, it is not contradictory for the statement. The limitations of 1500 samples were inferred from the source code of VCF2PopTree (e.g., var all=new Array(1500);). We also tested VCF2PopTree and found that it successfully processed a dataset containing 1,500 samples with 500 variants but failed when attempting to process a dataset with 1,501 samples and 500 variants. In the original publication of VCF2PopTree, the performance tests presented were conducted on the test datasets with fewer than 100 samples and less than 4M variants (https://pmc.ncbi.nlm.nih.gov/articles/PMC6901002/). We conducted tests on a windows 10 computer (256GB memory, 24 threads, i9 10920x CPU) using a small dataset consisting of 91 samples with 1M, 2M and 3M variants. The source code of VCF2PopTree was slightly

modified to include a function for displaying the runtime of the distance calculation step. The runtime for the 1M and 2M variants were 456 s and 596 s respectively. However, VCF2PopTree failed to respond when analyzing the 3M variants, even after 24 hours (a screenshot has been shown in the figure listed below). Similar tests on a Mac M1 max computer yielded a runtime of 111 seconds for the 1M dataset, while the software failed to process the 2M and 3M variants. A similar issue has also been reported by a VCF2PopTree user (https://github.com/sansubs/vcf2pop/issues/1). Additionally, the VCF2PopTree developer reported that the software took 3.57 min (214 seconds) to analyze 100 genomes and 2 M variants (PMID: 31824783). Even so, VCF2Dis is approximately 10 times faster than VCF2PopTree. Due to these limitations, including the instability, slower performance and failure in running the tests of VCF2PopTree, we decided not to include it in the direct performance comparison. Instead, we discussed its advantages and disadvantages in the revised manuscript, noting its user-friendliness for non-technical users to analyze a small dataset.

Figure R1. the snapshots of running VCF2PopTree across a dataset of 91 samples with 1 million, 2 million and 3 million variants on a Windows 10 computer. (Note: the figure was shown in the coverLetter_Response file which was uploaded as an attached supplementary material)
