# Supplementary material for: VCF2Dis: an ultra-fast and efficient tool to calculate pairwise genetic distance and construct population phylogeny from VCF files
Source: Gigascience. 2025 Apr 4;14:giaf032. doi: 10.1093/gigascience/giaf032 (PMC11970368; doi:10.1093/gigascience/giaf032)
Supplement: giaf032_Response_to_Reviewer_Comments_Revision_1 [file giaf032_response_to_reviewer_comments_revision_1.pdf]

Author's Response To Reviewer Comments

4 Feb, 2025
To
The Editor,
GigaScience

Dear Editor,
Thank you for carefully reviewing our manuscript, “VCF2Dis: an ultra-fast and efficient tool to calculate pairwise genetic distance and construct population phylogeny from VCF files” (GIGA-D-24-00393R1).

First, we appreciate for giving an opportunity to submit our revised manuscript, and also your kindly positive comments and critical suggestions, as well as two reviewers’ comments, which have helped us to greatly improved our submitted manuscript as well as our software, VCF2Dis.

We have carefully read the referees’ comments. We would like to express our sincere thanks to the reviewers for their constructive and positive comments. We have addressed all their suggestions, and the manuscript has been edited accordingly, with changes highlighted in red text.

The major amendments were listed below:
1. We have revised and resubmitted the manuscript as the “Technical Note” section.
2. The results and discussion on the population phylogeny comparison generated by fastreeR and VCF2Dis have been removed in response to reviewer #1’s comments.
3. A concise pseudocode for the p-distance calculation and accelerated methods in VCF2Dis has been added to the manuscript in response to reviewer #2’s comments.

We hope that with the amendments made in response to the reviewers’ comments, the manuscript is now acceptable for publication in GigaScience.

Sincerely,
Dr. Lian Xu
(On behalf of the authors)
Institute for translational neuroscience, the Second Affiliated Hospital of Nantong University, Nantong University, Nantong, Jiangsu, 226001, China.
Email: xulian@ntu.edu.cn

Response to comments point by point:

Ekaterina Noskova (Reviewer 1)
1. Reviewer #1: The authors have carefully addressed my previous suggestions and comments, resulting in a much clearer and improved manuscript. This has allowed me to form a more comprehensive opinion about both the manuscript and the tool, VCF2Dis, presented within it. The authors effectively highlight the problem of evaluating distance matrices for large datasets and provide evidence of the limitations in existing tools. Their benchmarking results demonstrate that VCF2Dis is indeed significantly faster than comparable tools.

Response: We sincerely appreciate your thoughtful feedback and positive evaluation of our revised manuscript and VCF2Dis.

2. Despite these improvements, I believe the manuscript lacks a substantial scientific contribution and would not recommend it for publication in GigaScience as a full research article. However, it may be better

suited for a shorter format, such as a technical note, similar to how the tool VCF2PopTree was published.

Response: Thank you for your professional suggestions. We did not shorten the paper, as there is no strict length restriction for Technical Notes. We have resubmitted the manuscript as a “Technical Note.”

3. In response to my comments, the authors provided detailed explanations of VCF2Dis's implementation, which contributes to its efficiency. These include line-by-line reading of VCF files, pointer-based string operations, eliminating redundant computations using upper triangle matrix evaluations only, and parallelization using OpenMP. While these features contribute to the tool's performance, I find these approaches to be straightforward and relatively standard methods for handling large datasets in C++, rather than technically innovative or advanced.

Response: We greatly appreciate your insightful feedback and your recognition of the key features contributing to VCF2Dis’s efficiency. While we agree that methods such as line-by-line reading, pointer-based string operations, and parallelization using OpenMP are common strategies for handling large datasets, our intention was to highlight how their combined implementation in VCF2Dis leads to a significant performance improvement.
In line with your suggestion, we have resubmitted the manuscript as a “Technical Note.”

4. The manuscript includes an experiment using a real dataset from the 1000 Genomes Project, where VCF2Dis is compared with fastreeR. The results demonstrate that VCF2Dis produces a more accurate phylogenetic tree. Upon further investigation, the authors determined that the difference arises from the methods used for phylogenetic tree construction, as the distance matrices generated by both tools were identical. I believe this section may be confusing for the reader and requires revision. The main feature of VCF2Dis is its ability to evaluate distance matrices quickly, and I would therefore expect a direct comparison of this step between the two tools. The observed differences in phylogenetic trees and the more accurate results obtained using VCF2Dis could instead be presented as supporting evidence for why the ape package was chosen as the phylogenetic method in VCF2Dis. However, I would argue that a single case is not sufficient to demonstrate the superiority of ape over the method used in fastreeR. Additional experiments and comparisons are necessary to substantiate this claim.

Response: We greatly appreciate your valuable feedback and professional comments. We agree that a single case is not sufficient to conclusively demonstrate the superiority of the ape method over that used in fastreeR. In response to your suggestion, we have removed the results and discussion comparing the population phylogeny generated by VCF2Dis+ape and fastreeR.

5. That said, the manuscript serves as a solid technical paper. It identifies a genuine problem—computing distance matrices for large datasets—presents an efficient solution, and provides a fair comparison with existing tools to demonstrate its superiority. While its current contribution may not be sufficient for a full research article, it could still hold value as a Technical Note with some additional experiments or analyses to enhance its impact.

Response: Thank you for your professional suggestions. We have resubmitted the manuscript as a “Technical Note.”

Reviewer #2:
Catia Vaz (Reviewer 2)
1. It thank the authors for their answers, but I have still some doubts.

When the authors say:
"For memory efficiency, we employed a strategy by reading and processing in a manner of line-by-line, ensuring that memory usage is affected only by the number of samples and remains independent of the number of variants. For runtime efficiency, the distance complexity is O(n^2 M) ".
I would like to see more details in the paper (perhaps a pseudo-code) of how you, in runtime, depend on the number of variants but in memory you don't depend on it. You don't keep it in memory? If so, how you

process the distance depending on that? That should be clarified better in my opinion.
Also, when you refer:
" is not solely due to the programming language but also its optimized implementation."
So, what specific optimizations that you have and others don't allow to do this affirmation? Again, a pseudo code with some comments will help to see what is the innovation of your implementation.

Response: Thank you for your kind comments. The optimized implementation of VCF2Dis incorporates several features, including line-by-line reading of VCF files, pointer-based string operations, eliminating redundant computations through upper triangle matrix evaluations, and parallelization using OpenMP.
We have added a pseudo-code and a detailed explanation of the calculation of p-distance and the optimization process to the Supplementary Note 1. Additionally, as per your suggestion, we have now included a concise version of the pseudocode in the manuscript.

2. Can we reproduce your tests to experiment with other datasets, namely the comparison of your approach with the other one that you mention? I am sorry, I did not find that to reproduce on the github.

Response: Thank you for your kind comments. Due to the large size of the test data, we did not upload it to GitHub previously. However, we have provided all the scripts and test datasets used in this study on the FTP server hosted by GigaDB (the username and password are listed below) to allow others to reproduce the results. Additionally, we have now uploaded the test scripts for downloading and generating the test dataset, as well as the running scripts, to GitHub at https://github.com/hewm2008/VCF2Dis/tree/main/RunTest. Furthermore, this tool has been widely used in real datasets and has been cited more than 203 times (according to a Google Scholar search on February 3, 2025).

The username and password to access the test dataset and scripts to the ftp provided by GigaDB.
username = user270
password = SNBjQos8sIubL
FTP server = files.gigadb.org

3. I also thank the authors for making available a docker image to test it.

Response: Thank you for your professional suggestion again.
