# Supplementary material for: VCF2Dis: an ultra-fast and efficient tool to calculate pairwise genetic distance and construct population phylogeny from VCF files
Source: Gigascience. 2025 Apr 4;14:giaf032. doi: 10.1093/gigascience/giaf032 (PMC11970368; doi:10.1093/gigascience/giaf032)
Supplement: giaf032_Reviewer_1_Report_Original_Submission — Ekaterina Noskova -- 10/8/2024 [file giaf032_reviewer_1_report_original_submission.pdf]

Reviewer Report

Title: VCF2Dis: an ultra-fast and efficient tool to calculate pairwise genetic distance and construct population phylogeny from VCF files

Version: Original Submission **Date:** 10/8/2024

Reviewer name: Ekaterina Noskova

Reviewer Comments to Author:

In this manuscript, the authors present a new tool, VCF2Dis, which facilitates the construction of phylogenetic trees from large datasets typically presented in VCF format. VCF2Dis provides a fast implementation for calculating pairwise distances and employs the ape tool to construct phylogenetic trees using either the neighbour-joining or UPGMA algorithms. It features an easy-to-use interface that does not require programming skills from the user. The authors compare the performance of VCF2Dis with a tool called fastreeR and demonstrate its superiority in terms of memory efficiency and speed.Overall, while I find the concept of the manuscript interesting, I believe the introduction lacks sufficient motivation and background, and the comparison with other tools could be more detailed and accurate. Furthermore, the comparison is made against fastreeR, which is not published and does not have a preprint. The novelty of VCF2Dis appears to lie in its efficient implementation of distance evaluation, as it relies on the external tool ape for tree construction. I think this aspect is not adequately emphasized in the manuscript.I have three major comments and several minor comments that I believe will help improve the manuscript.Major comments:1) Lack of detailed introduction to existing phylogenetic tools. The manuscript does not provide sufficient background on the broader class of phylogenetic methods. VCF2Dis focuses on distance-based methods for phylogenetic tree construction, such as neighbour-joining and UPGMA, which are computationally efficient and use evaluated pairwise distances between samples to build trees. These methods are well-suited for working with large datasets, especially those provided in VCF format, and do not require sequence alignment.In contrast, other groups of phylogenetic tools, such as RAxML, IQ-TREE, PhyML, and FastTree, use maximum likelihood estimation. These tools rely on substitution models to infer phylogenies, and they require alignment data as input. This class of methods is more complex and provides more accurate evolutionary inferences, but is generally more computationally intensive. They can handle large sample counts, but are restricted by gene-level analyses.I believe it is essential for the manuscript to provide a broader context and highlight the distinctions between distance-based methods, like those implemented in VCF2Dis, and maximum likelihood methods, as they serve different purposes in phylogenetic analysis. It would also be helpful to explain why many tools use sequence-aligned data instead of VCF formatâ€”whether due to traditional practices, methodological requirements, or other reasons.2) Comparison with other tools. The manuscript compares VCF2Dis only with fastreeR, an unpublished tool, which makes it difficult to fully assess its strengths and weaknesses. I would expect the authors to compare VCF2Dis with other popular tools, especially VCF2PopTree, which they mention can handle up to 1,500 samples. While the authors mention that VCF2PopTree crashes on large datasets, I believe it should still be included in their comparative analysis to determine the extent to

which it can be used effectively.Due to VCF2Dis's feature for fast distance evaluation, I believe the comparison should focus solely on the distance conversion aspect (see my next comment). I suggest comparing it with PLINK's Hamming distance conversion, though I believe other methods also exist. However, this comparison must be conducted carefullyâ€"ideally by comparing conversions to the same distance types or to those with equivalent computational complexity.3) Comparison procedure. VCF2Dis consists of two components: distance evaluation and tree construction. The first part is novel, with the authors introducing an efficient implementation. The second part relies on the ape software for tree construction. The comparison presented in the manuscript evaluates VCF2Dis and fastreeR in terms of memory usage and speed. Unfortunately, due to the lack of a publication for fastreeR, I was unable to determine the method of tree construction used in that tool. If fastreeR also employs a method from ape, then the comparison is valid. However, if it does not, I believe the manuscript would benefit from conducting two separate comparisons: one for the distance evaluation methods and another for the tree construction methods. It is possible that the difference in efficiency may not stem from the distance evaluation that is a key feature for VCF2Dis, but rather from the fast tree construction in ape.Minor comments:1) The ape software used by VCF2Dis for phylogenetic analysis is mentioned at the end of the manuscript in the Methods section. I believe it should be introduced earlier, as its late mention may confuse readers and give the impression that VCF2Dis has its own implementation of phylogenetic tree construction.2) It would be helpful to provide a definition or at least a brief description of p-distance at the beginning of the manuscript.3) It is unclear how the standard evaluation of p-distance is implemented in VCF2Dis. The manuscript would benefit from a figure illustrating the performance or a pseudocode listing of the implementation. I find the section "Accelerated Method of VCF2Dis" to be very brief.4) To demonstrate that the proposed implementation is both correct and efficient, it would be beneficial to compare VCF2Dis with a naive implementation of p-distance evaluation, implemented in C++.4) I suggest including some final results in the abstract and introduction. These could be part of the results, but they should be specific and impressive. For example, "VCF2Dis requires only 0.37 GB of memory when analyzing 2,504 individuals with 81.2 million variants," or "VCF2Dis performs the analysis of 81.2 million variants across 203 individuals in just about 3 hours and is N times faster than fastreeR."5) The authors introduced a multithreaded version of VCF2Dis and demonstrated that it is 22 times faster when using 60 threads. However, it is unclear how the tool scales with the number of threads. The manuscript indicates that the speed-up is not linear; the speed is only 22 times faster, not 60 times. I suggest the authors provide benchmarking results for VCF2Dis to illustrate its performance with different thread counts.6) It is stated that, in terms of runtime performance, VCF2Dis and fastreeR exhibited exponential increases. How was this determined? Does it scale exponentially with the number of samples? Could the authors provide an explanation of that complexity? I would expect the complexity to derive from both the distance evaluation and tree construction processes. The distance complexity is O(n^2 M), where nnn is the number of samples and MMM is the number of variants (which also changes with the number of samples). The tree construction complexity should take O(n^3). Therefore, I expect the overall complexity to be O(n^2 M + n^3). Could the authors elaborate on this?7) Line 142: "In contrast, fastreeR grouped some 143 individuals clustered with population from different regions.". If fastreeR employs a different method for building the phylogenetic tree (as noted in Major Comment #3) than ape, then it is not surprising that the resulting tree could differ and potentially be less accurate. However, this observation does not provide any insight into the distance metric used. From the

experiments, it is not possible to determine whether the discrepancies arise from the distance metrics (p-distance for VCF2Dis and cosine distance for fastreeR) or from the phylogenetic tree construction method. The authors should be clear and accurate in their conclusions.

Level of Interest

Please indicate how interesting you found the manuscript: Choose an item.

Quality of Written English

Please indicate the quality of language in the manuscript: Choose an item.

Declaration of Competing Interests

Please complete a declaration of competing interests, considering the following questions:

- Have you in the past five years received reimbursements, fees, funding, or salary from an organisation that may in any way gain or lose financially from the publication of this manuscript, either now or in the future?
- Do you hold any stocks or shares in an organisation that may in any way gain or lose financially from the publication of this manuscript, either now or in the future?
- Do you hold or are you currently applying for any patents relating to the content of the manuscript?
- Have you received reimbursements, fees, funding, or salary from an organization that holds or has applied for patents relating to the content of the manuscript?
- Do you have any other financial competing interests?
- Do you have any non-financial competing interests in relation to this paper?

If you can answer no to all of the above, write 'I declare that I have no competing interests' below. If your reply is yes to any, please give details below.

I declare that I have no competing interests

I agree to the open peer review policy of the journal. I understand that my name will be included on my report to the authors and, if the manuscript is accepted for publication, my named report including any attachments I upload will be posted on the website along with the authors' responses. I agree for my report to be made available under an Open Access Creative Commons CC-BY license (http://creativecommons.org/licenses/by/4.0/). I understand that any comments which I do not wish to be included in my named report can be included as confidential comments to the editors, which will not be published.

Choose an item.

To further support our reviewers, we have joined with Publons, where you can gain additional credit to further highlight your hard work (see: https://publons.com/journal/530/gigascience). On publication of this paper, your review will be automatically added to Publons, you can then choose whether or not to claim your Publons credit. I understand this statement.

Yes Choose an item.
