# Supplementary material for: VCF2Dis: an ultra-fast and efficient tool to calculate pairwise genetic distance and construct population phylogeny from VCF files
Source: Gigascience. 2025 Apr 4;14:giaf032. doi: 10.1093/gigascience/giaf032 (PMC11970368; doi:10.1093/gigascience/giaf032)
Supplement: giaf032_Reviewer_1_Report_Revision_1 — Ekaterina Noskova -- 1/24/2025 [file giaf032_reviewer_1_report_revision_1.pdf]

Reviewer Report

Title: VCF2Dis: an ultra-fast and efficient tool to calculate pairwise genetic distance and construct population phylogeny from VCF files

Version: Revision 1 **Date:** 1/24/2025

Reviewer name: Ekaterina Noskova

Reviewer Comments to Author:

The authors have carefully addressed my previous suggestions and comments, resulting in a much clearer and improved manuscript. This has allowed me to form a more comprehensive opinion about both the manuscript and the tool, VCF2Dis, presented within it. The authors effectively highlight the problem of evaluating distance matrices for large datasets and provide evidence of the limitations in existing tools. Their benchmarking results demonstrate that VCF2Dis is indeed significantly faster than comparable tools.Despite these improvements, I believe the manuscript lacks a substantial scientific contribution and would not recommend it for publication in GigaScience as a full research article. However, it may be better suited for a shorter format, such as a technical note, similar to how the tool VCF2PopTree was published.In response to my comments, the authors provided detailed explanations of VCF2Dis's implementation, which contributes to its efficiency. These include line-by-line reading of VCF files, pointer-based string operations, eliminating redundant computations using upper triangle matrix evaluations only, and parallelization using OpenMP. While these features contribute to the tool's performance, I find these approaches to be straightforward and relatively standard methods for handling large datasets in C++, rather than technically innovative or advanced.The manuscript includes an experiment using a real dataset from the 1000 Genomes Project, where VCF2Dis is compared with fastreeR. The results demonstrate that VCF2Dis produces a more accurate phylogenetic tree. Upon further investigation, the authors determined that the difference arises from the methods used for phylogenetic tree construction, as the distance matrices generated by both tools were identical. I believe this section may be confusing for the reader and requires revision. The main feature of VCF2Dis is its ability to evaluate distance matrices quickly, and I would therefore expect a direct comparison of this step between the two tools. The observed differences in phylogenetic trees and the more accurate results obtained using VCF2Dis could instead be presented as supporting evidence for why the ape package was chosen as the phylogenetic method in VCF2Dis. However, I would argue that a single case is not sufficient to demonstrate the superiority of ape over the method used in fastreeR. Additional experiments and comparisons are necessary to substantiate this claim.That said, the manuscript serves as a solid technical paper. It identifies a genuine problemâ€"computing distance matrices for large datasetsâ€"presents an efficient solution, and provides a fair comparison with existing tools to demonstrate its superiority. While its current contribution may not be sufficient for a full research article, it could still hold value as a Technical Note with some additional experiments or analyses to enhance its impact.

Level of Interest

Please indicate how interesting you found the manuscript: Choose an item.

Quality of Written English

Please indicate the quality of language in the manuscript: Choose an item.

Declaration of Competing Interests

Please complete a declaration of competing interests, considering the following questions:

- Have you in the past five years received reimbursements, fees, funding, or salary from an organisation that may in any way gain or lose financially from the publication of this manuscript, either now or in the future?
- Do you hold any stocks or shares in an organisation that may in any way gain or lose financially from the publication of this manuscript, either now or in the future?
- Do you hold or are you currently applying for any patents relating to the content of the manuscript?
- Have you received reimbursements, fees, funding, or salary from an organization that holds or has applied for patents relating to the content of the manuscript?
- Do you have any other financial competing interests?
- Do you have any non-financial competing interests in relation to this paper?

If you can answer no to all of the above, write 'I declare that I have no competing interests' below. If your reply is yes to any, please give details below.

I declare that I have no competing interests

I agree to the open peer review policy of the journal. I understand that my name will be included on my report to the authors and, if the manuscript is accepted for publication, my named report including any attachments I upload will be posted on the website along with the authors' responses. I agree for my report to be made available under an Open Access Creative Commons CC-BY license (http://creativecommons.org/licenses/by/4.0/). I understand that any comments which I do not wish to be included in my named report can be included as confidential comments to the editors, which will not be published.

Choose an item.

To further support our reviewers, we have joined with Publons, where you can gain additional credit to further highlight your hard work (see: https://publons.com/journal/530/gigascience). On publication of this paper, your review will be automatically added to Publons, you can then choose whether or not to claim your Publons credit. I understand this statement.

Yes Choose an item.
