# Supplementary material for: VCF2Dis: an ultra-fast and efficient tool to calculate pairwise genetic distance and construct population phylogeny from VCF files
Source: Gigascience. 2025 Apr 4;14:giaf032. doi: 10.1093/gigascience/giaf032 (PMC11970368; doi:10.1093/gigascience/giaf032)
Supplement: giaf032_Reviewer_2_Report_Revision_1 — Catia Vaz -- 1/28/2025 [file giaf032_reviewer_2_report_revision_1.pdf]

Reviewer Report

Title: VCF2Dis: an ultra-fast and efficient tool to calculate pairwise genetic distance and construct population phylogeny from VCF files

Version: Revision 1 **Date:** 1/28/2025

Reviewer name: Catia Vaz

Reviewer Comments to Author:

It thank to the authors for their answers, but I have still some doubts.When authors say "For memory efficiency, we employed a strategy by reading and processing in a manner of line-by-line, ensuring that memory usage is affected only by the number of samples and remains independent of the number of variants. For runtime efficiency, the distance complexity is O(n^2 M) ".I would like to see more details on the paper (perhaps a pseudo-code) of how you in runtime depend on the number of variants but in memory you don't depend. You don't keep it in memory? If so, how you process the distance depending on that? That should be more clarified in my opinion.Also, when you refer:" is not solely due to the programming language but also its optimized implementation." So, what specific optimizations that you have and others don't that allows to do this affirmation? Again, a pseudo code with some comments will help to see what is the innovation of your implementation. Can we reproduce your tests to experiment with other datasets, namely the comparison of your approach with the other one that you mention? I am sorry, I did not find that to reproduce on the github.I also thank to the authors for making avalable a docker image to test it.

Level of Interest

Please indicate how interesting you found the manuscript: Choose an item.

Quality of Written English

Please indicate the quality of language in the manuscript: Choose an item.

Declaration of Competing Interests

Please complete a declaration of competing interests, considering the following questions:

- Have you in the past five years received reimbursements, fees, funding, or salary from an organisation that may in any way gain or lose financially from the publication of this manuscript, either now or in the future?
- Do you hold any stocks or shares in an organisation that may in any way gain or lose financially from the publication of this manuscript, either now or in the future?
- Do you hold or are you currently applying for any patents relating to the content of the manuscript?

- Have you received reimbursements, fees, funding, or salary from an organization that holds or has applied for patents relating to the content of the manuscript?
- Do you have any other financial competing interests?
- Do you have any non-financial competing interests in relation to this paper?

If you can answer no to all of the above, write 'I declare that I have no competing interests' below. If your reply is yes to any, please give details below.

I declare that I have no competing interests'below.

I agree to the open peer review policy of the journal. I understand that my name will be included on my report to the authors and, if the manuscript is accepted for publication, my named report including any attachments I upload will be posted on the website along with the authors' responses. I agree for my report to be made available under an Open Access Creative Commons CC-BY license (http://creativecommons.org/licenses/by/4.0/). I understand that any comments which I do not wish to be included in my named report can be included as confidential comments to the editors, which will not be published.

Choose an item.

To further support our reviewers, we have joined with Publons, where you can gain additional credit to further highlight your hard work (see: https://publons.com/journal/530/gigascience). On publication of this paper, your review will be automatically added to Publons, you can then choose whether or not to claim your Publons credit. I understand this statement.

Yes Choose an item.
