# Supplementary material for: VCF2Dis: an ultra-fast and efficient tool to calculate pairwise genetic distance and construct population phylogeny from VCF files
Source: Gigascience. 2025 Apr 4;14:giaf032. doi: 10.1093/gigascience/giaf032 (PMC11970368; doi:10.1093/gigascience/giaf032)
Supplement: giaf032_Reviewer_3_Report_Original_Submission — Sankar Subramanian -- 11/13/2024 [file giaf032_reviewer_3_report_original_submission.pdf]

Reviewer Report

Title: VCF2Dis: an ultra-fast and efficient tool to calculate pairwise genetic distance and construct population phylogeny from VCF files

Version: Original Submission **Date:** 11/13/2024

Reviewer name: Sankar Subramanian

Reviewer Comments to Author:

A number of software tools have been developed in the past to perform population genetic and evolutionary analysis using large-scale genomic data in Variant Call Format (VCF). The present study has developed another software program, 'VCF2Dis,' which receives a VCF file, computes pairwise genetic distance and infers population phylogeny using simple methods such as Neighbour-Joining and UPGMA methods. While this program has some small incremental benefits over the other available software, such as VCF2POPTREE and fastreeR, it does have limitations that the other software don't. Therefore, I don't think this manuscript has merits that warrant publication in GigaScience, but it may be suitable for other specialized journals.The authors highlight the positive features of VCF2Dis, such as faster execution time compared to VCF2POPTREE and fastreeR. They failed to mention the limitations in comparison with the same software. For instance, VCF2POPTREE is a client-side browser program (no need for installation), and hence, it is easy to draw a phylogeny in a couple of clicks. On the other hand, VCF2Dis is a command line-based program, which has to be complied, installed, and run through a terminal that are not user-friendly, particularly for those who have limited computational skills. Hence, I suggest the authors list the limitations of their software as well. This will inform the users to choose the right software tool that serves their needs.Lines 57-58 states that VCF2POPTREE can analyze only <1500 genomes. Lines 120-121 say that fastreeR also crashes when the number of genomes (or samples) is >1000. However, the performance of VCF2Dis was compared only with that of fastreeR but not with that of VCF2POPTREE using 1000 genomes. The reason for this was stated as VCF2POPTREE crashes when the number of genomes was large (lines 116-117). This statement was contradictory to that mentioned in lines 57-58, which means that VCF2POPTREE is capable of analysing 1000 genomes. I suggest that the authors compare the performances of both VCF2POPTREE and fastreeR with VCF2Dis using 1000 or less genomes.

Level of Interest

Please indicate how interesting you found the manuscript: Choose an item.

Quality of Written English

Please indicate the quality of language in the manuscript: Choose an item.

Declaration of Competing Interests

Please complete a declaration of competing interests, considering the following questions:

- Have you in the past five years received reimbursements, fees, funding, or salary from an organisation that may in any way gain or lose financially from the publication of this manuscript, either now or in the future?
- Do you hold any stocks or shares in an organisation that may in any way gain or lose financially from the publication of this manuscript, either now or in the future?
- Do you hold or are you currently applying for any patents relating to the content of the manuscript?
- Have you received reimbursements, fees, funding, or salary from an organization that holds or has applied for patents relating to the content of the manuscript?
- Do you have any other financial competing interests?
- Do you have any non-financial competing interests in relation to this paper?

If you can answer no to all of the above, write 'I declare that I have no competing interests' below. If your reply is yes to any, please give details below.

I declare that I have no competing interests

I agree to the open peer review policy of the journal. I understand that my name will be included on my report to the authors and, if the manuscript is accepted for publication, my named report including any attachments I upload will be posted on the website along with the authors' responses. I agree for my report to be made available under an Open Access Creative Commons CC-BY license (http://creativecommons.org/licenses/by/4.0/). I understand that any comments which I do not wish to be included in my named report can be included as confidential comments to the editors, which will not be published.

Choose an item.

To further support our reviewers, we have joined with Publons, where you can gain additional credit to further highlight your hard work (see: https://publons.com/journal/530/gigascience). On publication of this paper, your review will be automatically added to Publons, you can then choose whether or not to claim your Publons credit. I understand this statement.

Yes Choose an item.
